# A donor–acceptor photosensitizer-catalyst dyad for light-driven nicotinamide hydrogenation

**DOI:** 10.1039/d5sc08675b

**Published:** 2025-12-23

**Authors:** Alexander Tombrink, Mohini Semwal, Tamar Maisuradze, Alexander K. Mengele, Daniel Straub, Alexander J. C. Kuehne, Sven Rau, Stephan Kupfer, Benjamin Dietzek-Ivanšić, Birgit Esser

**Affiliations:** a Institute of Organic Chemistry II and Advanced Materials, Ulm University Albert-Einstein-Allee 11 89081 Ulm Germany birgit.esser@uni-ulm.de; b Leibniz Institute of Photonic Technology, Research Department Functional Interfaces Albert-Einstein-Str. 9 07745 Jena Germany benjamin.dietzek@uni-jena.de; c Institute of Physical Chemistry, Friedrich Schiller University Jena Helmholtzweg 4 07743 Jena Germany; d Institute of Inorganic Chemistry I, Ulm University Albert-Einstein-Allee 11 89081 Ulm Germany; e Institute of Organic Chemistry III, Ulm University Albert-Einstein-Allee 11 89081 Ulm Germany; f Leibniz-Institut für Oberflächenmodifizierung e.V. (IOM) Permoserstraße 15 04318 Leipzig Germany

## Abstract

Using light energy to drive chemical transformations is of great relevance, with photosynthesis in nature as a grand example. In artificial light-driven catalysis, part of nature's complex supramolecular architecture can be mimicked through the so-called covalently linked photosensitizer-catalyst (PS-CAT) dyads. We herein report a dyad using an organic donor–acceptor PS, with dipyridophenazine as the acceptor and *tert*-butylcarbazole as the donor (2^*t*^BuCzDPPZ), that contains a coordination site for a rhodium(iii)Cp* center as the catalyst. The organic PS shows a charge-transfer transition upon visible-light irradiation and has redox properties similar to typically used ruthenium-based PSs. The resulting PS-CAT dyad 2^*t*^BuCzDPPZRhCp* shows – with methoxy-substituted 1,3-dimethyl-2-phenyl-2,3-dihydro-1*H*-benzo[*d*]imidazole (BIH-OMe) as the sacrificial electron donor – photocatalytic activity in light-driven NAD^+^ reduction with a TON of 3.2 (after 4 h). Femtosecond transient absorption and resonance Raman spectroscopy, as well as time-dependent density functional theory (TDDFT) calculations, shed light on the photophysical properties of the PS and PS-CAT dyad and reveal a high dependency of the photoluminescence quantum yield and excited state properties on solvent polarity – in line with its donor–acceptor structure. This work presents a new design concept for PS-CAT dyads in artificial light-driven catalysis and provides important insight into the interplay between solvation dynamics of organic donor–acceptor systems and their photophysics, paving the way for future design strategies.

## Abstract 2

This work presents a novel photosensitizer-catalyst dyad containing a noble metal-free donor acceptor photosensitizer, which can be employed in light-driven nicotinamide hydrogenation.

## Graphical abstract

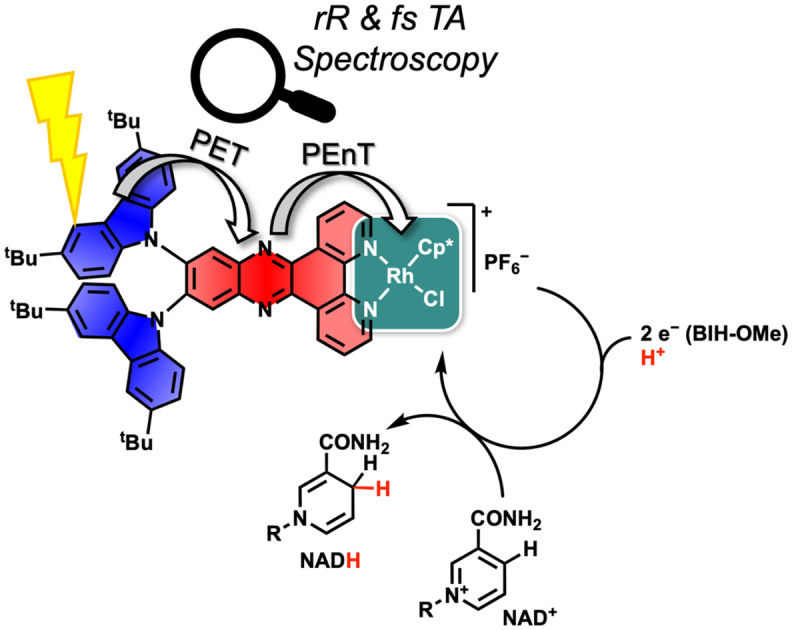

## Introduction

**Fig. 1:**
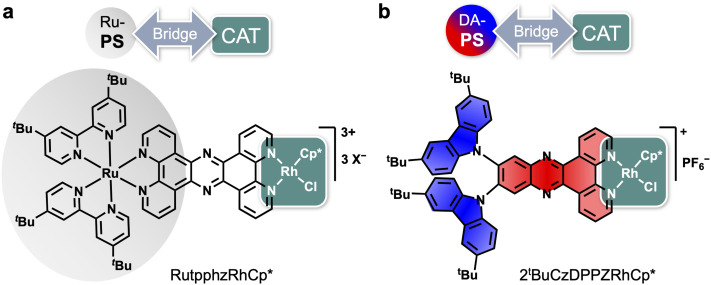
(a) Previously reported Ru-based dyad RutpphzRhCp* and (b) design of the donor–acceptor (D–A)-based photosensitizer (PS)-catalyst (CAT) dyad 2^*t*^BuCzDPPZRhCp*.

Nature's photosynthesis is arguably one of the most significant chemical transformation processes on earth, and it is consequently appealing for scientists to artificially recreate specific core processes in the laboratory.^1^ The interplay of light-harvesting and cascade-like energy transfer, photoinduced charge separation and catalytic turnover processes are of particular interest in this context, and researchers strive to exploit them for artificial systems. In homogeneous artificial light-driven catalysis, typically multicomponent systems are used, containing a photosensitizer (PS) and catalyst (CAT), among others, where the photoinduced electron transfer (PET) between these components is a key step in the photocatalytic cycle. Here, being of intermolecular nature, the PET is limited by the diffusional colliding of PS and CAT or sacrificial agents during the excited-state lifetime of the PS.^2,3^ By contrast, in nature a network of precisely positioned molecular cofactors within photosynthetic proteins efficiently couples light-harvesting, electron transfer and chemical transformation within a complex supramolecular architecture.^4^ Part of this complex assembly can be mimicked through the so-called PS-CAT dyads, in which the photosensitizer and the catalytically active center are covalently linked *via* a bridging unit.^5–7^ An example is the ruthenium-based PS [Ru(tbbpy)_2_(tpphz)]^2+^ (Rutpphz; tbbpy = 4,4′-di-*tert*-butyl-2,2′-bipyridine, tpphz = tetrapyrido[3,2-*a*:2′,3′-*c*:3″,2″-*h*:2‴,3‴*j*]phenazine), which provides a second α-diimine coordination site to attach various types of metal centers to form the respective PS-CAT architectures (Fig. 1a).^8^ By coordinating PdCl_2_, PtCl_2_, or PtI_2_, these dyads showed activity in the hydrogen-evolution reaction (HER) with TONs (turn-over numbers) of up to 276 after 70 h.^5,9^ By altering the catalytic center to RhCp*Cl (Cp*: 1,2,3,4,5-pentamethylcyclopentadienyl), the resulting dyad RutpphzRhCp* not only showed photocatalytic activity in the HER, but also in the reduction of the nicotinamide co-factor NAD^+^ with TONs up to 6 after 90 min.^7,10^

A disadvantage of these dyads is the use of a Ru-based complex as a PS, containing a rare and expensive noble transition metal. Limited options for chemical modifications and low stability can also be problematic. Using organic chromophores as a PS that consist of abundant elements is an attractive goal, and has been realized on few occasions in PS-CAT dyads.^6,11–14^ Since the main first step in the light-driven catalysis of the Ru-based dyads is a ^1^MLCT population upon photon absorption, we searched for a suitable replacement in the form of an organic donor–acceptor (D–A) compound that also shows a vectorial charge-transfer transition upon visible-light irradiation and contains a coordination site for the CAT moiety. We choose a Rh(iii)Cp* center as a model CAT as it offers a broad variety of known catalytic functions^15–18^ and has been investigated with a multitude of spectroscopic tools.^10,19–21^ The above mentioned coordination site can easily be employed for alternative CAT centers like PtI_2_ or PdCl_2_. We identified 2^*t*^BuCzDPPZ (bis(di-*tert*-butylcarbazole)dipyridophenazine), reported by Morgan *et al.*, to match our requirements (blue/red structure in Fig. 1b). 2^*t*^BuCzDPPZ is an organic red-light-emitting compound with a high molar absorptivity, featuring a dipyrido unit to attach RhCp* as the catalytic center.^22^ In addition, its reported redox properties fit to earlier reported Ru-polypyridyl centers, as the oxidation potential of 0.94 V lies close to the oxidation of Ru^II^ to Ru^III^ at around 0.8 V (all redox potentials given *versus* the ferrocene/ferrocenium redox couple (Fc/Fc^+^)). Also, the reported reduction potential of −1.59 V is close to the reduction of the bridging ligand at −1.44 V of the Ru-dyad.^10,22^ Furthermore, the dipyridophenazine coordination sphere provides an attractive interaction between the CAT-based metal center and the extended π-ligand system.^23,24^ This makes 2^*t*^BuCzDPPZ highly desirable as a purely organic PS for the novel PS-CAT dyad 2^*t*^BuCzDPPZRhCp* (Fig. 1b).^10^

We herein set out to synthesize and investigate the photocatalytic activity of the new PS-CAT dyad 2^*t*^BuCzDPPZRhCp*. We report its excited-state photophysics, in comparison to 2^*t*^BuCzDPPZ, using resonance Raman (rR) spectroscopy, femtosecond transient absorption spectroscopy, and quantum chemical calculations (DFT and TDDFT as well as TDA-TDDFT) including scalar-relativistic effects as well as spin–orbit couplings, revealing strong solvent-dependent variations in the triplet-state character and lifetimes. We demonstrate that 2^*t*^BuCzDPPZRhCp* – with a suitable sacrificial electron donor – shows photocatalytic activity in the light-driven NAD^+^ reduction. This work presents a new design concept for PS-CAT dyads in artificial light-driven catalysis since the TADF-like chromophore 2^*t*^BuCzDPPZ features the crucial property of directional light-induced electron/energy transfer within the PS towards the CAT entity. The understanding of the deactivation mechanism and proof of photocatalytic competence opens a pathway to improved properties in artificial photosynthesis.

## Results and discussion

### Synthesis

**Scheme 1:**
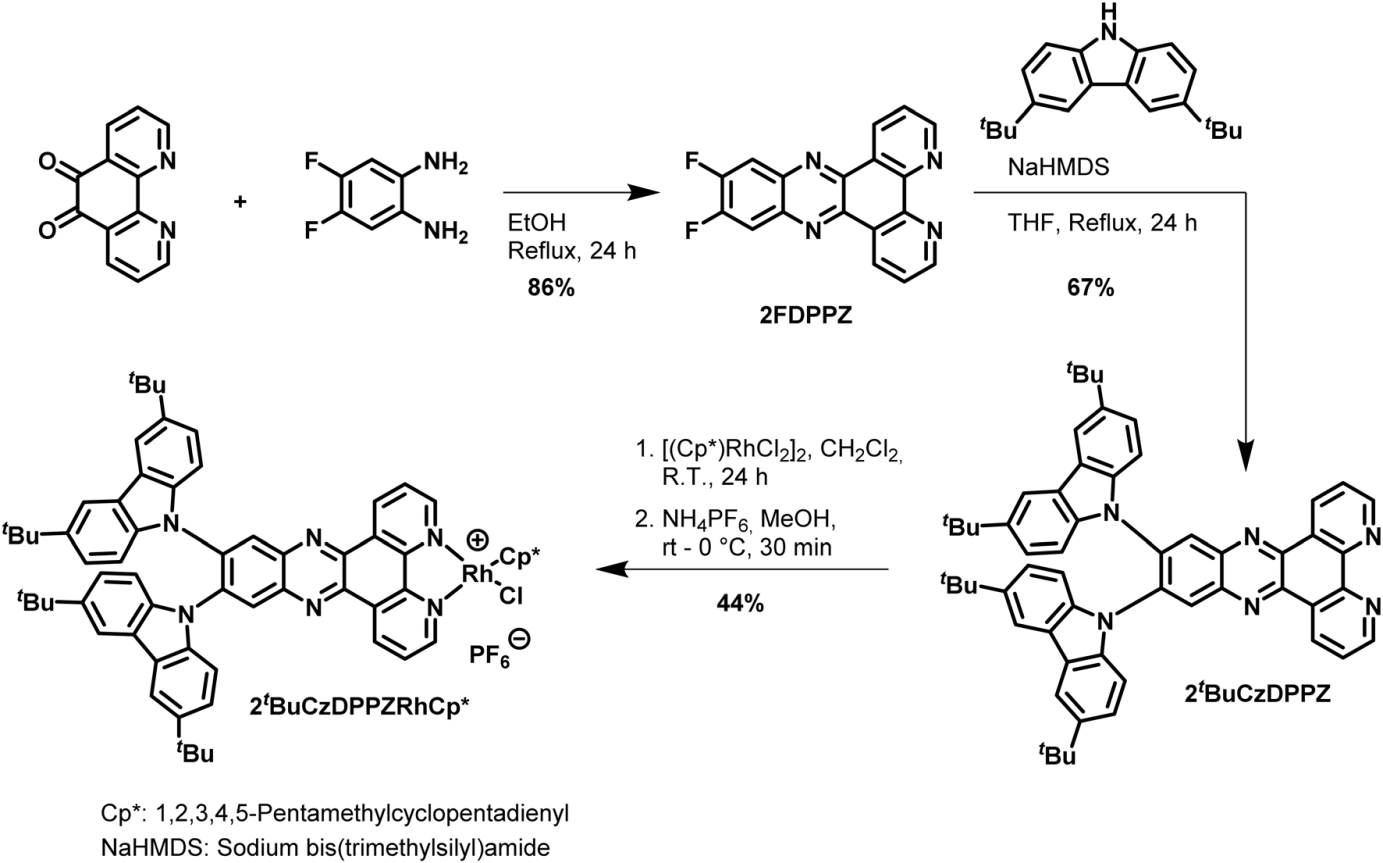
Synthesis of the D–A-photosensitizer/ligand 2^*t*^BuCzDPPZ and its Rh complex 2^*t*^BuCzDPPZRhCp* as a PS-CAT dyad.

2^*t*^BuCzDPPZ was synthesized in three steps in analogy to the literature^22^ starting with a condensation reaction^25^ between 1,10-phenanthroline-5,6-dione and 2-amino-4,5-difluoroaniline to form 2FDPPZ (Scheme 1) complementing the π-system of the chromophore. In a subsequent nucleophilic aromatic substitution^22^ the two 3,6-di-*tert*-butyl-9*H*-carbazoles (^*t*^BuCz) were attached, furnishing 2^*t*^BuCzDPPZ. The PS-CAT dyad 2^*t*^BuCzDPPZRhCp* was obtained by reaction of the ligand with the pentamethylcyclopentadienyl rhodium(iii)-dichloride dimer.^11^

We also synthesized further modifications of the D–A-based PS-CAT dyad by varying the donor type and position (Scheme S1). The synthesis of the *para*-isomer *p*2^*t*^BuCzDPPZRhCp* started with a Buchwald–Hartwig amination of 4,7-dibromobenzo[*c*][1,2,5]thiadiazole with ^*t*^BuCz (see the SI for details). A reduction of the thiadiazole with LiAlH_4_ gave the respective diamine, which was directly condensed with 1,10-phenanthroline-5,6-dione to the final ligand *p*2^*t*^BuCzDPPZ. This was then reacted with the pentamethylcyclopentadienyl rhodium dichloride dimer to form *p*2^*t*^BuCzDPPZRhCp*.^11^ For the third dyad 2TPADPPZRhCp*, 1,10-phenanthroline-5,6-dione was condensed with 4,5-dibromo-*o*-phenylenediamine to form 11,12-dibromodipyridophenazine, which was converted to 2TPADPPZ by Suzuki–Miyaura coupling with 4-diphenylaminophenylboronic acid (Scheme S1). Reaction with the pentamethylcyclopentadienyl rhodium dichloride dimer afforded 2TPADPPZRhCp*.^11^

All compounds were characterized by NMR spectroscopy and high-resolution mass spectrometry (see SI Fig. S1–S24).

In the following section, we report the detailed optoelectronic and photophysical characterization and catalyst optimization of the PS-CAT dyad 2^*t*^BuCzDPPZRhCp* as well as the ligand 2^*t*^BuCzDPPZ. *p*2^*t*^BuCzDPPZRhCp* and 2TPADPPZRhCp* were not considered further, as *p*2^*t*^BuCzDPPZRhCp* features only a weak charge-transfer band in the UV-vis spectrum at *ca.* 600 nm with a low extinction coefficient (Fig. S54a), while for 2TPADPPZRhCp* initial photocatalysis tests revealed a low performance in NAD^+^ reduction (Fig. S54b).

### Photophysics

We first investigated the optoelectronic properties of 2^*t*^BuCzDPPZ and 2^*t*^BuCzDPPZRhCp* to gain insight into their absorption characteristics and excited-state relaxation dynamics. Cyclic voltammetry (CV) in CH_2_Cl_2_ solution confirms a D–A character for 2^*t*^BuCzDPPZ with oxidation half-wave potentials of 0.74 V and 1.06 V (all redox potentials are given *versus* the ferrocene/ferrocenium redox couple (Fc/Fc^+^)) and a reduction half-wave potential of −1.70 V (Table 1 and SI, Fig. S25). The oxidations take place at the *tert*-butyl carbazoles,^26^ and the reduction occurs on the DPPZ core. In the PS-CAT dyad 2^*t*^BuCzDPPZRhCp*, the potentials are slightly shifted, and, in addition, new irreversible processes were observed at 1.13, −1.28, and −0.22 V. However, the wave at −0.22 V was only visible when both the oxidative and reductive areas were scanned. The two-electron reduction of the Rh center (Rh^III^/Rh^I^) was observed at −1.21 V, which lies in the range of values reported in the literature.^10,11^ This indicates that after excitation the *tert*-butyl carbazoles can be re-reduced with common sacrificial electron donors (SEDs), since the oxidation potential of the SED is lower (*e.g. E*_ox_ (NEt_3_): 0.31 V *vs.* Fc/Fc^+^)^27^ than *E*_1/2-ox_ of 2^*t*^BuCzDPPZRhCp*.

**Table 1:** Redox potentials of the PS-CAT dyad and its components (in CH_2_Cl_2_ (1 mM) with 0.1 M *n*-Bu_4_NPF_6_)

Compound	*E*_1/2-ox_ *vs.* Fc/Fc^+^/V	Peak-to-peak separation^a^/V	*E*_1/2-red_ *vs.* Fc/Fc^+^/V	Peak-to-peak separation^a^/V
^*t*^BuCz^26^	0.78^b^	—	—	—
DPPZ^28^	—	—	−1.6	—
			−2.06	
2^*t*^BuCzDPPZ	0.74	0.31	−1.70	0.19
	1.06	0.16		
2^*t*^BuCzDPPZRhCp*	0.81	0.09	−0.22^c^^,^^e^	0.21
	1.13^c^^,^^d^	—	−1.21	0.40^f^
	1.21	0.73	−1.28^c^^,^^e^	—
			−1.73	—

a Peak-to-peak separation for Fc/Fc^+^ in the measurement: 0.45 V (2^*t*^BuCzDPPZ) and 0.35 V (2^*t*^BuCzDPPZRhCp*).

b From the literature-reported value of 1.12 V *vs.* SCE, 0.38 V was subtracted to obtain the value *vs.* Fc/Fc^+^.^29^

c Irreversible processes.

d Anodic peak potential.

e Cathodic peak potential.

f Quasi-reversible.

**Fig. 2:**
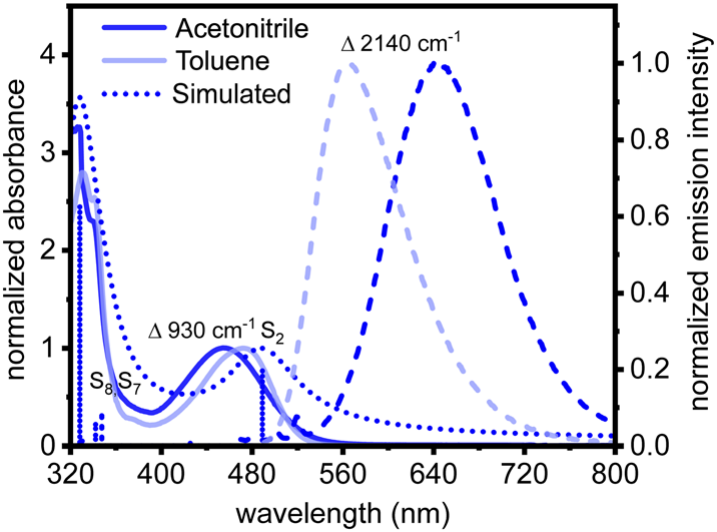
Electronic absorption (solid lines) and emission (dashed lines; *λ*_exc_ = 450 nm) spectra of 2^*t*^BuCzDPPZ in acetonitrile (blue) and toluene (light blue) together with its simulated absorption spectra (dotted lines, blue).

The ground-state absorption spectrum of 2^*t*^BuCzDPPZ in toluene features an absorption band centered at 472 nm (Fig. 2). Quantum chemical calculations associate this absorption feature with a dipole-allowed intramolecular charge transfer excitation of 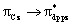 character into the S_2_ state with an excitation wavelength of 499 nm (2.47 eV, Table S7). This band undergoes a hypsochromic shift to 452 nm in acetonitrile (*Δ* = −930 cm^−1^) without altering the character of the underlying electronic transition (S_2_ at 490 nm, 2.54 eV, Table S3). Notably, the transition into the S_1_ charge transfer state is dipole-forbidden (*i.e.* optically inactive) in both solvents. The emission maximum shifts from 563 nm in toluene to 645 nm in acetonitrile (*Δ* = 2140 cm^−1^). Such a red shift in a polar solvent is characteristic for CT emitters and suggests a stabilization of the lowest excited singlet state (S_1_) due to its strong dipole moment.^30,31^

A comparison of the fluorescence and phosphorescence spectra (recorded at 77 K in methylcyclohexane) provides a relatively small energy gap (Δ*E*_ST_ = 0.15 eV) between the first excited singlet and triplet states (Fig. S34 and Table S1). According to quantum chemical calculations at the Franck–Condon point, the vertical S_1_–T_1_ energy gaps are 0.25 and 0.26 eV in toluene and acetonitrile, respectively. In the case of toluene, the energy gap between the fully relaxed S_1_ and T_1_ states was predicted to be 0.25 eV, *i.e.* identical to the Δ*E*_ST_ in the Franck–Condon geometry (S_0_ equilibrium structure). Therefore, the subsequently discussed singlet-triplet energy gaps are exclusively obtained based on the vertical energy difference in the relaxed singlet ground state. Calculated rates of intersystem crossing (ISC) (*k*_ISC_, SI, eqn (S1)) between lower lying singlet (S_1_ and S_2_) and triplet (T_1_ and T_2_) states, as well as rates of spontaneous emission (*k*_F_, SI, eqn (2)) were obtained in both acetonitrile and toluene and indicated rather slow and inefficient population transfer among low-lying singlet and triplet states as well as slow (spontaneous) emission based on the quasi dipole-forbidden S_1_ → S_0_ transition (Table S2).

The photoluminescence quantum yield (PLQY) of 2^*t*^BuCzDPPZ is strongly solvent-dependent, as typically observed for CT-emitters, where the excited CT state is stabilized in more polar solvents (Table 2): whereas in toluene the PLQY amounts to 36%, in acetonitrile it is only 2% (both argon-purged). In non-argon-purged (aerated) solvents the PLQYs are slightly lower (29% in toluene and 1% in acetonitrile). A strong increase in PLQY upon argon-purging of a dye solution typically indicates participation of a triplet excited state. Furthermore, a strong solvent (polarity) effect on PLQYs and on the excited-state properties is typical for photophysical properties of DPPZ-like scaffolds.^53,54^

**Table 2:** Photophysical properties of 2^*t*^BuCzDPPZ and 2^*t*^BuCzDPPZRhCp*

	2^*t*^BuCzDPPZ		2^*t*^BuCzDPPZRhCp*	
	Air-saturated	Argon-purged	Air-saturated	Argon-purged
PLQY/% (acetonitrile)	1	2	1	1
PLQY/% (toluene)	29	36	10	12
*τ*/ns (acetonitrile)	0.10, 3.87^a^	0.10, 4.87^a^	0.28, 1.42^a^	0.34, 1.91^a^
*τ*/ns (toluene)	8.63^b^	9.74^b^	0.09, 5.34^a^	0.15, 8.74^a^

a Bi-exponential decay.

b Mono-exponential decay.

**Fig. 3:**
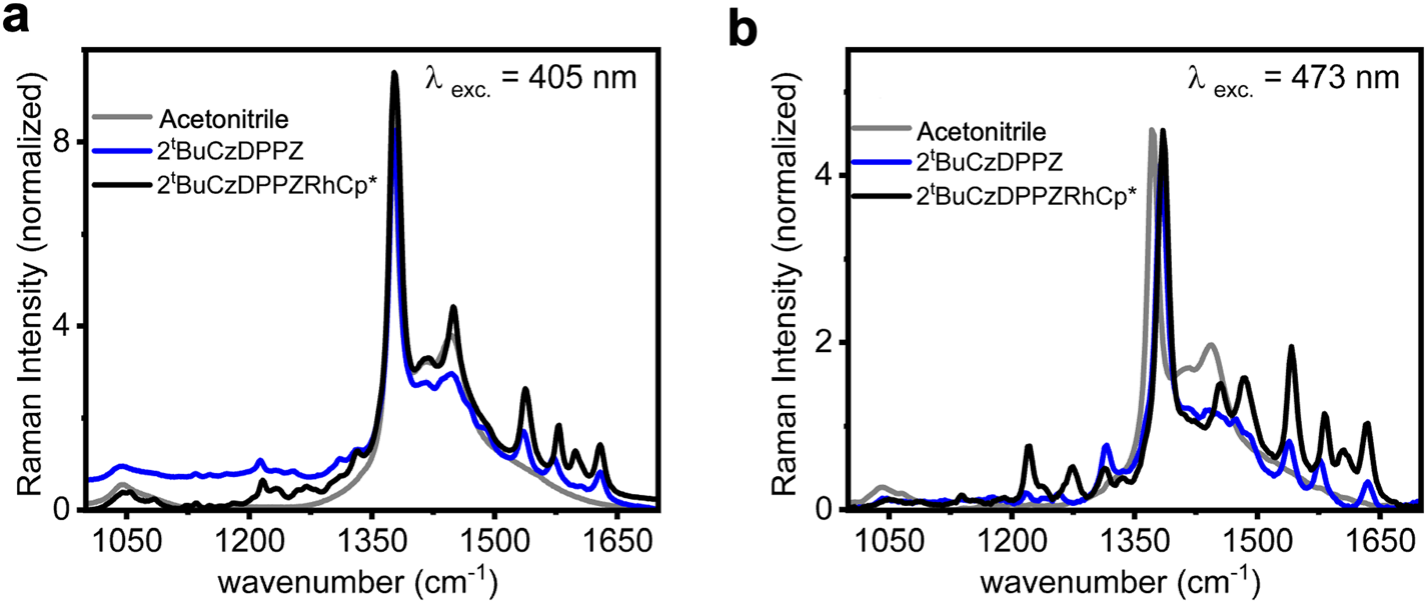
Resonance Raman spectra of 2^*t*^BuCzDPPZ and 2^*t*^BuCzDPPZRhCp* in acetonitrile excited at (a) 405 nm and (b) 473 nm. All spectra are recorded in solution, normalized to the solvent band (acetonitrile) at 1370 cm^−1^ and with respect to the absorption coefficient of the corresponding excitation wavelength.

To further characterize the Franck–Condon point of 2^*t*^BuCzDPPZ and 2^*t*^BuCzDPPZRhCp*, the resonance Raman (rR) spectra of both compounds were measured in their acetonitrile solution. The rR selectively enhances vibrations of the ligands that are involved in the electronic transitions, and enables an understanding of the localization of the initially excited state of the chromophore. The rR spectra recorded upon 405 nm excitation are shown in Fig. 3. Both for 2^*t*^BuCzDPPZ and 2^*t*^BuCzDPPZRhCp* the Raman spectrum is dominated by sharp bands at 1536, 1577, 1602, and 1630 cm^−1^, along with a shoulder at 1487 cm^−1^ (Fig. 3a). These features are attributed to C=C and C=N stretching vibrations of the DPPZ bridging ligand, indicating π–π* or intramolecular CT transitions centered on the DPPZ chromophore.^32,33^ Carbazole contributions at this excitation energy are minimal. Upon 473 nm excitation, a broader set of vibrational features emerges, including DPPZ-associated modes at 1541, 1582, 1606, and 1633 cm^−1^.^34^ Finally, the appearance of weak bands at 1219, 1271, 1315, 1455, and 1486 cm^−1^ points to the involvement of the carbazole moieties.^35,36^ The latter spectral pattern aligns well with Raman bands of free carbazole in dichloromethane, which are assigned to the in-plane bending and C–N/C=C stretching modes of the carbazole framework.^35,37,38^ This suggests that 405 nm excitation leads to CT transitions (from the carbazoles to DPPZ). Upon longer-wavelength excitation at 473 nm, the intensity ratio of carbazole with the DPPZ ligand increases as compared to that at 405 nm (Fig. 3b; the Raman bands were normalized with respect to their absorption coefficient). The enhanced intensity of carbazole-based vibrational modes under 473 nm excitation suggests that this excitation involves the π-systems of both the terminal carbazole and the central DPPZ ligand. Overall, these results demonstrate that 405 nm excitation selectively enhances DPPZ-localized vibrations, while 473 nm excitation activates modes from both DPPZ and carbazole.

**Fig. 4:**
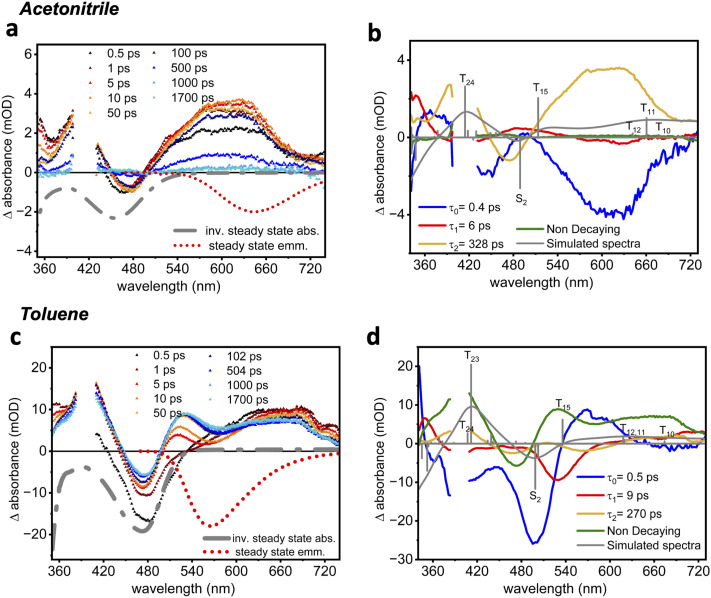
(a and c) Transient absorption spectra of 2^*t*^BuCzDPPZ in acetonitrile (a) and toluene (c) following excitation at 400 nm. (b and d) Decay-associated spectra (DAS) in the wavelength range of 350 to 740 nm with its simulated TD (DFT) calculated spectra in acetonitrile (b) and toluene (d).

To gain insights into the excited-state dynamics of 2^*t*^BuCzDPPZ, femtosecond transient absorption (fs-TA) measurements were performed upon excitation at 400 nm (Fig. 4a). In acetonitrile, a pronounced ground-state bleach (GSB) is observed at 450 nm, consistent with the inverse of the steady-state absorption spectrum (Fig. 2a). In parallel, excited-state absorption (ESA) bands emerge at both short (360–400 nm) and long (>500 nm) probe wavelengths (Fig. S26 and S27). The long-wavelength ESA is assigned to an intramolecular CT transition from one carbazole unit to the phenazine core. Upon photoexcitation, both ESA bands rise rapidly, *i.e.* within the first 0.5 ps to 50 ps, followed by a decay onsetting after approximately 100 ps. As a result of this decay, approximately 95% of the initial excited-state population decays within 1 ns. The lifetime associated with the sub-ns decay is reminiscent of the fast, 0.1 to few ns component in the emission lifetime measurements (see Table 2).

Global kinetic analysis was performed using three decay components in addition to a non-decaying (within our experimental window of 1.8 ns) component to reasonably fit the TA signal (see Fig. 4). The resulting decay-associated spectra (DAS, Fig. 4b and d) show that the first two components, associated with the decay time constants *τ*_0_ = 0.4 ps and *τ*_1_ = 6 ps, correspond to ultrafast vibrational and electronic relaxations, respectively, within the initially populated ^1^CT state. According to DFT, the fully relaxed T_1_ state features a prominent CT character in acetonitrile which is associated with a charge redistribution from the π-system of the carbazole moieties towards the lowest energy 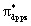 orbital. Excitation of this T_1_ state causes the ESA signal observed in the time-resolved measurements. More precisely, simulations of the TA spectra indicated that a 1 : 3 singlet-to-triplet ratio best accounts for the experimental data. According to TDA-TDDFT, the main contributions to the ESA stem from T_24_, T_15_, and T_12–10_, resulting in major absorption at ∼425 nm, ∼520 nm, and 630–680 nm, respectively (Fig. S49 and Table S6). Excitations into T_10,11_ and T_24_ feature mixed CT and locally excited characters, while T_12_ is of pure CT and T_15_ is of 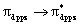 nature. Detailed information with respect to the electronic nature of the underlying transitions is collected and provided in the SI, as shown in Table S1. From this we assign the 328 ps component (*τ*_2_) to the decay of a long-lived ^3^CT state, while the long-lived non-decaying component is nearly featureless and may represent a non-decaying offset or minor contribution from a weakly emissive state.

2^*t*^BuCzDPPZ in the nonpolar solvent toluene does not show the same results (Fig. 4d): within the first few picoseconds, an ESA band centered at around 650 nm is observed, mirroring the early-time spectral feature in acetonitrile. However, at later delay times (after *ca.* 5 ps), the ESA feature at 520 nm progressively intensifies, while the ESA at 620 nm decays. This behavior leads to the appearance of an isosbestic point at 590 nm, which indicates internal conversion between two states, assigned to simulated T_11_ and T_15_ states (Table S10). According to TDA-TDDFT, the nature of the transition into T_11_ in toluene shifts from partial CT in acetonitrile to purely CT in toluene, while the respective transition dipole moment decreases significantly. In contrast, the CT nature of T_15_ remains unaffected. These solvent-dependent relaxation channels – as evident by means of the difference in the ESA spectral profile – highlight the influence of solvent polarity on the excited-state dynamics, especially impacting the triplet state behavior and interconversion of the involved CT states.

**Fig. 5:**
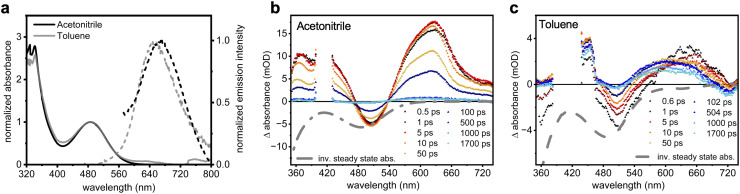
(a) Electronic absorption (solid lines) and emission (dashed lines; *λ*_exc._ = 450 nm) spectra of 2^*t*^BuCzDPPZRhCp* in acetonitrile (black) and toluene (grey). (b and c) Transient absorption spectra of 2^*t*^BuCzDPPZ in acetonitrile (b) and toluene (c) following excitation at 400 nm.

We next investigated the photophysical properties of the PS-CAT dyad 2^*t*^BuCzDPPZRhCp* in more detail. Upon coordination of an Rh center to form the dyad 2^*t*^BuCzDPPZRhCp*, the intramolecular charge-transfer absorption further red-shifts to 492 nm compared to 2^*t*^BuCzDPPZ, independent of solvent polarity (Fig. 5a). According to the TDA-DFT calculations with the range-separated ωB97x-d3 functional as well as using the PBE0 global hybrid functional, this band can be consistently associated with a dipole allowed CT 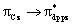 transition into the S_2_, state (Table S1). However, the excitation energies are over- and underestimated, respectively, depending on the functional used, thus a further assignment of the subsequent excited-state processes in the PS-CAT dyad based on quantum chemical simulations was – unfortunately – not possible. The emission maxima of 2^*t*^BuCzDPPZRhCp* are observed at 647 nm in toluene and at 672 nm in acetonitrile, which correspond to only a small bathochromic shift in the more polar solvent (*Δ* = 575 cm^−1^). This red shift is three times smaller than that in 2^*t*^BuCzDPPZ, indicating a notable impact of the Rh center on the excited states associated with the phenazine core.

Notably, the introduction of the Rh center significantly decreases the PLQY compared to the photosensitizer 2^*t*^BuCzDPPZ (Table 2). The PLQY drops from 36% (2^*t*^BuCzDPPZ) to 12% (2^*t*^BuCzDPPZRhCp*) in toluene (under an argon atmosphere), and from 2% to 1% in acetonitrile. This trend is consistent with the observed emission lifetimes (see SI Fig. S36 and S37). In toluene, the lifetime decreases from 9.7 ns (2^*t*^BuCzDPPZ) to 8.7 ns (2^*t*^BuCzDPPZRhCp*), whereas in acetonitrile it decreases from 4.9 ns (2^*t*^BuCzDPPZ) to 1.9 ns (2^*t*^BuCzDPPZRhCp*). These experimental observations point towards excited-state electron transfer from the photosensitizer to the Rh center and are corroborated by calculations of the non-radiative decay rates (*k*_nr_). In toluene, the non-radiative rate increases from 6.6 × 10^7^ s^−1^ for 2^*t*^BuCzDPPZ to 1.0 × 10^8^ s^−1^ for 2^*t*^BuCzDPPZRhCp*—an approximate 50% increase. This effect is even more pronounced in acetonitrile, where *k*_nr_ dramatically increases from 2.0 × 10^8^ s^−1^ (2^*t*^BuCzDPPZ) to 5.2 × 10^8^ s^−1^ (2^*t*^BuCzDPPZRhCp*). These values for *k*_nr_ reflect solvent effects and reveal an increase in non-radiative decay pathways, potentially mediated by the coordinated Rh center. A similar effect has been documented in ruthenium and perylene dyads, and it can be rationalized by the transfer of excited-state electrons to the Rh metal center^7,11^ – a prerequisite for photocatalysis, during which the Rh(iii) center must undergo a twofold reduction *via* PET to a Rh–H species, which then reduces NAD^+^ (see further below).

Femtosecond TA measurements on the dyad 2^*t*^BuCzDPPZRhCp* were performed both in acetonitrile and toluene (Fig. 5b, c and SI S31, S33). Upon coordination with the Rh center, the transient spectral features in acetonitrile resemble those of the PS 2^*t*^BuCzDPPZ, including a characteristic GSB at 505 nm and ESA bands in the regions of 360–400 nm and >500 nm (Fig. 5b). However, in acetonitrile the excited state of 2^*t*^BuCzDPPZRhCp* decays faster than that of 2^*t*^BuCzDPPZ (*cf.*Fig. 4a): the transient signals of 2^*t*^BuCzDPPZRhCp* decay almost completely (to 5% of the initial amplitude) within 92 ps. This stands in stark contrast to the comparably slow excited-state decay of 2^*t*^BuCzDPPZ. The rapid deactivation in 2^*t*^BuCzDPPZRhCp* is attributed to an accelerated photoinduced energy transfer (PEnT) facilitated by the Rh center. This behavior is consistent with prior studies showing enhanced PEnT in heavy-atom-containing systems.^39–41^ Interestingly, in toluene, PEnT seems to be prolonged, with ESA features persisting beyond our experimental window of 2 ns (Fig. 5c). Coordination of Rh significantly alters the excited-state dynamics of the 2^*t*^BuCzDPPZ photosensitizer. While the free PS exhibits two distinct ESA bands in toluene—indicative of internal conversion between triplet excited states (as shown in Fig. 4e)—2^*t*^BuCzDPPZRhCp* displays only a single ESA feature centered at 650 nm (Fig. 5c). This suggests that coordination to Rh simplifies the excited-state manifold so that only a single triplet state remains visible in the TA data. The complete recovery of the ground-state bleach within 1 ns in both solvents further supports the notion that the excited-state population fully relaxes back to the ground state. The polarization dependence of this process and the fact that such fast ground-state recovery is observed upon coordination of the Rh center suggest that the molecular origin of the excited-state relaxation is a PEnT towards the Rh(iii) metal center. This has also been supported by the emission lifetime (see Table 2), where the introduction of Rh significantly reduces the emission lifetime of the 2^*t*^BuCzDPPZ chromophore.

### Photocatalytic performance of the PS-CAT dyad

We next set out to investigate the photocatalytic performance of the new PS-CAT dyad. Prior to photocatalysis we checked the general activity of the catalytic Rh(iii)Cp* center in 2^*t*^BuCzDPPZRhCp* for NAD^+^ reduction in a “thermal” (dark) catalysis.^42,43^ For this, sodium formate was used as the reducing agent. It is anticipated that the dye center (PS) has no influence on the outcome of the thermal catalysis, as reported by Zedler and co-workers.^17,41,44^ They observed a similar performance for three PSs with different bridging ligands to the Rh metal center and a strong temperature dependence in accordance with an Arrhenius plot since the reaction only involved the Rh-metal center and not the bridging ligand or the PS part. In our case we could observe catalytic activity (TON: 8 (after 60 min, 45 °C), TOF: 21 h^−1^ (after 5 min, 45 °C), see SI Fig. S42) under similar conditions, which demonstrates that 2^*t*^BuCzDPPZRhCp* is in principle capable of reducing NAD^+^ to NADH. However, in the literature higher TONs and TOFs were achieved by using different catalysts (*e.g.* RutpphzRhCp*: TON: 43 (after 50 min, 45 °C), TOF: 75 h^−1^ (after 5 min, 45 °C)).^41^ One explanation for this might be the slightly more negative redox potential (100 mV) for the Rh(iii)/Rh(i) couple of the 2^*t*^BuCzDPPZRhCp* dyad in comparison to the Ru-based dyads.^41^ A more negative potential is associated with a lower NAD^+^ reduction rate.^43^ In addition, it has been previously shown for DPPZ-containing Ru(ii) polypyridine complexes that NADH is strongly stacking to the DPPZ moiety of the complex.^45^ This could potentially lead to it being readily available for reoxidation of NADH into NAD^+^ by Rh(iii).

**Fig. 6:**
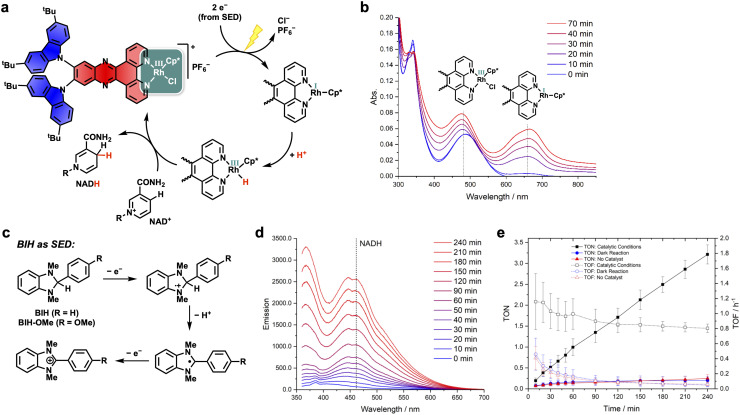
(a) Proposed mechanism of the light-driven NAD^+^ reduction using the PS-CAT dyad 2^*t*^BuCzDPPZRhCp*.^10^ (b) UV/vis spectra of the catalytic mixture of dyad 2^*t*^BuCzDPPZRhCp* (5 µM) with TEA (0.212 M) as an SED (0.17 M NaH_2_PO_4_, LED (*λ*_max_ = 465 nm, fwhm = 20 nm, *P* = 45 mW cm^−2^), acetonitrile/water (1/2, v/v)). (c) Mechanism of BIH serving as the SED.^27^ (d) Formation of NADH monitored by fluorescence spectroscopy with (e) TON and TOF values under the following conditions in acetonitrile/water (1/2, v/v): 5 µM 2^*t*^BuCzDPPZRhCp*, 400 µM BIH-OMe, 250 µM NAD^+^, LED (*λ*_max_ = 465 nm, fwhm = 20 nm, *P* = 45 mW cm^−2^); dark reaction: 5 µM 2^*t*^BuCzDPPZRhCp*, 400 µM BIH-OMe, 250 µM NAD^+^; no catalyst: 400 µM BIH-OMe, 250 µM NAD^+^, LED (*λ*_max_ = 465 nm, fwhm = 20 nm, *P* = 45 mW cm^−2^).

To assess the photocatalytic activity of the new PS-CAT dyad we studied the light-driven reduction of NAD^+^ to NADH. The mechanism of this process starts by exciting 2^*t*^BuCzDPPZRhCp* with LED light (*λ*_max_ = 465 nm, fwhm = 20 nm, *P* = 45 mW cm^−2^), which is assumed to cause a photo-induced electron transfer from the PS to the Rh center (Fig. 6a). This process must occur twice, as the Rh(iii) has to be reduced to Rh(i) under the loss of an anionic ligand. To regenerate the oxidized carbazole, a sacrificial electron donor (SED) is needed. Oftentimes triethylamine (TEA) fulfills the intended purpose, although it must be used in high quantities (24 000 eq. relative to the 2^*t*^BuCzDPPZRhCp* dyad).^10,11^ After the oxidative addition of a proton to the Rh center (red in Fig. 6a), the final hydride transfer from the latter to NAD^+^ can take place to afford NADH.^10^

In our first experiments we tested the well-established conditions with TEA (p*K*_a_ (HNEt_3_^+^): 10.76; *E*_ox_ (NEt_3_): 0.31 V *vs.* Fc/Fc^+^)^27^ as an SED as its redox potential fits for reducing the oxidized carbazoles (*cf.*Table 1).^10,11^ However, with the 2^*t*^BuCzDPPZRhCp* dyad, no NADH was formed under these conditions. *In situ* spectroscopic investigations revealed that during photocatalysis a broad absorption band at 666 nm arises within minutes (Fig. 6b). This can be attributed to the formation of an Rh(i) species, which is an intermediate in the catalytic cycle (Fig. 6a). The formation of this intermediate in a TEA/phosphate buffer could previously only be observed when no NAD^+^ was present but is typically found if dihydrogenphosphate is left out of the solution.^10,11,20^ Its accumulation indicates that the next step of the catalytic cycle is hindered, the oxidative addition of a proton. From this, one can conclude that the p*K*_a_ value of the Rh–H species is lower than the p*K*_a_ value of the buffer solution. Increasing the buffer concentration did not solve this problem. Shifting to other amine-based SEDs with lower p*K*_a_ values was also not successful. Triethanolamine (p*K*_a_ (HN(CH_2_CH_2_OH)_3_^+^): 7.74; *E*_ox_ (N(CH_2_CH_2_OH)_3_): 0.19–0.44 *vs.* Fc/Fc^+^)^27,46^ performed similar to TEA, and the aromatic amines dimethylaniline (p*K*_a_ (HNMe_2_Ph^+^): 5.15; *E*_ox_ (NMe_2_Ph): 0.43 *vs.* Fc/Fc^+^)^27,47^ and *N*,*N*-dimethyl-*p*-toluidine (p*K*_a_ (HNMe_2_(C_6_H_4_Me)^+^): 5.63; *E*_ox_ (NMe_2_(C_6_H_4_Me)): 0.33 *vs.* Fc/Fc^+^)^27,48^ showed neither NADH nor Rh(i) formation. Finally, modified benzimidazole SEDs brought success. 1,3-Dimethyl-2-phenyl-2,3-dihydro-1*H*-benzo[*d*]imidazole (BIH) is well known both as a hydride donor and as an SED (Fig. 6c).^27^ BIH and its derivatives were for instance employed by Ishitani as an SED for the HER and CO_2_ reduction with Ru-RhCp* dyads.^49,50^ The mechanism of action when BIH serves as an SED is shown in Fig. 6c and involves a stepwise oxidation *via* successive one-electron transfer processes as well as an associated deprotonation.^27^ BIH therefore supplies everything needed for the NAD^+^ reduction. Initial tests with this SED showed promising results, although BIH was almost insoluble in the catalytic mixture.

To increase solubility, we attached a methoxy group to the phenyl substituent of BIH, resulting in BIH-OMe.^51^ Being sufficiently soluble in the acetonitrile/water mixture used in photocatalysis, BIH-OMe was finally able to serve as a functional SED in the light-driven NAD^+^ reduction with dyad 2^*t*^BuCzDPPZRhCp*. The amount of NADH was determined by fluorescence spectroscopy (Fig. 6d, *λ*_max_(NADH): 461.5 nm, see SI Fig. S38 and S39). Under the optimized conditions dyad 2^*t*^BuCzDPPZRhCp* delivered NADH with a TON of 3.2 after 4 h and a maximum TOF of 1.2 h^−1^ (after 10 min) (Fig. 6e). For the literature-known Ru-based dyad RutpphzRhCp*, TONs up to 8 (after 60 min) and TOFs of 9 h^−1^ (after 60 min) could be observed.^41^ In the case of the perylene-based dyad, TONs of 4 and TOFs of 4 h^−1^ (after 60 min) were reported.^11^ Despite the lower values of the herein reported dyad, it could be shown that 2^*t*^BuCzDPPZRhCp* works as a proof-of-concept D–A-PS-CAT dyad, and many design alterations are possible to improve performance in the future.

Control experiments with no catalyst and no LED irradiation demonstrated that all components are crucial to form NADH (Fig. 6e). Prior to catalysis, BIH-OMe was also tested in emission quenching experiments of the PS 2^*t*^BuCzDPPZ, which resulted in a bimolecular quenching constant of *k*_q-*I*_ = 5.06 × 10^9^ M^−1^ s^−1^ for emission quenching, and a bimolecular quenching constant of *k*_q-*τ*_ = 7.46 × 10^9^ M^−1^ s^−1^ for lifetime quenching (see SI Fig. S35). This is close to the diffusion-controlled limit of the quenching rate, which is ∼1 × 10^10^ M^−1^ s^−1^ (ref. 52) and confirms that electron transfer from BIH-OMe to the CT-excited state of the PS 2^*t*^BuCzDPPZ is a rapid event.

It is worth noting that BIH-OMe already performs well at a relatively low quantity of 80 equivalents of the PS-CAT dyad. When increasing the concentration from 250 µM to 400 µM, significantly more NADH is produced over time. However, beyond this point (3300 µM of BIH-OMe), no further NADH is formed, but the catalyst starts degrading (see SI Fig. S47). In comparison to photocatalysis with other dyads using 12 mM TEA as an SED,^10^ in our case 300-fold lower SED concentration is needed to run the reaction, meaning that less chemical waste is produced.

On performing the light-driven catalysis at a higher temperature, emission bands in the range from 370 to 385 nm can be observed, which can be attributed to a degradation product of BIH-OMe (SI, Fig. S46). These bands increase in intensity with the irradiation time. This indicates that BIH-OMe undergoes some kind of side reaction, which is not coupled with the actual catalysis. This side reaction becomes dominant at higher temperatures making BIH-OMe not suitable as an SED under these conditions.

## Conclusions

We herein present the donor–acceptor design of the photosensitizer-catalyst (PS-CAT) dyad 2^*t*^BuCzDPPZRhCp* for nicotinamide hydrogenation/reduction. PS-CAT dyads mimic part of nature's complex assembly active in photosynthesis. The organic PS part with a donor (carbazole)-acceptor (dipyrido[3,2-*a*:2,3-*c*]phenazine = DPPZ) structure and substantial visible-light absorption contains a bidentate coordination site for a catalytically active rhodium(iii) center. The presented derivatives of this principal building block all show photoluminescence with lifetimes of 10 ns and 36% photoluminescence quantum yield. Photophysical investigations on the PS 2^*t*^BuCzDPPZ, including femtosecond transient absorption (fs-TA) measurements and supported by TDDFT calculations, revealed a strong solvent (polarity) effect on photoluminescence quantum yields and on the excited-state properties. In addition, we postulate that a fast excited-state energy transfer from the 2^*t*^BuCzDPPZ PS to the Rh center takes place. This can be considered as a deactivation pathway of the crucially important excited state and might be a reason for the low photocatalytic activity. The PS-CAT dyad was employed in the light-driven NAD^+^ to NADH reduction and showed – after identification of BIH-OMe as a suitable sacrificial electron donor (SED) – photocatalytic performance with a TON of 3.2 (after 4 h) and a TOF of up to 1.2 h^−1^. The fact that only a strong SED resulted in catalytic turnover is an indication that the lifetime of the charge-separated state is short and therefore limits the overall photocatalytic potential. This study provides a new approach to PS-CAT dyads using organic D–A chromophores as a PS, stimulating the future development of new types of organic PS-based dyads. A future pathway for the optimization opened by this combined synthetic, theoretic and spectroscopic investigation is to close the energy transfer pathway between the charge-separated state and the Rh(iii)Cp* center, to favor the required electron transfer pathway.

## Author contributions

Conceptualization: B. E., A. T., and B. D.-I.; data curation: all authors; formal analysis: A. T., M. S., and T. M.; funding acquisition: B. E., B. D.-I., S. K., S. R., and A. J. C. K.; investigation: A. T., M. S., T. M., and D. S.; methodology: all authors; project administration: B. E., B. D.-I., and S. K.; resources: B. E., B. D.-I., S. K., S. R., and A. J. C. K.; supervision: B. E., B. D.-I., and S. K.; validation: all authors; visualization: A. T., M. S., T. M., and B. E.; writing – original draft: A. T. and B. E.; writing – review & editing: all authors.

## Conflicts of interest

There are no conflicts to declare.

## Supplementary Material

SC-OLF-D5SC08675B-s001

## Data Availability

The data obtained in this study have been deposited in the repository Zenodo and are available under the digital object identifier: https://doi.org/10.5281/zenodo.17493527. Supplementary information (SI) is available. See DOI: https://doi.org/10.1039/d5sc08675b.
