# Supplementary material for: A donor–acceptor photosensitizer-catalyst dyad for light-driven nicotinamide hydrogenation
Source: Chem Sci. 2025 Dec 23;17(6):3012–22. doi: 10.1039/d5sc08675b (PMC12850510; doi:10.1039/d5sc08675b)
Supplement: SC-017-D5SC08675B-s001 [file SC-017-D5SC08675B-s001.pdf]

Supplementary Information (SI) for Chemical Science.
This journal is © The Royal Society of Chemistry 2025

A Donor-Acceptor Photosensitizer-catalyst Dyad for Light-Driven Nicotinamide Hydrogenation

Alexander Tombrink,[a]+ Mohini Semwal,[b,c]+ Tamar Maisuradze,[c]+ Alexander K. Mengele,[d] Daniel Straub,[e] Alexander J.C. Kuehne,[e] Sven Rau,[d] Stephan Kupfer,[c] Benjamin Dietzek-Ivanšić,*[b,c,f] and Birgit Esser*[a]

[a] Institute of Organic Chemistry II and Advanced Materials, Ulm University, Albert-Einstein-Allee 11, 89081 Ulm, Germany

[b] Leibniz Institute of Photonic Technology, Research Department Functional Interfaces, Albert-Einstein-Str. 9, 07745 Jena, Germany

[c] Institute of Physical Chemistry, Friedrich Schiller University Jena, Helmholtzweg 4, 07743 Jena, Germany

[d] Institute of Inorganic Chemistry I, Ulm University, Albert-Einstein-Allee 11, 89081 Ulm, Germany

[e] Institute of Organic Chemistry III, Ulm University, Albert-Einstein-Allee 11, 89081 Ulm, Germany

[f] Leibniz-Institut für Oberflächenmodifizierung e.V. (IOM), Permoserstraße 15, 04318 Leipzig, Germany

+ These authors contributed equally

Table of Contents

1. Materials and Methods 3
   1.1. Chemicals and Solvents 3
   1.2. Flash Column Chromatography 3
   1.3. Thin-Layer Chromatography 3
   1.4. Nuclear Magnetic Resonance (NMR) Spectroscopy 3
   1.5. High-Resolution Mass Spectroscopy (HRMS) 4
   1.6. UV/Vis Absorption Spectra 4
   1.7. Fluorescence Spectra 4
   1.8. Phosphorescence Spectra 4
   1.9. Photoluminescence Quantum Yields 4
   1.10. Time-correlated Single Photon Counting 4
   1.11. Cyclic Voltammetry 4
   1.12. Transient Absorption Spectroscopy 5
   1.13. Resonance Raman Spectroscopy 5
   1.14. Computational Details 6
2. Synthetic Procedures 9
3. NMR Spectra 19
4. Cyclic Voltammograms 31
5. Photophysical Measurements 32
   5.1. Transient Absorption Spectroscopy 32
   5.2. Determination of ΔE_{ST} 35
   5.3. Stern-Volmer Quenching Experiments 37
   5.4. Emission Decay 38
6. Catalysis 40
   6.1. Determination of NADH Formation 40
   6.2. Formate-driven Catalysis 42
   6.3. Light-driven Catalysis 43
7. Computational Results 47
8. Additional Figure 68
9. Literature 69

1. Materials and Methods

1.1. Chemicals and Solvents

Chemicals were purchased from ABCR, Acros-Organics, Alfa-Aesar, Bernd-Kraft, ChemPur, Roth, Sigma-Aldrich, BLD Pharm or TCI and used directly without further purification unless otherwise noted. 2-(4-Methoxyphenyl)-1,3-dimethyl-2,3-dihydro-1*H*-benzo[*d*]imidazole (BIH-OMe) was synthesized according to a published procedure.[1] Moisture- or oxygen-sensitive reactions were carried out in dried glassware, heated under vacuum (10^{-3} mbar) using standard Schlenk techniques in dry argon atmosphere (Argon 5.0 from SAUERSTOFFWERKE FRIEDRICHSHAFEN). Anhydrous solvents (THF, toluene, CH_2Cl_2) were obtained from an M. BRAUN solvent purification system (MB-SPS-800) and stored over 3 Å molecular sieves.

1.2. Flash Column Chromatography

Column chromatography was carried out using silica gel 60, grain size 40–63 µm (230–400 mesh) from MACHERY-NAGEL.

1.3. Thin-Layer Chromatography

Analytical thin-layer chromatography was carried out using silica gel-coated aluminum plates with a fluorescence indicator (Merck 60 F_{254}). Detection was carried out using short-wave UV-light (λ_{max} = 254 nm; 350 nm).

1.4. Nuclear Magnetic Resonance (NMR) Spectroscopy

NMR spectra were recorded at 298 K on the following spectrometers: BRUKER *Avance Neo 400* [400.1 MHz (^{1}H), 101.6 MHz (^{13}C)], and BRUKER *Avance Neo 600* [600.2 MHz (^{1}H), 150.9 MHz (^{13}C)]. Structural assignments were made with additional information from gCOSY, gHSQC, and gHMBC experiments. Chemical shifts are reported in parts per million (ppm, δ scale) relative to the signal of the residual solvent signal of the respective solvent: $CDCl_3$: δ = 7.26 ppm; CD_2Cl_2 = 5.32 ppm; ^{13}C NMR spectra are referenced to the following signals: $CDCl_3$: δ = 77.16 ppm; CD_2Cl_2 = 53.84 ppm. Analysis followed first order, and the following abbreviations for multiplets were used: singlet (s), broad singlet (br. s), doublet (d), triplet (t), quartet (q), septet (sept), multiplet (m) and combinations thereof, i.e. doublet of doublets (dd). Coupling constants (*J*) are given in Hertz / Hz. Solvent signals were not integrated, and the most common solvent shifts for the ^{1}H NMR spectra in $CDCl_3$ are: dichloromethane (5.30 ppm), acetone (2.17 ppm), H_2O (1.56 ppm), and silicon grease (0.07 ppm). The chemical shifts of the most common solvents in ^{13}C NMR spectra in $CDCl_3$ are: dichloromethane (53.3 ppm),

acetone (207.1, 30.9 ppm), and silicon grease (1.2 ppm). Signal orientations in DEPT experiments were described as follows: o = no signal; + = up (CH, CH_3); - = down (CH_2).

1.5. High-Resolution Mass Spectroscopy (HRMS)

High-resolution mass spectrometry (HRMS) was performed using a Fourier Transform Ion Cyclotron Resonance (FT-ICR) mass spectrometer solariX (Bruker Daltonik) equipped with a 7.0 T superconducting magnet and interfaced to an Apollo II Dual ESI/MALDI source.

1.6. UV/Vis Absorption Spectra

Ground-state UV/Vis absorption spectroscopy was performed on a V-670 JASCO UV-VIS-NIR Spectrometer.

1.7. Fluorescence Spectra

Emission spectroscopy was performed on a JASCO FP-8500 spectrofluorometer.

1.8. Phosphorescence Spectra

Phosphorescence spectra were measured with a Perkin Elmer FL 6500.

1.9. Photoluminescence Quantum Yields

The PLQY were determined with a Hamamatsu Quantaurus-QY (C11347).

1.10. Time-correlated Single Photon Counting

Fluorescence lifetimes were recorded with a DeltaPro from Horiba Scientific using a 481, 451 or 372 nm pulsed Laser source (Class 3B Laser Product, <0.5 W peak in pulsed and CW mode). The Delta Pro consists of: DeltaDiode (Picosecond diode controller), DeltaHub (High throughput TCSPC controller), DPS-1 (Detector Power supply) and a PPD (Picosecond photon detection module). The IRF (Instrument response function) was measured with LUDOX silica nanoparticles. Fits were done with the software DecayFit – Emission Decay Analysis Software (Version 1.4).

1.11. Cyclic Voltammetry

Cyclic voltammograms were measured in anh. degassed DCM solution in an argon-filled glovebox using a METROHM Autolab PGSTAT128N. As the working electrode, a glassy carbon disc electrode (2 mm in diameter) was used, and a platinum rod served as the counter electrode. As the reference electrode, an Ag/AgNO3 electrode containing a silver wire immersed in an inner chamber filled with 0.1 M AgNO3 and an

outer chamber containing 0.1 M n-Bu_4NPF_6 were used. For internal reference, the ferrocene/ferrocenium (Fc/Fc+) redox couple was used.

1.12. Transient Absorption Spectroscopy

Femtosecond transient absorption (fs-TA) experiments were conducted on a home-built setup described in detail elsewhere.[2,3] The system was powered by a Ti:sapphire regenerative amplifier (Astrella, Coherent, USA) that delivers 800 nm pulses at 1 kHz repetition rate. White-light probe continua covering 300–700 nm were produced by focusing part of the amplifier output into a rotating CaF_2 crystal. This probe light was split into reference and measurement channels. Pump pulses were generated from the remaining 800 nm output using an optical parametric amplifier (TOPAS Prime, Light Conversion, Lithuania). Pump and probe beams were combined at the sample position with magic-angle polarization (54.7°). Excitation was carried out at 400 and 470 nm.

Samples were contained in 1 mm quartz cuvettes and adjusted to an optical density of 0.1–0.3 at the excitation wavelength. Pump pulses were set to 0.5 μJ per pulse. To exclude artifacts from sample degradation, UV/Vis absorption spectra were collected before and after each measurement.

Transient absorption data were processed using KiMoPack package.[4] The chirp was corrected prior to global fitting, which was performed with a sequential kinetic scheme and a 95% confidence criterion. To minimize coherent artifact contributions, the first 300 fs around time-zero were omitted from the kinetic analysis.[5]

1.13. Resonance Raman Spectroscopy

Resonance Raman (rR) spectra were acquired using two excitation sources: a single-longitudinal-mode diode laser at 405 nm (TopMode-405-HP, Toptica, Germany) and a 473 nm diode-pumped solid-state laser (HB-Laser, Germany). Raman scattering was collected with an IsoPlane 160 spectrometer (Princeton Instruments, USA) equipped with gratings of 2400, 1200, and 600 grooves/mm. A 30 μm entrance slit provided high spectral resolution. To suppress Rayleigh scattering, long-pass filters matched to the excitation wavelengths (Semrock, USA) were applied. The laser power at the sample was fixed at 8 mW to minimize local heating and photodegradation. Acetonitrile was used as an internal reference, and its Raman band at 1370 cm^{-1} was employed for both spectral calibration and relative intensity normalization.

1.14. Computational Details

PS: 2^{t}BuCzDPPZ

All quantum chemical calculations were carried out using the ORCA 6.0 program.[6,7] The singlet (S_0) and triplet (T_1) ground state structures of 2^{t}BuCzDPPZ were obtained by density functional theory (DFT) by applying the PBE0 functional[8,9] and in combination with the def2-tzvpp basis set.[10,11] The frequency analysis (analytical) was carried out to verify that all obtained ground state geometries are (local) minimum on the potential energy surface (PES).

Scalar relativistic time-dependent DFT (TDDFT) within the Tamm-Dancoff approximation (TDA) was applied to obtain 50 lowest excited singlet-singlet, singlet-triplet and/or triplet-triplet states each, along with spin-orbit couplings (SOCs) in S_0 and T_1 geometries. In contrast to TDDFT, TDA neglects deexcitations from the Casida equation, which is often beneficial for the energetic description of triplet excited states.[6,12,13] Furthermore, the relaxed geometry of S_1 excited state was obtained using this computational setup. Relativistic effects were accounted for using second-order Douglas-Kroll-Hess method and relativistic triple-zeta old-dkh-tzvpp basis set introduced in previous versions of ORCA program. Second-order picture-change correction with point nucleus method was applied. The transient absorption spectra were modelled by assuming 1:1 population of S_0 and T_1, with triplet-triplet excitations from T_1 representing excited state absorption and singlet-singlet transitions from S_0 representing the ground state bleach. Such approach allows to model the transient absorption signal of specific exited states upon excitation of the longest wavelength absorption band at long delay times.[14–18]

The solute electron density (SMD) variant of the conductor-like polarizable continuum model (CPCM) model was applied to account for the solvent effects of acetonitrile and toluene.

To reduce the computational cost of the calculations, the resolution of identity approximation for Coulomb integrals and numerical integration of HF exchange (RIJCOSX) approximation[19–21] with def2/J auxiliary basis set[10] were used for all calculations.

Downhill intersystem crossing (ISC) rates for calculated using Fourier transformed simplified rate constant formula with Lorentzian dephasing[22]:

$$k_{ISC,down}=\frac{2\gamma}{\hbar(E_{kl}^2+\gamma^2)}|SOC|^2 \qquad eq.\ 1,$$

where γ represents half width at half maximum (0.2 eV used in all cases), $\hbar$ is the reduced Plank constant, E_{kl} and SOC are adiabatic energy difference and the spin-orbit coupling between two states ($SOC=\langle T_l|\hat{H}_{SOC}|S_k\rangle$).

The rate of fluorescence was calculated according to the Einstein coefficient *A* for spontaneous emission[23]:

$$A=k_F=\frac{\omega_F^3}{3\epsilon_0\pi\hbar^4c^3}\mu_F^2 \qquad eq.\ 2,$$

where, ω_F denotes the resonance frequency of $S_1 \rightarrow S_0$ the transition, μ_F is the respective transition dipole moment, *c* is the speed of light in vacuum and ϵ_0 is vacuum permittivity.

All optimized structures (S_0,T_1) obtained at PBE0 level of theory, along with extended SOC data and high-resolution images of spin densities and charge density differences are available from the online repository Zenodo.

PS-CAT: 2^{t}BuCzDPPZRhCp*

Once more, all quantum chemical calculations were carried out using the ORCA 6.0 program[6,7]. The Frank-Condon equilibrium (singlet ground state, S_0) structure of 2^{t}BuCzDPPZRhCp* was obtained at density functional level of theory (DFT) by applying the range-separated ωB97x-d3 functional as well as by means of the PBE0 hybrid functional.[8,9] In both cases, the def2-tzvpp basis set and the corresponding pseudo potential for Rh was utilized.[10,11] The frequency analysis (analytical) was carried out to verify the presence of a (local) minimum on potential energy surface (PES).

In a similar fashion as outlined above for the PS, scalar relativistic TDDFT calculations were performed within and without (TDA). In each scenario, the 50 lowest energy excited singlet-singlet and singlet-triplet states were computed – along with SOCs. Relativistic effects as well as implicit solvent effects (toluene) were accounted for in an analogous fashion as described above for 2^{t}BuCzDPPZ, while the resolution of identity approximation was utilized (RIJCOSX; def2/J auxiliary basis).

Unfortunately, neither the applied range-separated nor the hybrid functional provide a fully satisfying description of the electronic transitions in 2^{t}BuCzDPPZRhCp*. However, and in line with our previous computational studies on 4d and 5d-based transition metal complexes which include an additional organic chromophore hybrid functionals with a slightly extended ratio of exact exchange provide a more reliable description of the electronic transitions with respect to experimental and multiconfigurational reference data.[24–28]

2. Synthetic Procedures

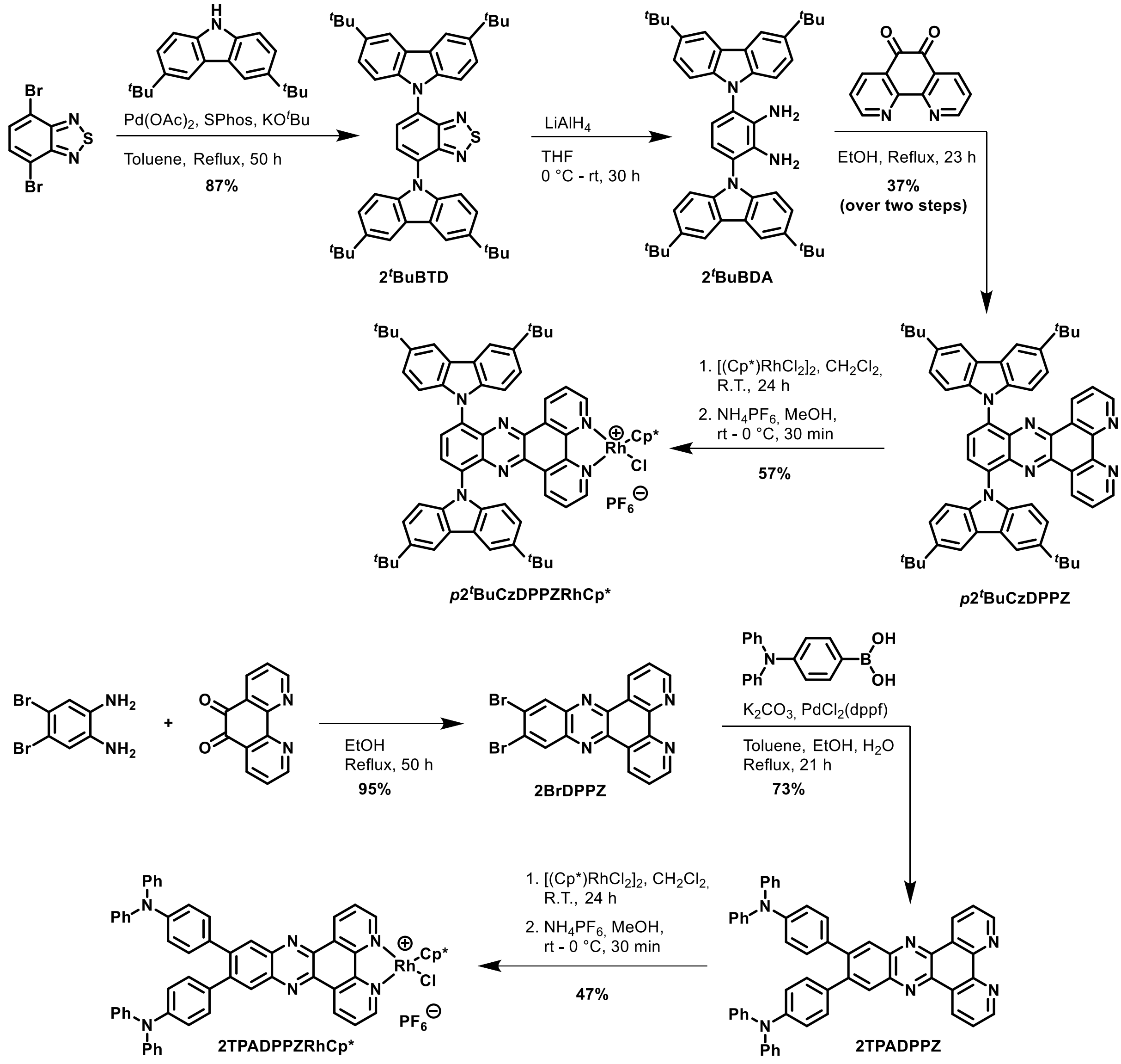

Scheme S1: Synthesis of the D-A-ligands *p*2^{t}BuCzDPPZ and 2TPADPPZ, and their Rh complexes *p*2^{t}BuCzDPPZRhCp* and 2TPADPPZRhCp*.

11,12-Difluorodipyrido[3,2-a:2',3'-c]phenazine (2FDPPZ)

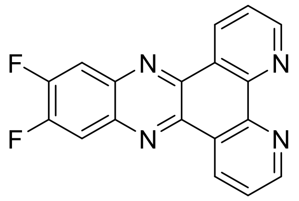

This compound was prepared according to a modified procedure published by GARCÍA-LÓPEZ *et al*. The NMR spectra are consistent with the literature data.[29]

1,10-Phenanthroline-5,6-dione (0.70 g, 3.33 mmol, 0.8 eq.) and 2-amino-4,5-difluoroaniline (0.60 g, 4.16 mmol, 1.0 eq.) were dissolved in 45 mL degassed absolute ethanol. The solution was refluxed at 90 °C under an argon atmosphere for 2.5 h. After removing the solvent under reduced pressure, the resulting brown crystals were filtered off and washed with methanol.

Yield: 0.79 g (86%) of brown needles.

***R*f**: 0.00–0.18 (methanol).

^{1}H NMR (400 MHz, $CDCl_3$): δ = 9.57 (d, *J* = 8.1 Hz, 2H), 9.38–9.19 (m, 2H), 8.08 (dd, *J* = 9.2, 9.2 Hz, 2H), 7.80 (dd, *J* = 8.2, 4.4 Hz, 2H).

^{13}C NMR (100 MHz, $CDCl_3$): δ = 154.30 (dd, *J* = 261.2, 18.8 Hz), 153.17, 148.63, 141.36, 140.10 (t, *J* = 6.0 Hz), 133.92, 127.26, 124.50, 114.91 (dd, *J* = 12.5, 6.5 Hz) ppm.

HRMS (pos. MALDI): *m/z* calcd. for $C_{18}H_8N_4F_2$ 318.0717 $[M+H]^+$, found 319.0789.

11,12-Bis(3,6-di-*tert*-butyl-9*H*-carbazol-9-yl)dipyrido[3,2-*a*:2',3'-*c*]phenazine (2^tBuCzDPPZ)

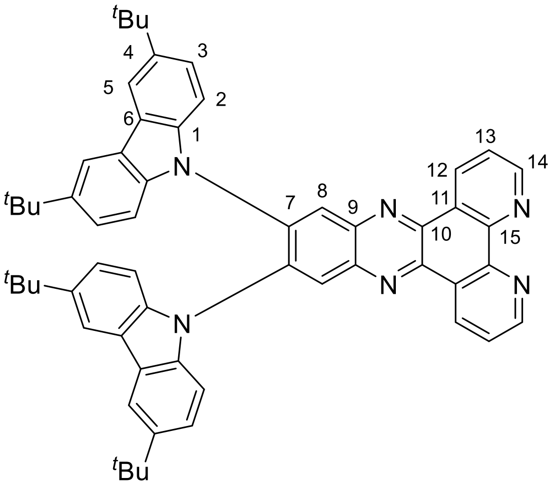

The synthesis was carried out under an argon atmosphere. A 50 mL, flame-dried, two-necked round-bottomed flask with condenser was charged with 3,6-di-*tert*-butyl-9*H*-carbazole (1.95 g, 6.91 mmol, 2.2 eq.). After adding 25 mL dry THF, the solution was stirred for 10 min at 0 °C. NaHMDS (1 M solution in THF; 7.81 mL, 7.81 mmol, 2.49 eq.) was added to the suspension, which was then stirred for 30 min at room temperature. **2FDPPZ** (1.00 g, 3.14 mmol, 1.0 eq.) was suspended in 29.3 mL dry THF, and the suspension was transferred in the reaction vessel. The reaction mixture was stirred for 41 h at reflux temperature. After removing the solvent, the crude product was purified by filtration through a silica plug (silica gel, ethyl acetate). Washing with MeCN/acetone 10/1 (v/v) gave the desired product.

Yield: 1.77 g (67%) of an orange solid.

$\boldsymbol{R}_f$: 0.00–0.09 (methanol).

^{1}H NMR (600 MHz, $CDCl_3$): δ = 9.65 (dd, *J* = 8.0, 1.8 Hz, 2H, 12-H), 9.31 (dd, *J* = 4.5, 1.8 Hz, 2H, 14-H), 8.82 (s, 2H, 8-H), 7.81 (dd, *J* = 8.1, 4.3 Hz, 2H, 13-H), 7.63 (d, *J* = 1.9 Hz, 4H, 5-H), 6.96 (d, *J* = 8.6 Hz, 4H, 2-H), 6.91 (dd, *J* = 8.6, 1.9 Hz, 4H, 3-H), 1.35 (s, 36H, 4-C(CH_3)$_3$).

^{13}C NMR (150 MHz, $CDCl_3$) δ = 153.00 (+, C14), 148.69 (o, C15), 143.42 (o, C4), 142.02 (o, C9), 141.82 (o, C7), 138.19 (o, C1), 137.5 (o, C10), 134.07 (+, C12), 130.05 (+, C8), 127.56 (o, C11), 124.47 (+, C13), 124.00 (o, C6), 123.03 (+, C3), 115.57 (+, C5), 108.98 (+, C2), 34.64 (o, C4-*C*(CH_3)$_3$), 32.01 (+, C4-C(*C*H_3)$_3$).

HRMS (pos. MALDI): *m/z* calcd. for $C_{58}H_{56}N_6$ 836.4566 $[M]^{\bullet+}$, found 836.4563.

11,12-Bis(3,6-di-*tert*-butyl-9*H*-carbazol-9-yl)dipyrido[3,2-*a*:2',3'-*c*]phenazine-RhClCp*PF$_6$ (2^{t}BuCzDPPZRhCp*)

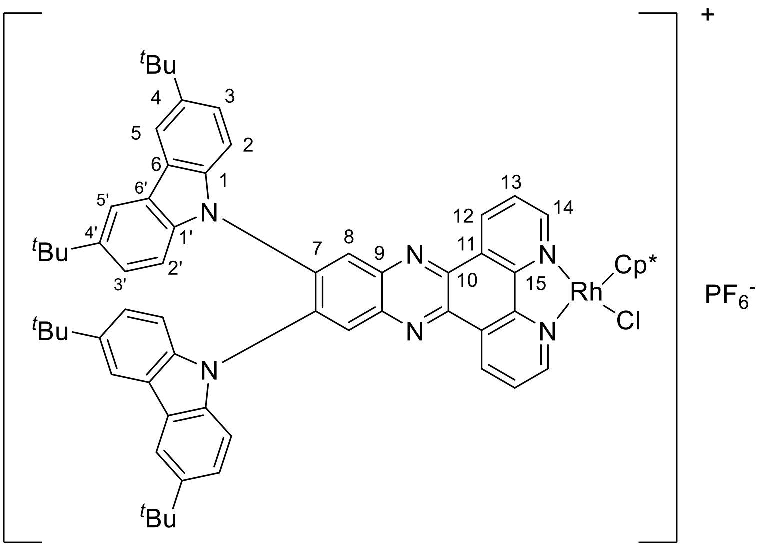

2^{t}BuCzDPPZ (51 mg, 0.06 mmol, 1 eq.) and pentamethylcyclopentadienylrhodium(III) chloride dimer (19 mg, 0.03 mmol, 0.5 eq.) were dissolved in 10 mL dichloromethane. The solution was stirred for 24 h at room temperature. After removing the solvent under reduced pressure, the crude product was purified by size-exclusion chromatography (Sephadex, methanol/acetone/chloroform: 47/30/23). In a second step, the salt was dissolved in methanol, and 1 mL of an aqueous solution of NH_4PF_6 (20 mg, 0.12 mmol, 2.0 eq.) was added to obtain the desired product.

Yield: 0.03 g (44%) of a red solid.

^{1}H NMR (600 MHz, $CDCl_3$): δ = 9.81 (dd, *J* = 8.1, 1.4 Hz, 2H, 12-H), 9.23 (dd, *J* = 5.2, 1.4 Hz, 2H, 14-H), 8.91 (s, 2H, 8-H), 8.17 (dd, *J* = 8.1, 5.2 Hz, 2H, 13-H), 7.62 (d, *J* = 1.9 Hz, 2H, 5-H), 7.60 (d, *J* = 1.9 Hz, 2H, 5'-H), 7.01–6.84 (m, 8H, 2-H, 3-H, 2'-H, 3'-H), 1.83 (s, 15H, Cp*-CH_3), 1.35 (s, 18H, 4-C(CH_3)$_3$), 1.31 (s, 18H, 4'-C(CH_3)$_3$).

^{13}C NMR (150 MHz, $CDCl_3$): δ = 152.63 (+, C14), 148.26 (o), 143.79 (o, C4), 143.72 (o, C4'), 142.26 (o), 140.03 (o), 138.89 (o), 137.95 (o), 137.88 (o), 136.52 (+, C12), 130.58 (o), 130.08 (+, C8), 128.08 (+, C13), 124.12 (o), 124.09 (o), 123.24 (+), 123.00 (+), 115.67 (+, C5), 115.64 (+, C5'), 109.01 (+), 108.86 (+), 97.83 (o, Cp*), 34.68 (o,

C4-*C*(CH_3)$_3$), 34.62 (o, C4'-*C*(CH_3)$_3$), 32.01 (+, C4-C(*C*H_3)$_3$), 31.95 (+, C4'-C(*C*H_3)$_3$), 9.13 (+, Cp*-*C*H_3).*

HRMS (pos. MALDI): *m/z* calcd. for $C_{68}H_{71}N_6ClRh$ 1109.4489 $[M{-}PF_6]^+$, found 1109.4475.

*Due to the limited resolution of the 2D NMR spectra it was not possible to assign every peak to a specific carbon atom.

4,7-Bis(3,6-di-*tert*-butyl-9*H*-carbazol-9-yl)benzo[*c*][1,2,5]thiadiazole (**2*t*BuCzBTD**)

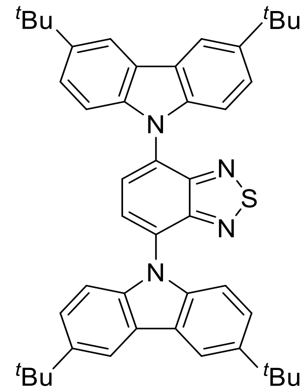

This compound was prepared according to a modified procedure published by GUDEIKA *et al*. The NMR spectra are consistent with the literature data.[30]

4,7-Dibromobenzo[*c*][1,2,5]thiadiazole (200 mg, 0.68 mmol, 1.0 eq.), 3,6-di-*tert*-butyl-9*H*-carbazole (532 mg, 1.90 mmol, 2.8 eq.), KO*t*Bu (183 mg, 1.63 mmol, 2.4 eq.), $Pd(OAc)_2$ (45 mg, 0.20 mmol, 0.3 eq.) and SPhos (84 mg, 0.20 mmol, 0.3 eq.) were dissolved in anh. and degassed toluene (24 mL) and stirred at reflux temperature for 50 h under an argon atmosphere. After removing of the solvent, the crude product was washed with acetone to give the desired product.

Yield: 0.37 g (78%) of a yellow solid.

***R*f**: 0.80 (petroleum ether:ethyl acetate 40:1).

^{1}H NMR (400 MHz, CD_2Cl_2): δ = 8.24 (d, *J* = 1.9 Hz, 4H), 8.05 (s, 2H), 7.51 (dd, *J* = 8.7, 1.9 Hz, 4H), 7.26 (d, *J* = 8.7 Hz, 4H), 1.50 (s, 36H).

^{13}C NMR (100 MHz, CD_2Cl_2) δ = 153.11, 144.09, 139.96, 130.07, 128.05, 124.24, 124.12, 116.91, 110.35, 35.11, 32.14.

HRMS (pos. MALDI): *m/z* calcd. for $C_{46}H_{50}N_4S$ 690.3756 $[M]^{\bullet+}$, found 690.3755.

3,6-Bis(3,6-di-*tert*-butyl-9*H*-carbazol-9-yl)benzene-1,2-diamine (2*t*BuBDA)

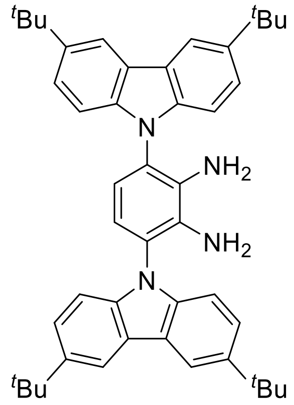

The reaction was carried out in an oven-dry Schlenk tube under an argon atmosphere. 2*t*BuBTD (150 mg, 0.22 mmol, 1.0 eq.) was dissolved in 12 mL dry and degassed THF. The mixture was cooled to 0 °C and $LiAlH_4$ (124 mg, 3.26 mmol, 15 eq.) was added stepwise. After 30 h the reaction was quenched with water (10 mL) and extracted with diethyl ether (3 x 20 mL). The organic layers were collected and were dried by the addition of $MgSO_4$. The mixture was filtered through a glass funnel and the solvent was evaporated under reduced pressure. The crude product was used immediately without further purification for the following reaction.

10,13-Bis(3,6-di-*tert*-butyl-9*H*-carbazol-9-yl)dipyrido[3,2-*a*:2',3'-*c*]phenazine (*p*2*t*BuCzDPPZ)

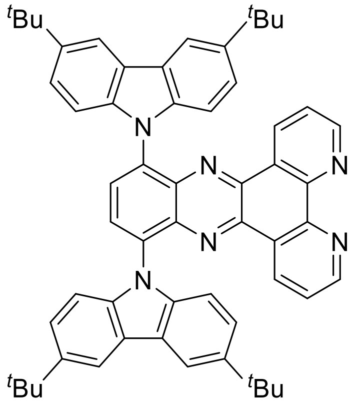

2*t*BuBDA (144 mg, 0.22 mmol, 1eq.) and 1,10-phenanthroline-5,6-dione (228 mg, 1.09 mmol, 5 eq.) were dissolved in 20 mL degassed absolute ethanol. The solution was refluxed at 100 °C under an argon atmosphere for 23 h. After removing of the

solvent, the crude product was purified by column chromatography (silica gel, petroleum ether:ethyl acetate 1:3).

Yield: 67 mg (37%) of a red solid.

***R*f**: 0.18–0.38 (petroleum ether:ethyl acetate 1:3).

^{1}H NMR (600 MHz, CD_2Cl_2): δ = 9.02 (dd, *J* = 4.5, 1.8 Hz, 2H), 8.43 (s, 2H), 8.39 (dd, *J* = 8.1, 1.8 Hz, 2H), 8.34 (d, *J* = 2.0 Hz, 4H), 7.46 (dd, *J* = 8.7, 1.9 Hz, 4H), 7.37 (dd, *J* = 8.1, 4.5 Hz, 2H), 7.31 (d, *J* = 8.6 Hz, 4H), 1.52 (s, 36H).

^{13}C NMR (150 MHz, CD_2Cl_2) δ = 152.84, 148.87, 143.59, 141.10, 140.94, 139.40, 135.64, 133.83, 129.33, 127.21, 124.13, 123.96, 123.71, 116.36, 110.51, 34.86, 31.93.

HRMS (pos. MALDI): *m/z* calcd. for $C_{58}H_{56}N_6$ 836.4566 $[M]^{\bullet+}$, found 836.4559.

10,13-Bis(3,6-di-*tert*-butyl-9*H*-carbazol-9-yl)dipyrido[3,2-*a*:2',3'-*c*]phenazine $RhClCp^*PF_6$ (*p*2*ᵗ*BuCzDPPZRhCp*)

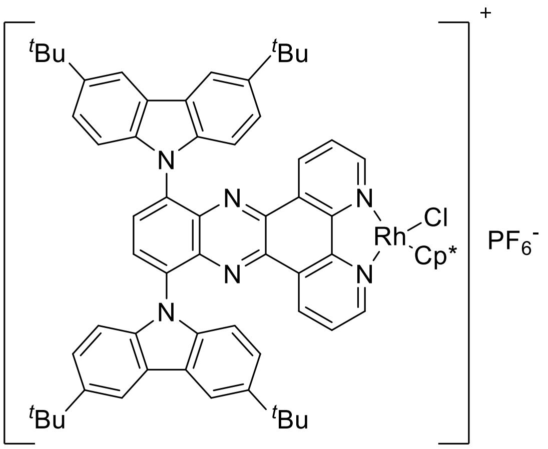

*p*2^{t}BuCzDPPZ (54 mg, 0.07 mmol, 1 eq.) and pentamethylcyclopentadienylrhodium(III) chloride dimer (20 mg, 0.03 mmol, 0.5 eq.) were dissolved in 10 mL dichloromethane. The solution was stirred for 24 h at room temperature. After removing of the solvent under reduced pressure, the crude product was purified by size-exclusion chromatography (spehadex, methanol/aceton/chloroform: 47/30/23). In a second step, the salt was dissolved in methanol and 1 mL of an aqueous solution of NH_4PF_6 (22 mg, 0.14 mmol, 2.0 eq.) was added to obtain the desired product.

Yield: 46 mg (57%) of a red solid.

^{1}**H NMR** (600 MHz, CD_2Cl_2): δ = 9.09 (d, *J* = 5.3 Hz, 2H), 8.63 (d, *J* = 8.2 Hz, 2H), 8.56 (s, 2H), 8.35 (d, *J* = 2.0 Hz, 4H), 7.85 (dd, *J* = 8.2, 5.3 Hz, 2H), 7.50 (dd, *J* = 8.2, 2.1 Hz, 2H), 7.45–7.40 (m, 2H), 7.37 (d, *J* = 8.6 Hz, 2H), 7.21 (d, *J* = 8.6 Hz, 2H), 1.75 (s, 15H), 1.53 (s, 18H), 1.52 (s, 18H).

^{13}**C NMR** (150 MHz, CD_2Cl_2) δ = 153.1, 148.4, 144.3, 144.2, 141.1, 140.9, 140.1, 139.1, 137.0, 136.1, 131.2, 130.5, 128.3, 124.4, 124.4, 124.2, 124.1, 116.8, 116.7, 110.9, 110.4, 98.0 (d, *J*(^{103}*Rh)* = 7.7 Hz), 35.2, 32.2, 9.3.

HRMS (pos. ESI): *m/z* calcd. for $C_{68}H_{71}N_6ClRh$ 1109.4489 $[M-PF_6]^+$, found 1109.4489.

11,12-Dibromodipyrido[3,2-*a*:2',3'-*c*]phenazine (2BrDPPZ)

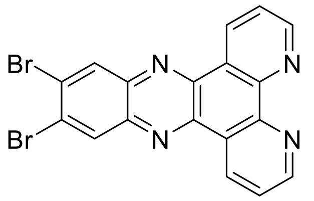

This compound was prepared according to a modified procedure by SCHÄFER *et al.*. The NMR spectra are consistent with the literature data.[31]

1,10-Phenanthroline-5,6-dione (1.00 g, 4.76 mmol, 1.0 eq.) and 4,5-dibromo-*o*-phenylendiamine (1.27 g, 4.76 mmol, 1.0 eq.) were heated in *abs*. ethanol (150 mL) at reflux temperature for 50 h. The organic solvent was removed under reduced pressure, and the crude product was washed with ethanol to give the desired product.

Yield: 1.98 g (95%) of a yellow solid.

^{1}**H NMR** (400 MHz, $CDCl_3$): δ = 9.55 (dd, *J* = 8.1, 1.7 Hz, 2H), 9.32 (dd, *J* = 4.6, 1.7 Hz, 2H), 8.67 (s, 2H), 7.83 (dd, *J* = 8.1, 4.4 Hz, 2H).

^{13}**C NMR** (100 MHz, $CDCl_3$) δ = 153.2, 148.5, 142.1, 141.5, 134.2, 133.5, 127.8, 127.3, 124.5.

4,4'-(Dipyrido[3,2-*a*:2',3'-*c*]phenazine-11,12-diyl)bis(*N*,*N*-diphenylaniline) (2TPADPPZ)

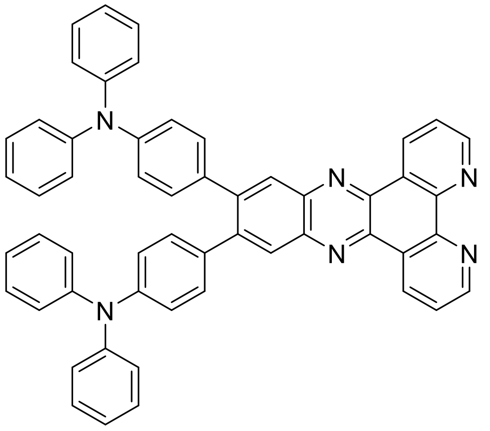

This compound was prepared according to a modified procedure by ADAMS *et al.*. The NMR spectra are consistent with the literature data.[32] The synthesis was carried out under an argon atmosphere in flame-dried glassware.

11,12-Dibromodipyrido[3,2-*a*:2',3'-*c*]phenazine (417 mg, 0.95 mmol, 1.0 eq.), 4-diphenylaminophenylboronic acid (1.17 g, 4.05 mmol, 4.3 eq.)), and potassium carbonate (1.30 g, 9.41 mmol, 9.9 eq.) were dissolved in a solvent mixture containing 20 mL toluene, 10 mL water and 5 mL ethanol. The mixture was purged with argon for 15 min. Then $PdCl_2$(dppf) (160 mg, 0.20 mmol, 0.21 eq.) was added. The reaction mixture was heated at reflux temperature for 21 h. After cooling, a saturated aqueous NH_4Cl solution was added to the reaction mixture followed by extraction with $CHCl_3$. The combined organic layers were dried with anhydrous $MgSO_4$. The organic solvent was removed under reduced pressure, and the crude product was washed with acetone to give the desired product.

Yield: 536 mg (73%) of a red solid.

^{1}H NMR (400 MHz, CD_2Cl_2): δ = 9.60 (dd, *J* = 8.1, 1.7 Hz, 2H), 9.20 (dd, *J* = 4.6, 1.7 Hz, 2H), 8.34 (s, 2H), 7.78 (dd, *J* = 8.1, 4.3 Hz, 2H), 7.33–7.19 (m, 12H), 7.13–6.98 (m, 16H).

^{13}C NMR (100 MHz, CD_2Cl_2) δ = 152.6, 148.8, 148.0, 147.7, 144.7, 142.3, 141.7, 134.5, 133.8, 131.3, 123.0, 129.7, 129.6, 128.1, 125.0, 124.5, 123.6, 123.1.

HRMS (pos. MALDI): *m/z* calcd. for $C_{54}H_{36}N_6$ 768.3001 $[M]^{\bullet+}$, found 768.2993.

4,4'-(Dipyrido[3,2-*a*:2',3'-*c*]phenazine-11,12-diyl)bis(*N*,*N*-diphenylaniline) RhClCp*PF$_6$ (2TPADPPZRhCp*)

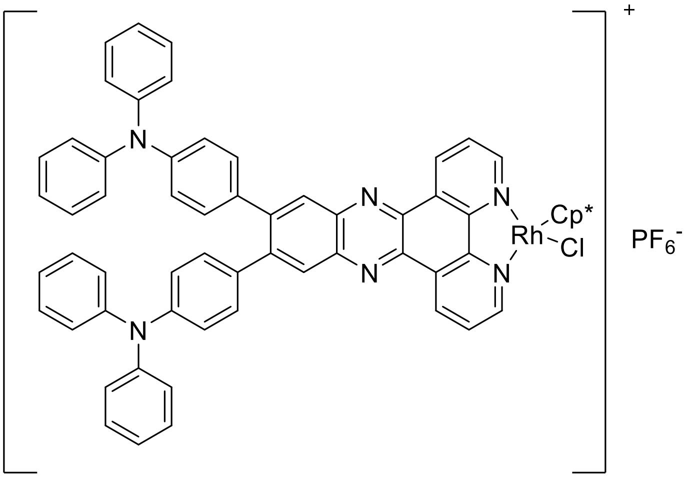

2TPADPPZ (103 mg, 0.13 mmol, 1.0 eq.) and pentamethylcyclopentadienylrhodium(III) chloride dimer (83 mg, 0.13 mmol, 1.0 eq.) were dissolved in 10 mL dichloromethane. The solution was stirred for 24 h at room temperature. After removing the solvent under reduced pressure, the crude product was purified by size-exclusion chromatography (spehadex methanol/acetone/chloroform: 47/30/23). In a second step, the salt was dissolved in methanol/acetone, and 1 mL of an aqueous solution of NH_4PF_6 (42 mg, 0.26 mmol, 2.0 eq.) was added to obtain the desired product.

Yield: 75 mg (47%) of a red solid.

^{1}H NMR (400 MHz, CD_2Cl_2): δ = 9.83 (d, J = 8.2 Hz, 2H), 9.24 (dd, *J* = 5.3, 1.4 Hz, 2H), 8.39 (s, 2H), 8.25 (dd, *J* = 8.2, 5.3 Hz, 2H), 7.34 – 7.18 (m, 12H), 7.17 – 7.00 (m, 16H), 1.84 (s, 15H).

^{13}C NMR (100 MHz, CD_2Cl_2) δ = 152.7, 148.1, 148.0, 147.8, 146.7, 142.9, 139.4, 136.7, 133.7, 131.2, 131.1, 130.0, 129.8, 128.4, 125.2, 123.8, 122.7, 97.9 (d, *J*(^{103}*Rh*) = 7.8 Hz), 9.4.

HRMS (pos. MALDI): *m/z* calcd. for $C_{64}H_{51}N_6ClRh$ 1041.2919 $[M{-}PF_6]^+$, found 1041.2915.

3. NMR Spectra

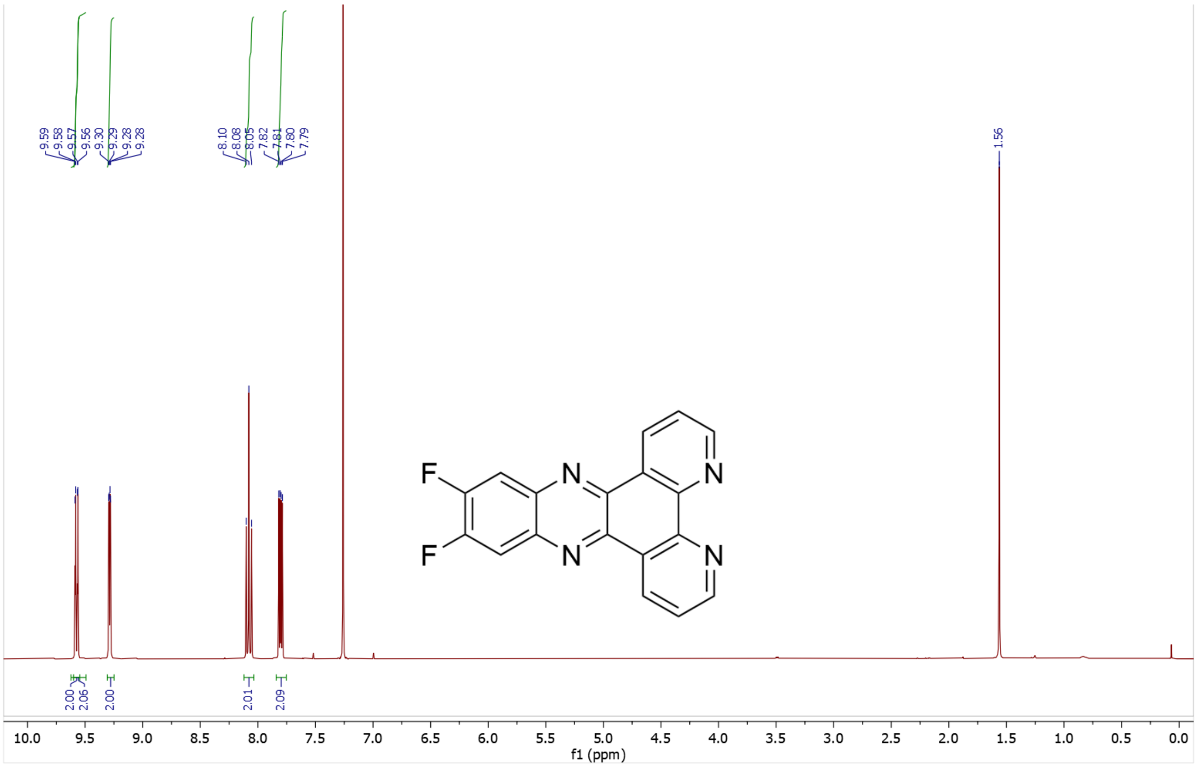

Figure S1: ^{1}H NMR spectrum of 2FDPPZ ($CDCl_3$, 400.1 MHz).

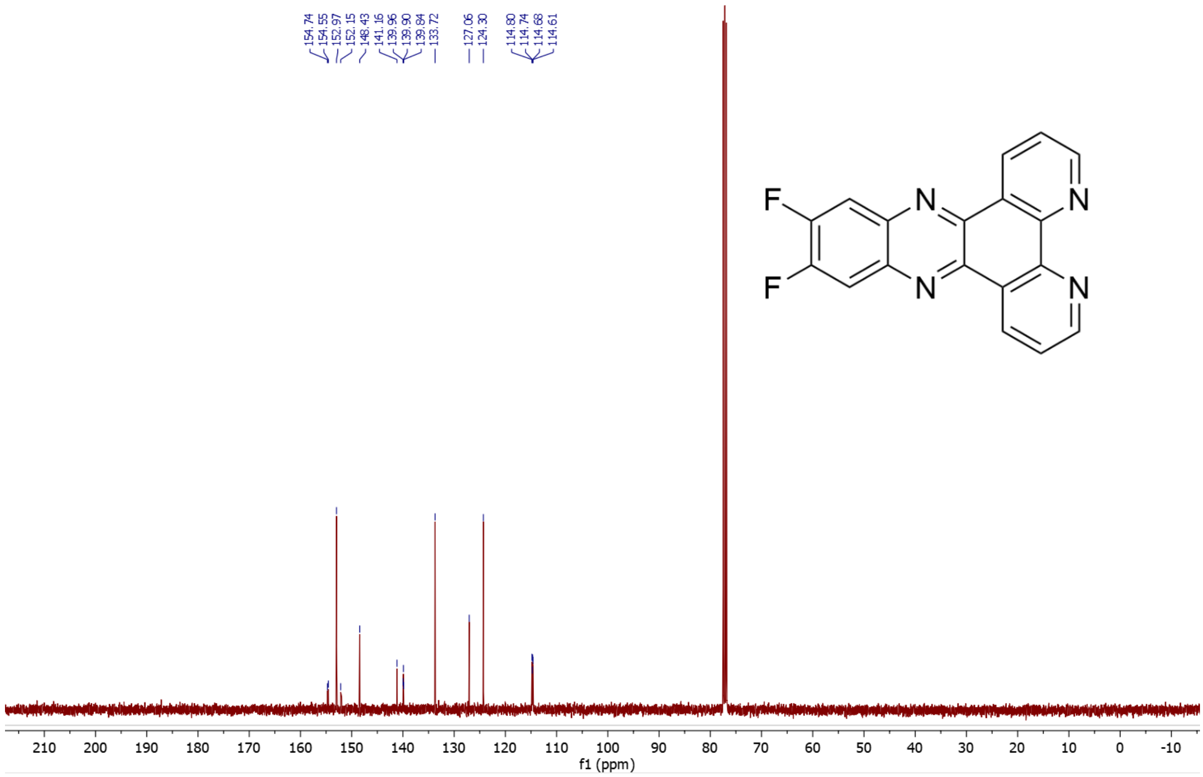

Figure S2: ^{13}C NMR spectrum of 2FDPPZ ($CDCl_3$, 101.6 MHz).

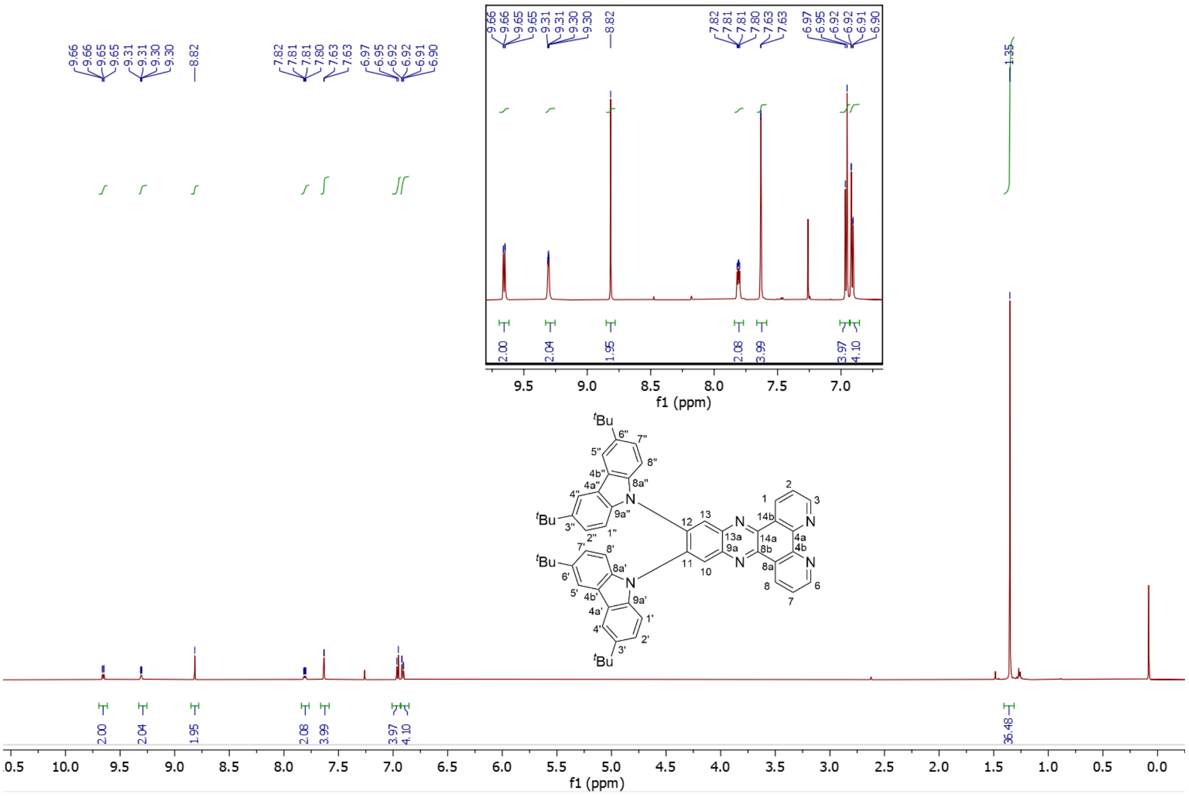

Figure S3: ^{1}H NMR spectrum of 2^{t}BuCzDPPZ ($CDCl_3$, 600.2 MHz).

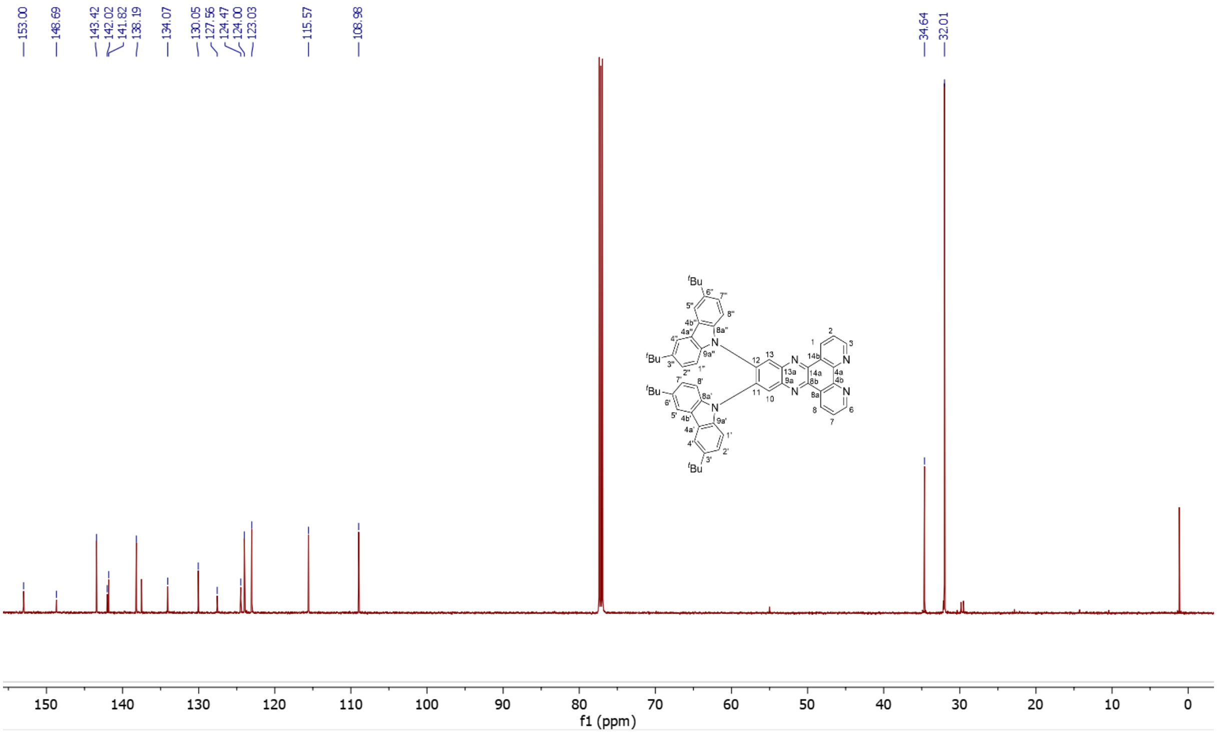

Figure S4: ^{13}C NMR spectrum of 2^{t}BuCzDPPZ ($CDCl_3$, 150.9 MHz).

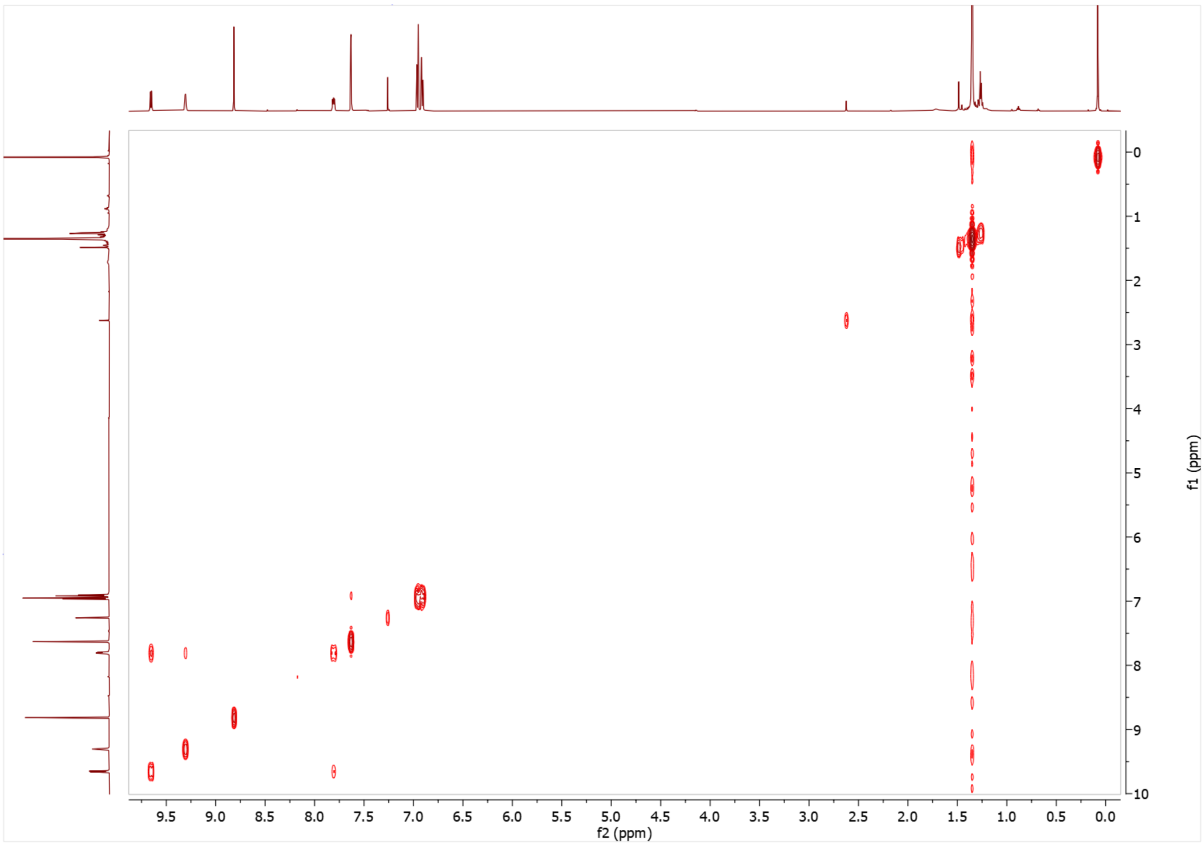

Figure S5: gCOSY 2D NMR spectrum of 2^{t}BuCzDPPZ ($CDCl_3$, 150.9 MHz).

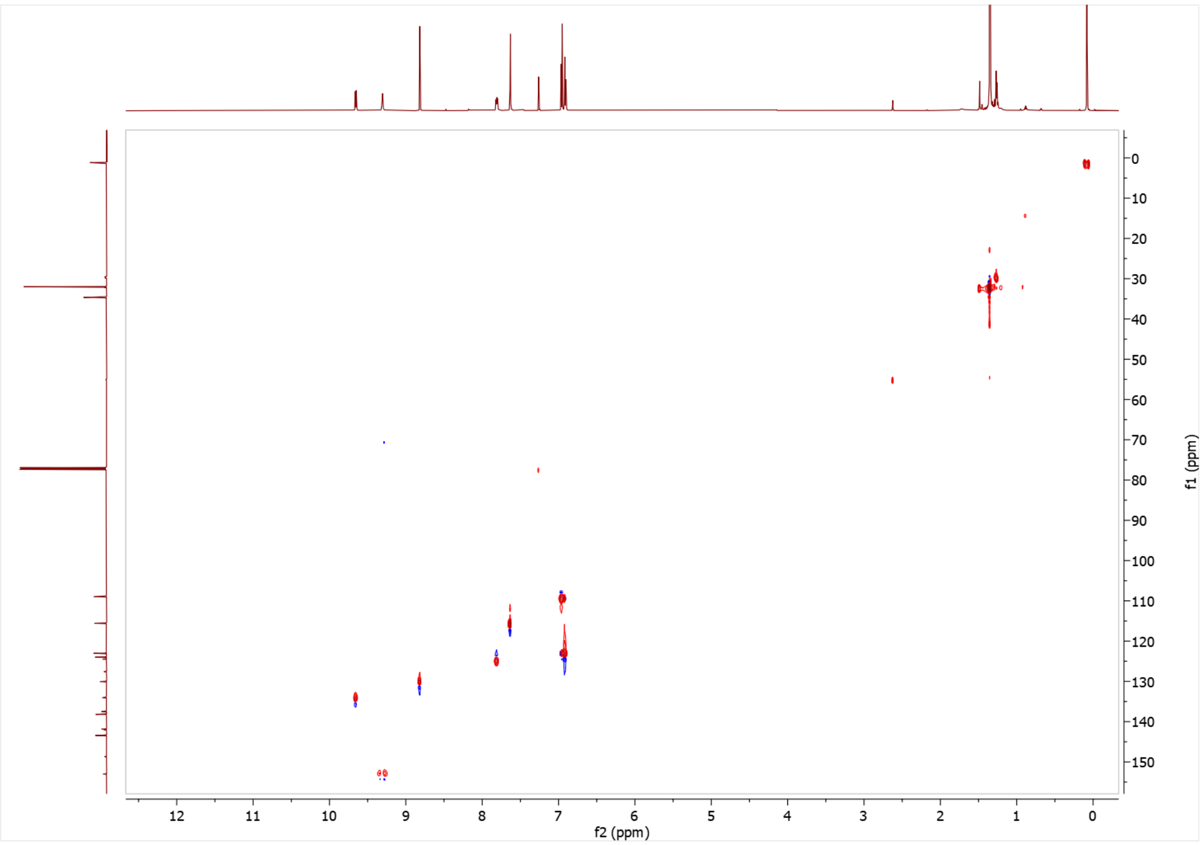

Figure S6: gHSQC 2D NMR spectrum of 2^{t}BuCzDPPZ ($CDCl_3$, 600.2 / 150.9 MHz).

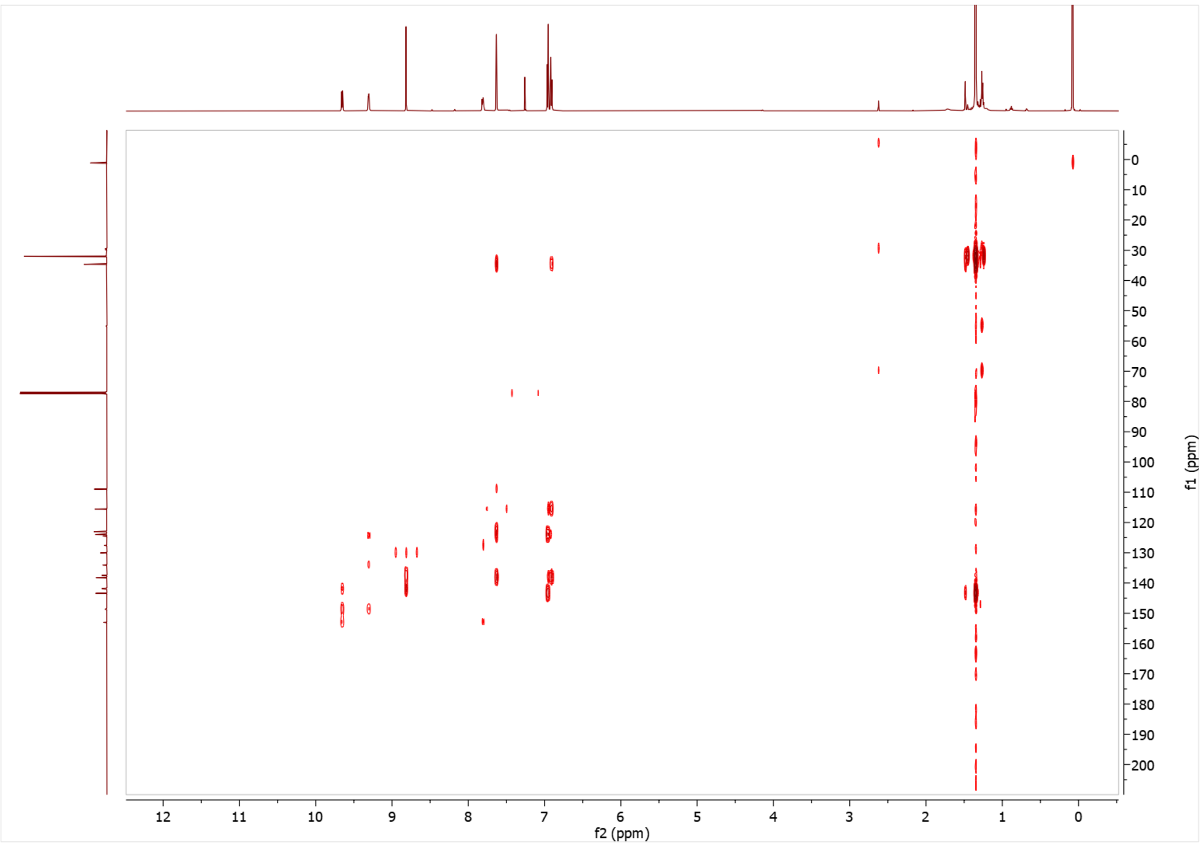

Figure S7: gHMBC 2D NMR spectrum of 2^{t}BuCzDPPZ ($CDCl_3$, 600.2 / 150.9 MHz).

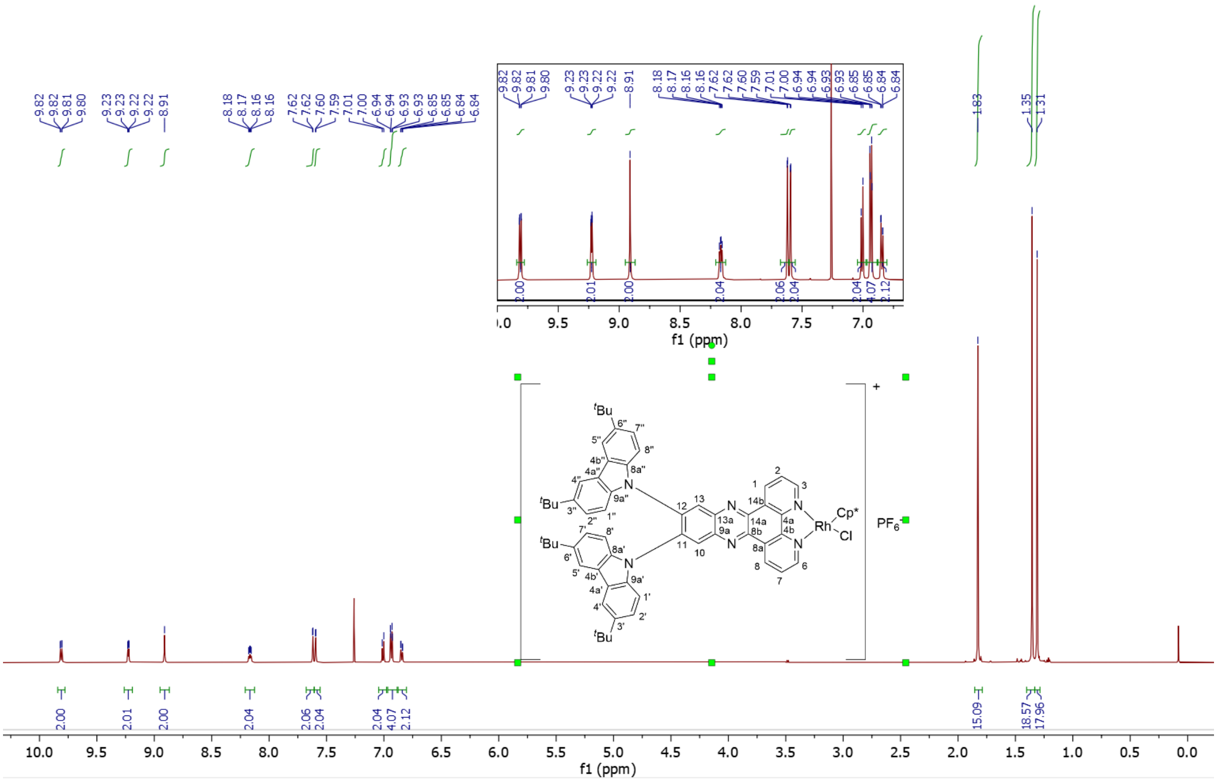

Figure S8: ^{1}H NMR spectrum of 2^{t}BuCzDPPZRhCp* ($CDCl_3$, 600.2 MHz).

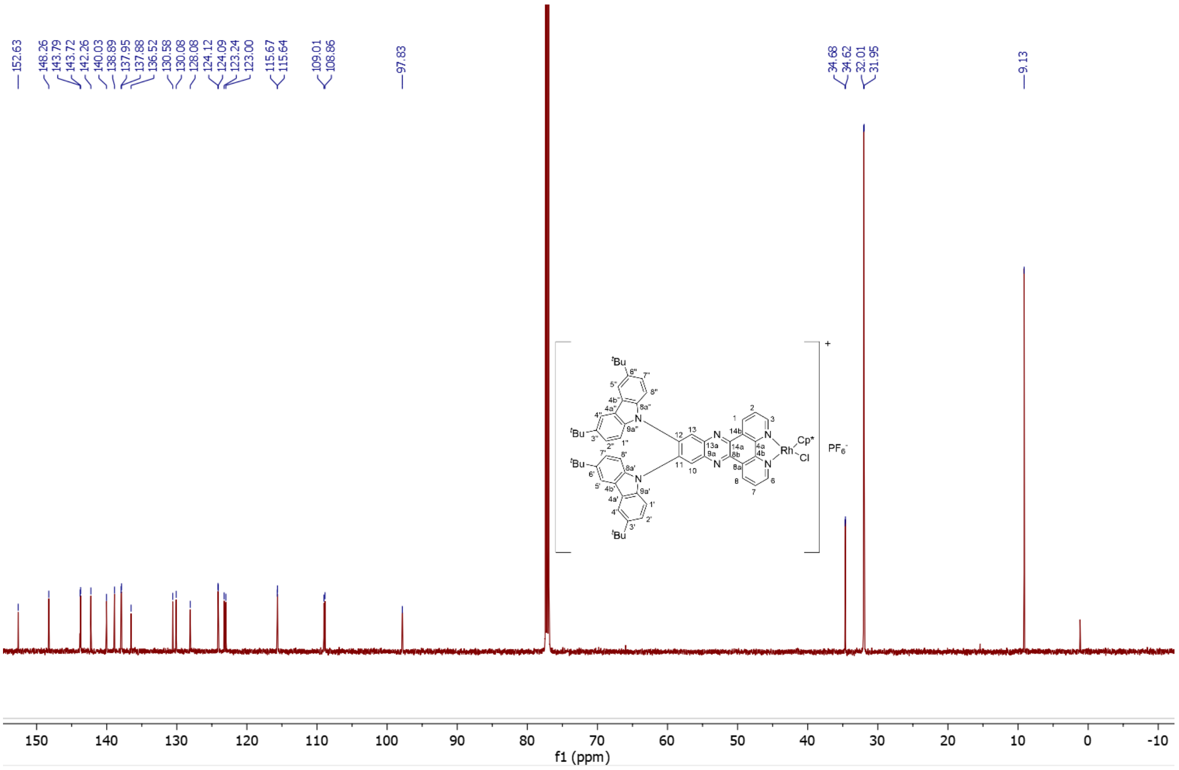

Figure S9: ^{13}C NMR spectrum of 2^{t}BuCzDPPZRhCp* (CDCl$_3$, 150.9 MHz).

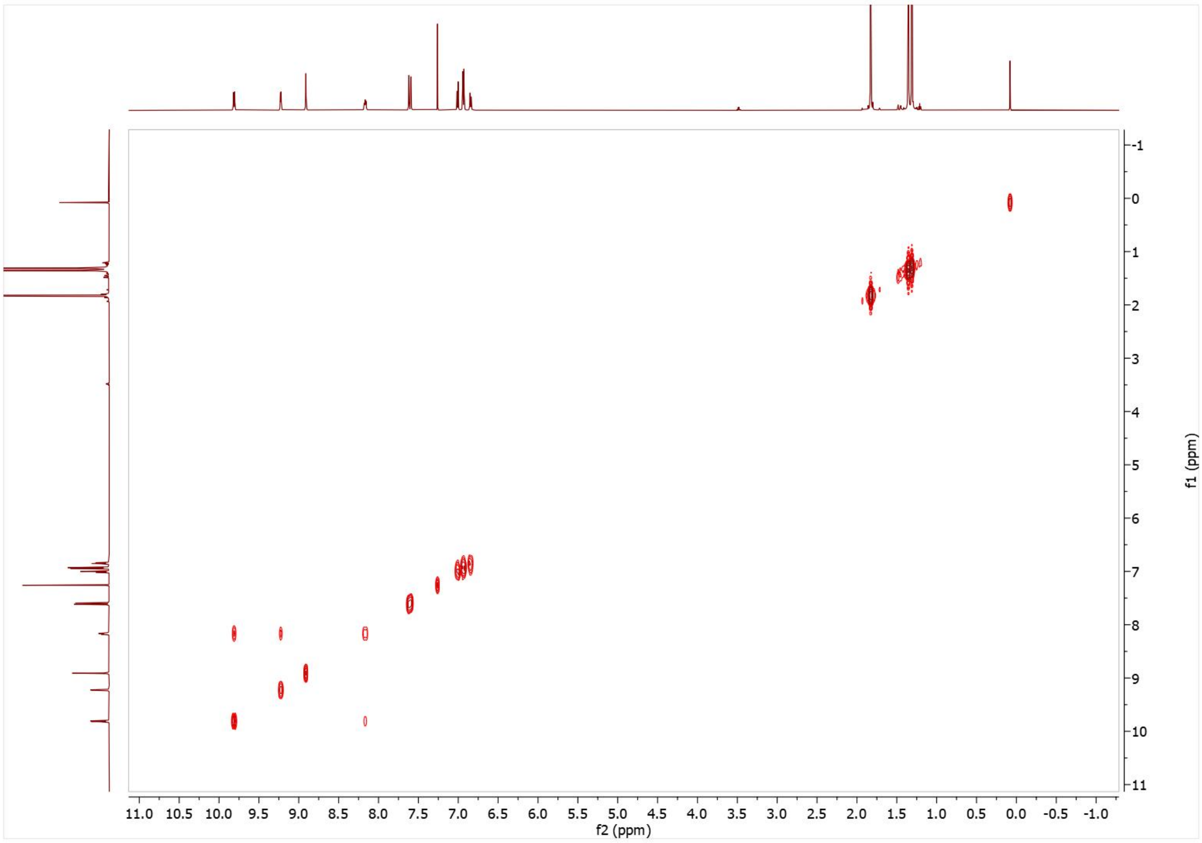

Figure S10: gCOSY 2D NMR spectrum of 2^{t}BuCzDPPZRhCp* (CDCl$_3$, 600.2 / 150.9 MHz).

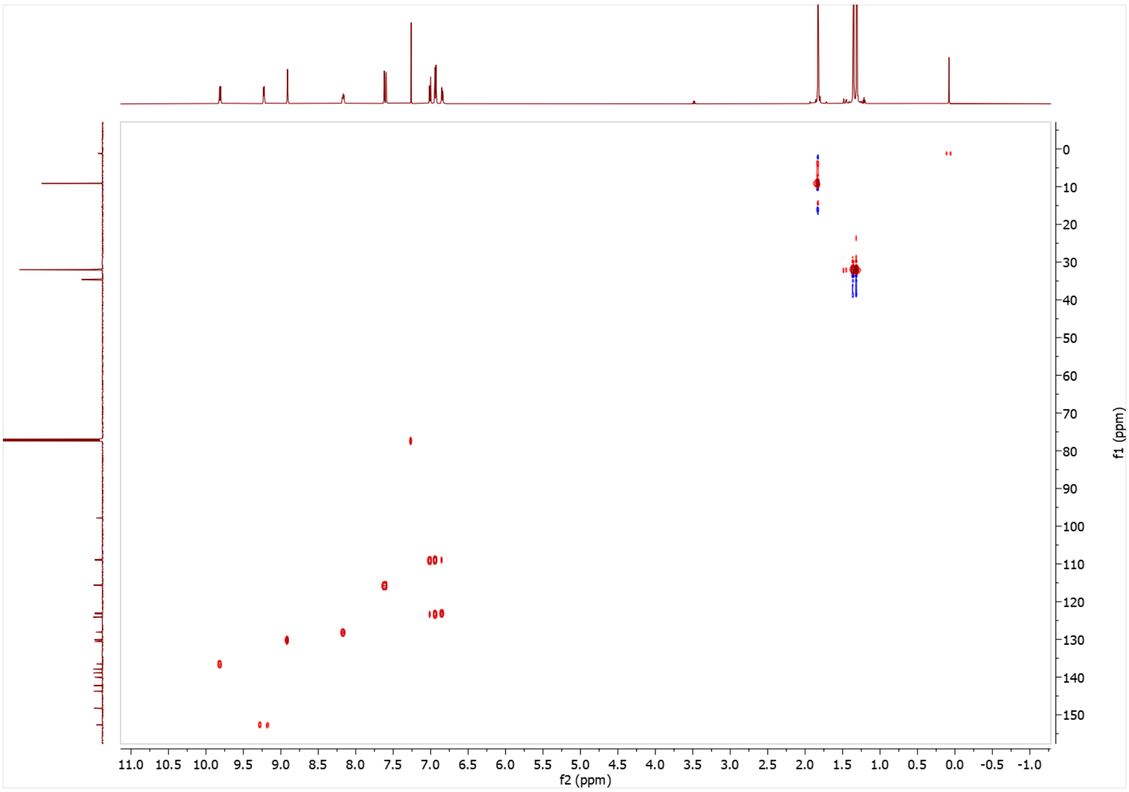

Figure S11: gHSQC 2D NMR spectrum of 2^{t}BuCzDPPZRhCp* ($CDCl_3$, 600.2 / 150.9 MHz).

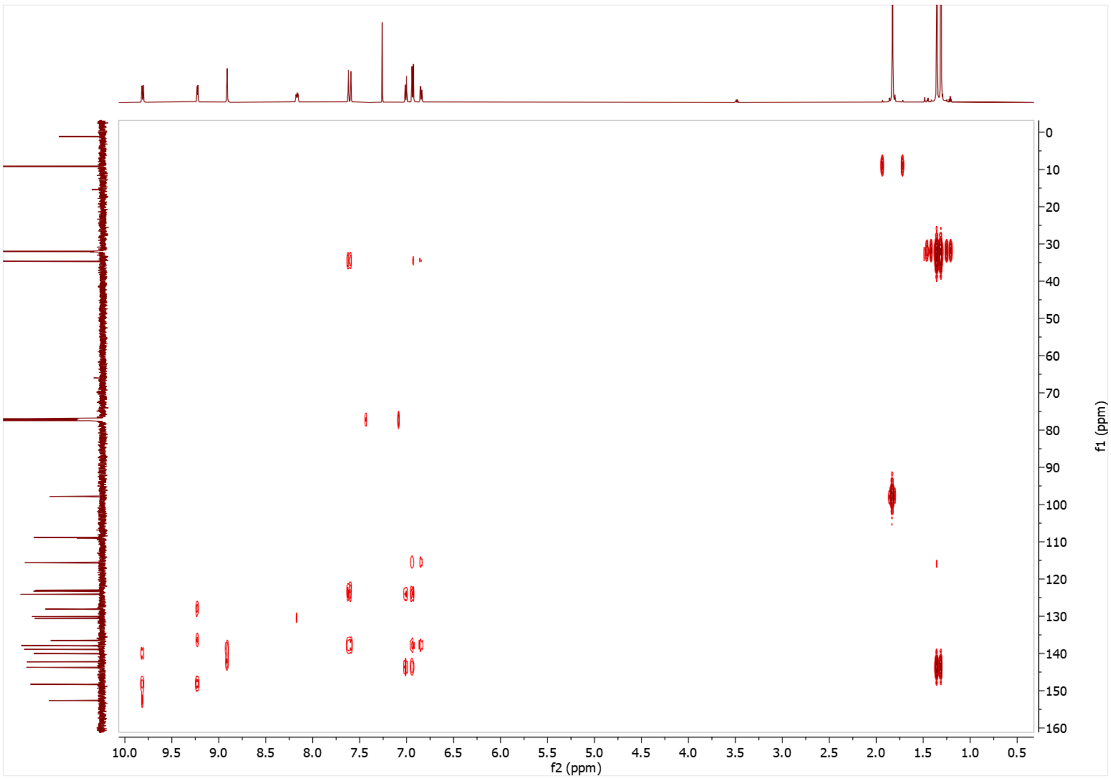

Figure S12: gHMBC 2D NMR spectrum of 2^{t}BuCzDPPZRhCp* ($CDCl_3$, 600.2 / 150.9 MHz).

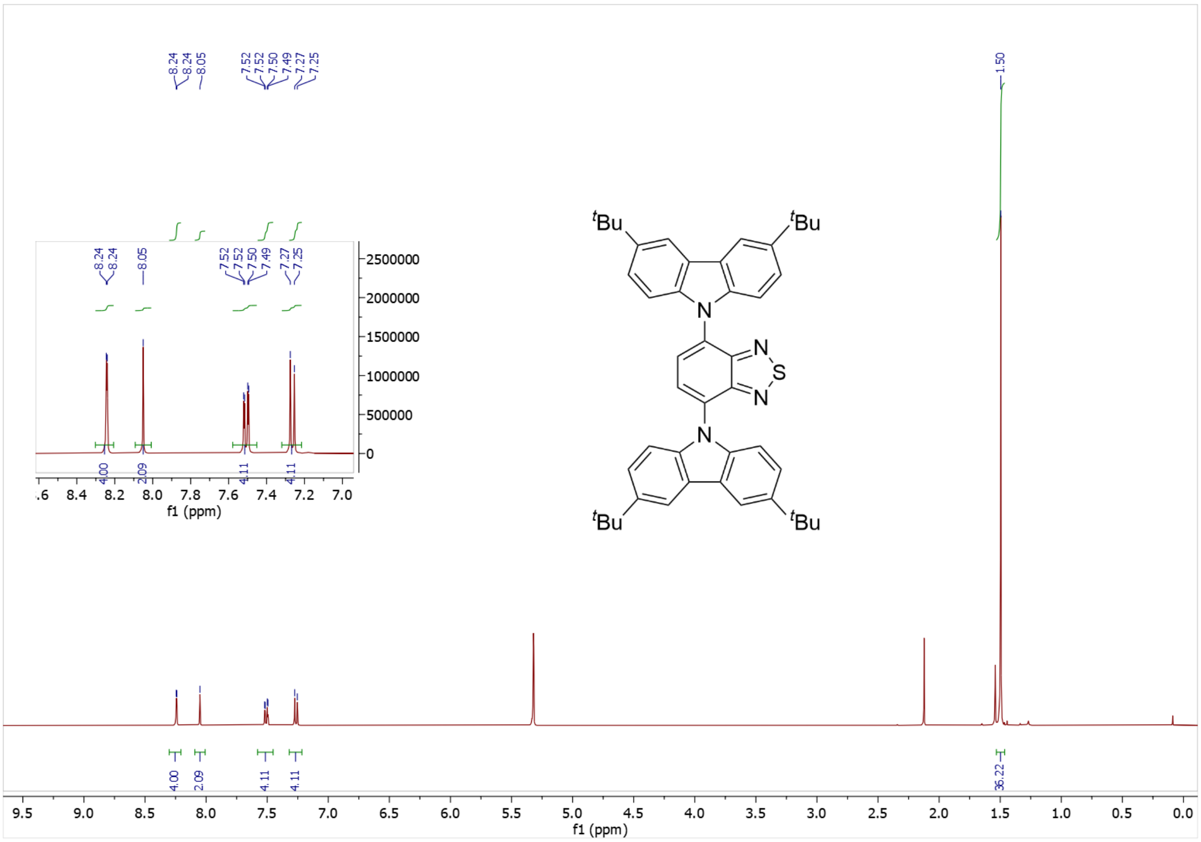

Figure S13: ^{1}H NMR spectrum of 2^{t}BuCzBTD (CD_2Cl_2, 400.1 MHz, δ(acetone) = 2.15 ppm).

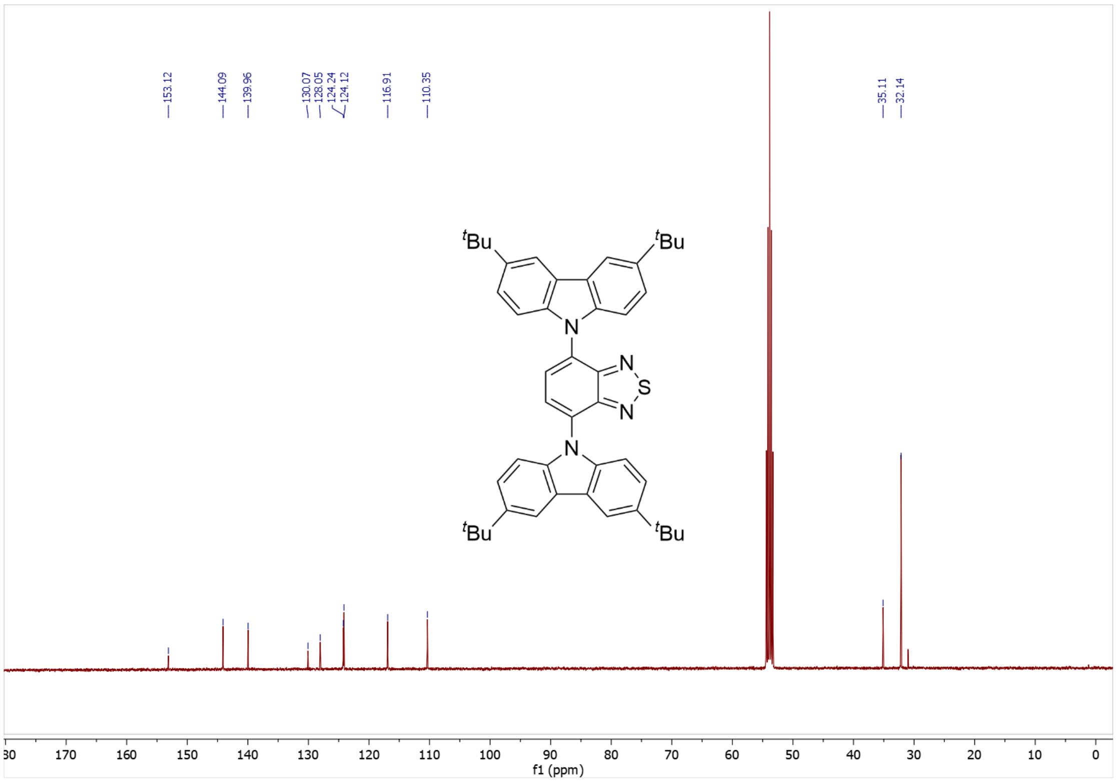

Figure S14: ^{13}C NMR spectrum of 2^{t}BuCzBTD (CD_2Cl_2, 101.6 MHz).

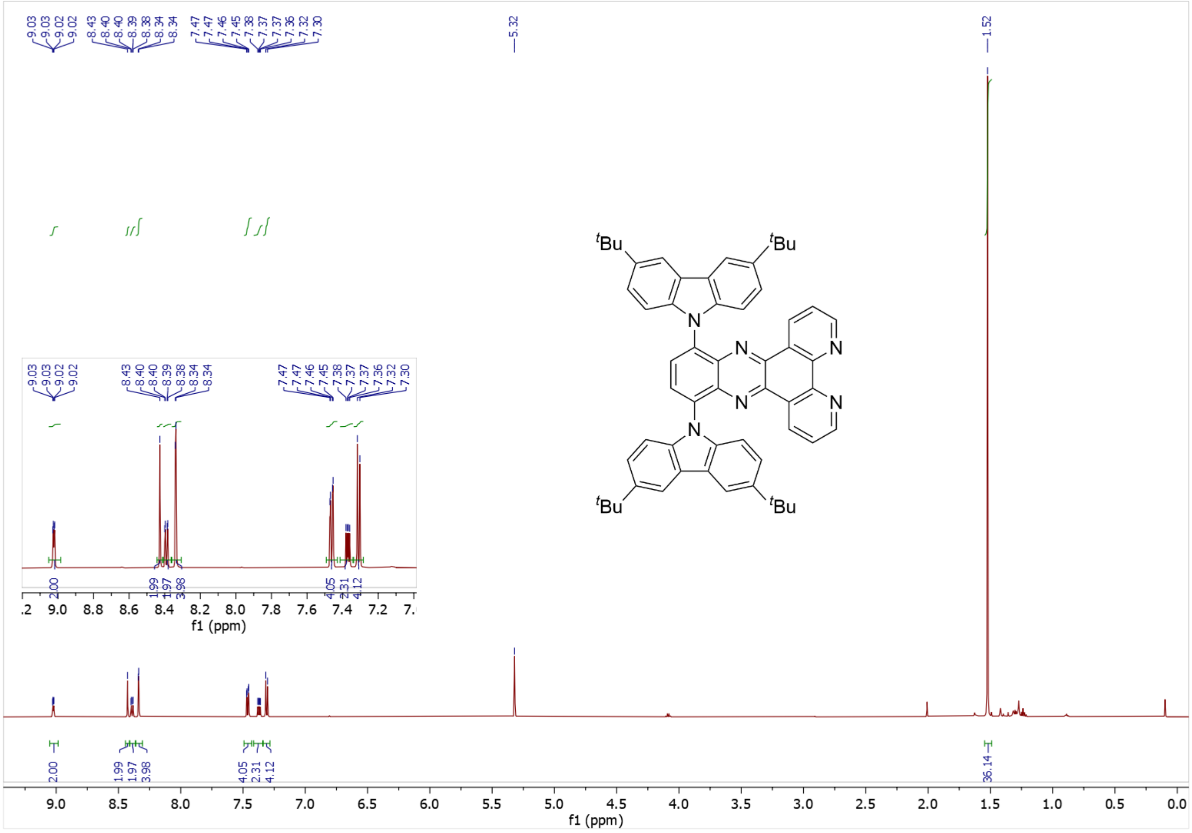

Figure S15: ^{1}H NMR spectrum of *p*2^{t}BuCzDPPZ (CD_2Cl_2, 600.2 MHz).

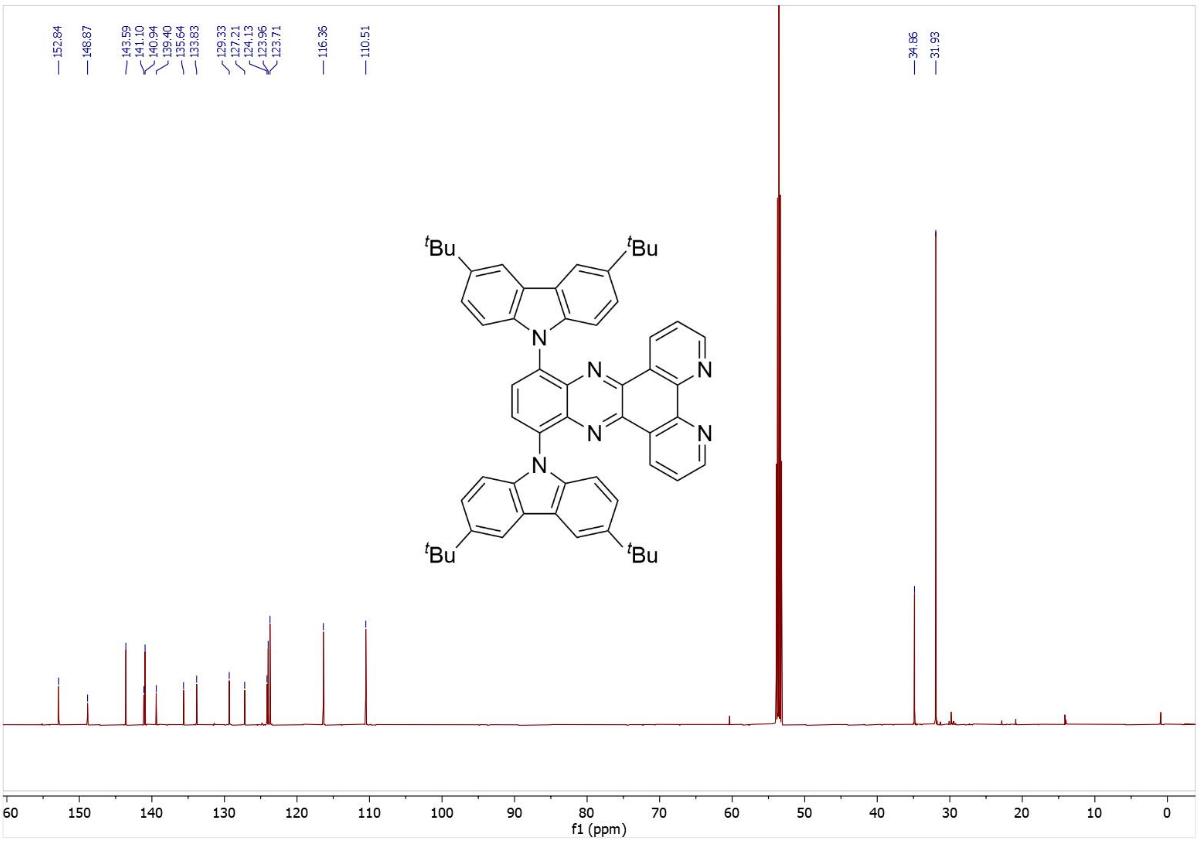

Figure S16: ^{13}C NMR spectrum of *p*2^{t}BuCzDPPZ (CD_2Cl_2, 150.9 MHz).

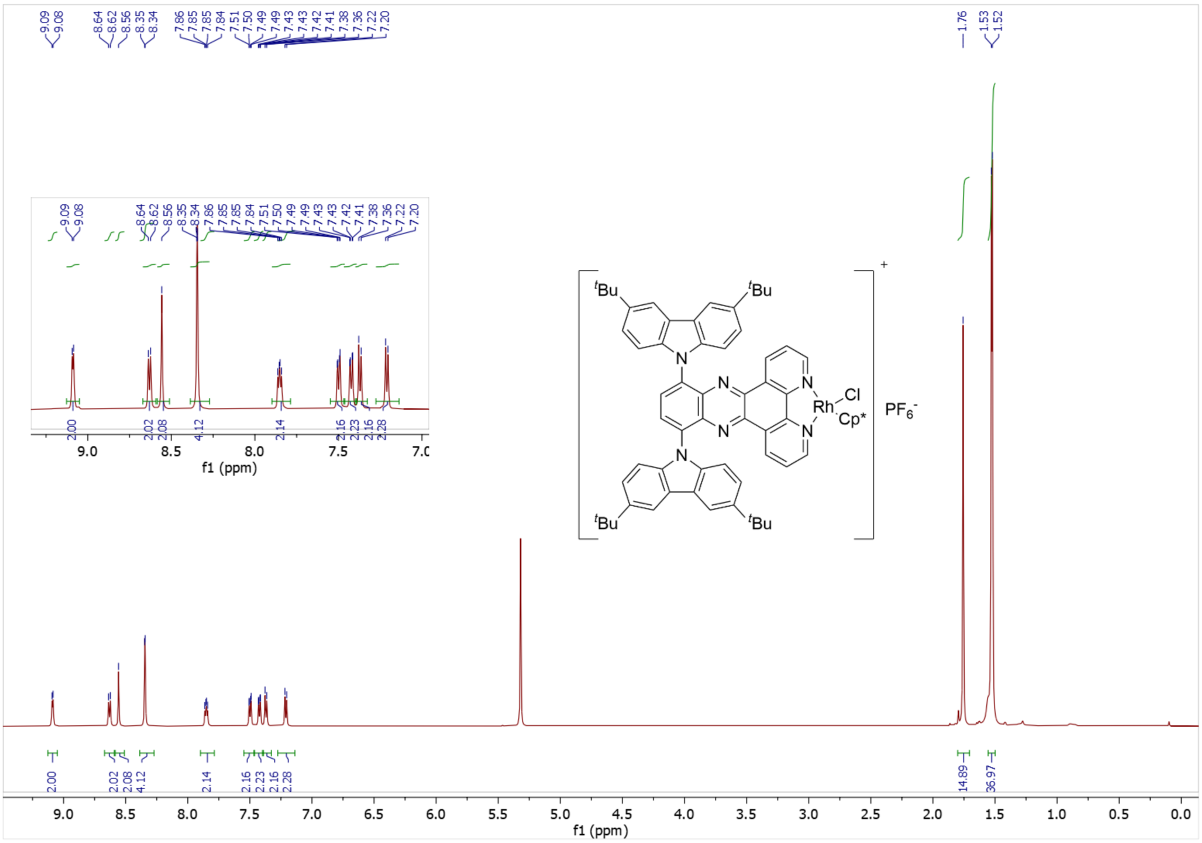

Figure S17: ^{1}H NMR spectrum of *p*2^{t}BuCzDPPZRhCp* (CD_2Cl_2, 600.2 MHz).

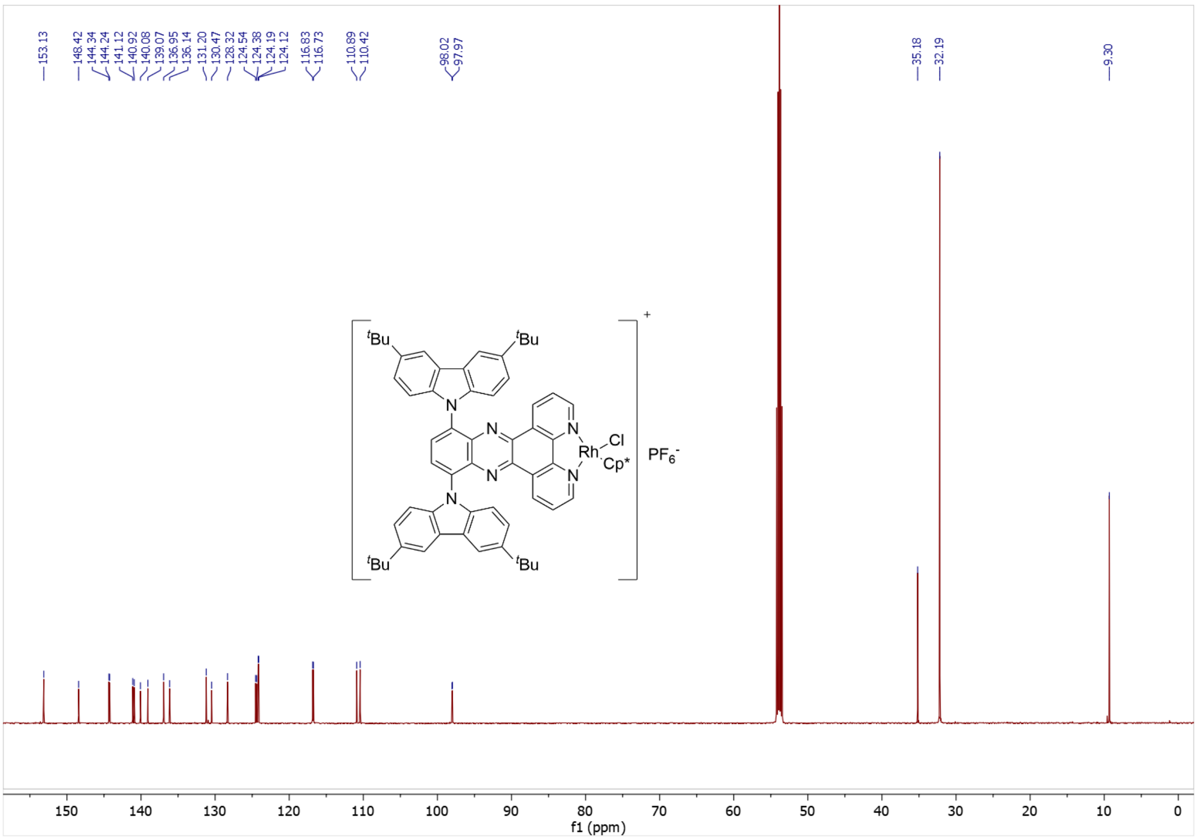

Figure S18: ^{13}C NMR spectrum of *p*2^{t}BuCzDPPZRhCp* (CD_2Cl_2, 150.9 MHz).

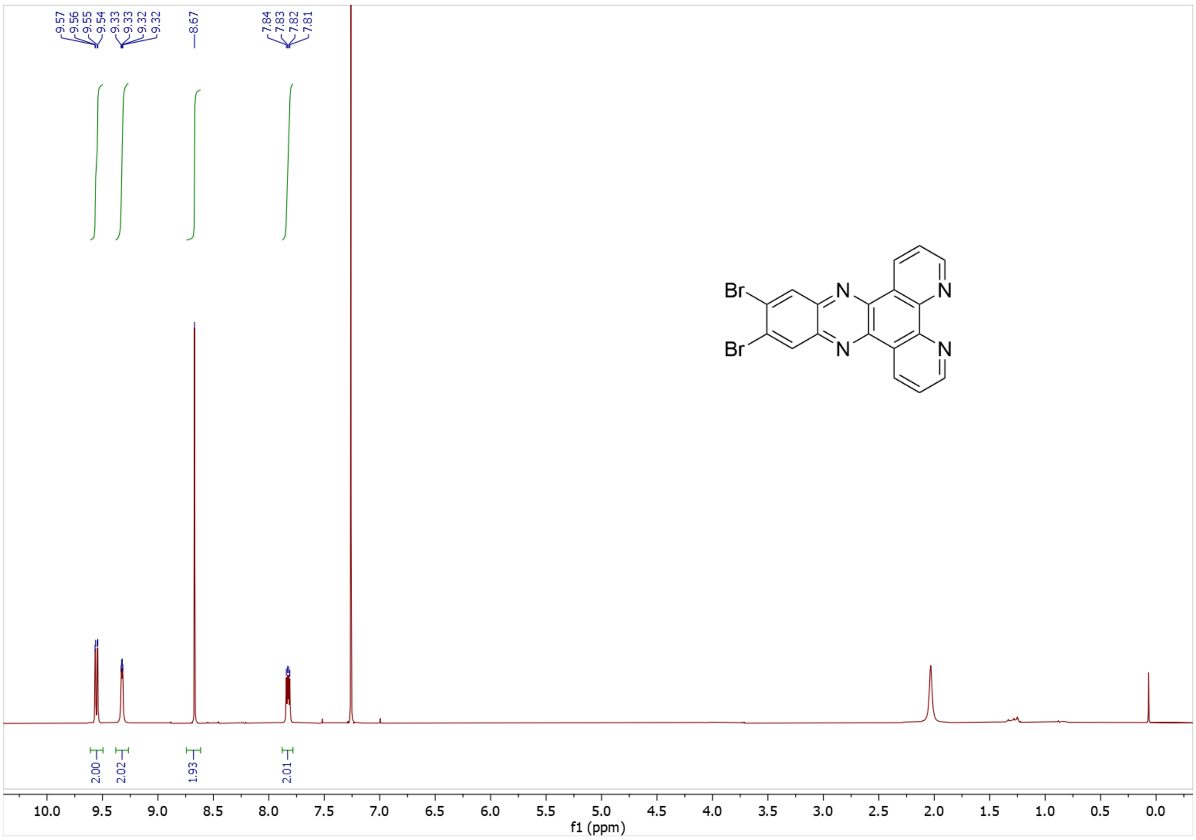

Figure S19: ^{1}H NMR spectrum of 2BrDPPZ ($CDCl_3$, 400.1 MHz).

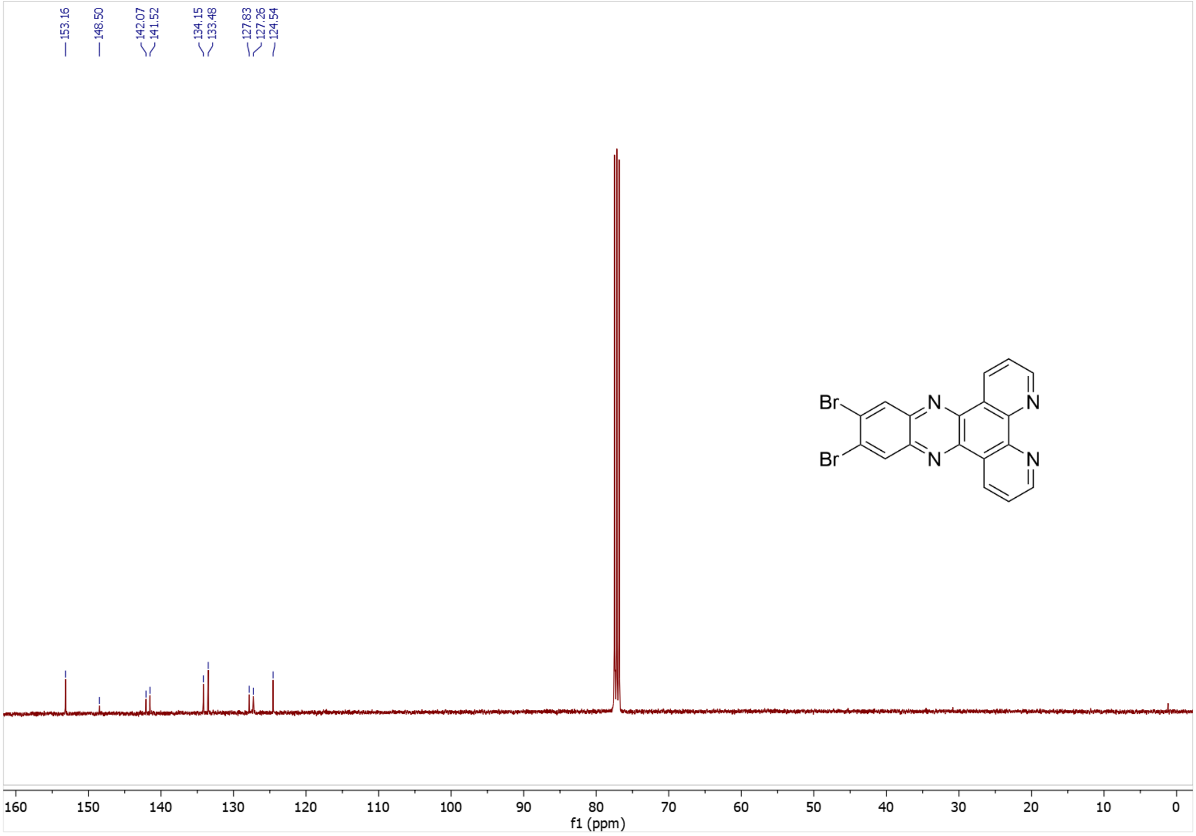

Figure S20: ^{13}C NMR spectrum of 2BrDPPZ ($CDCl_3$, 101.6 MHz).

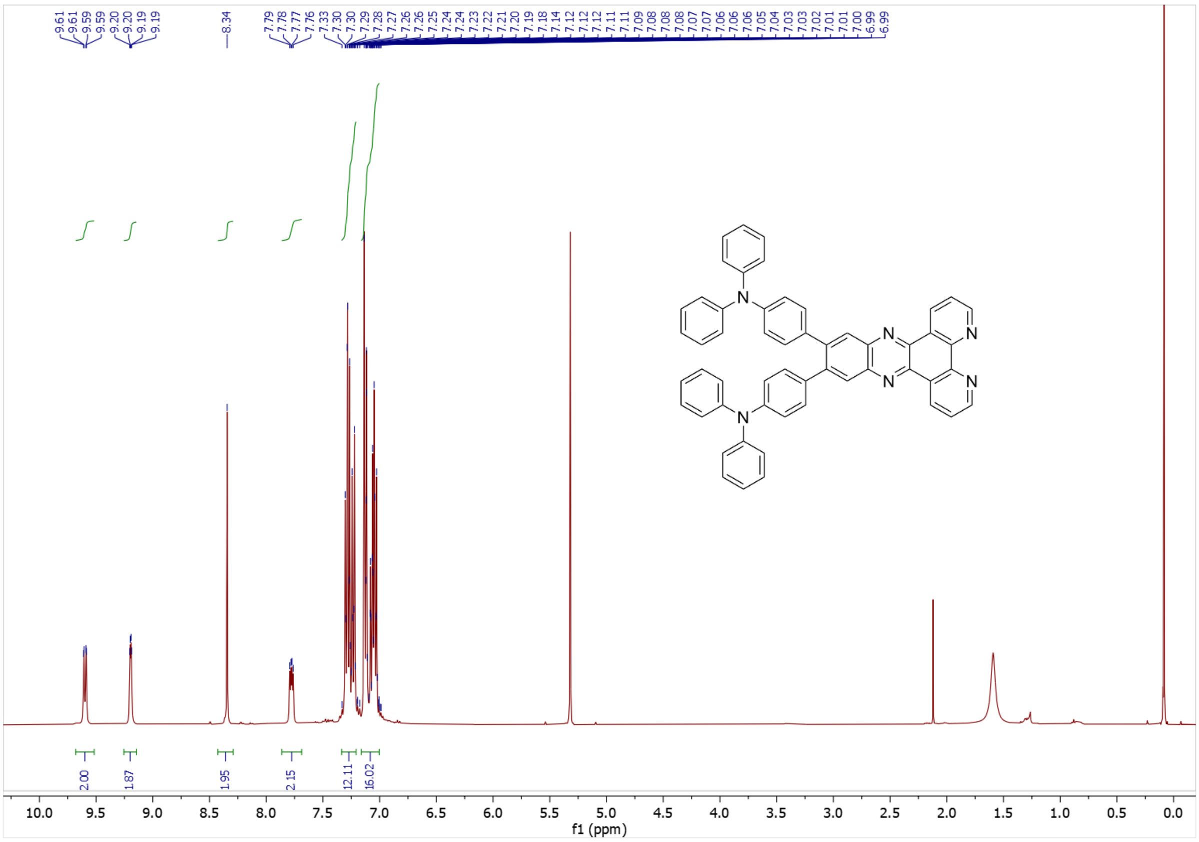

Figure S21: ^{1}H NMR spectrum of 2TPADPPZ (CD_2Cl_2, 400.1 MHz).

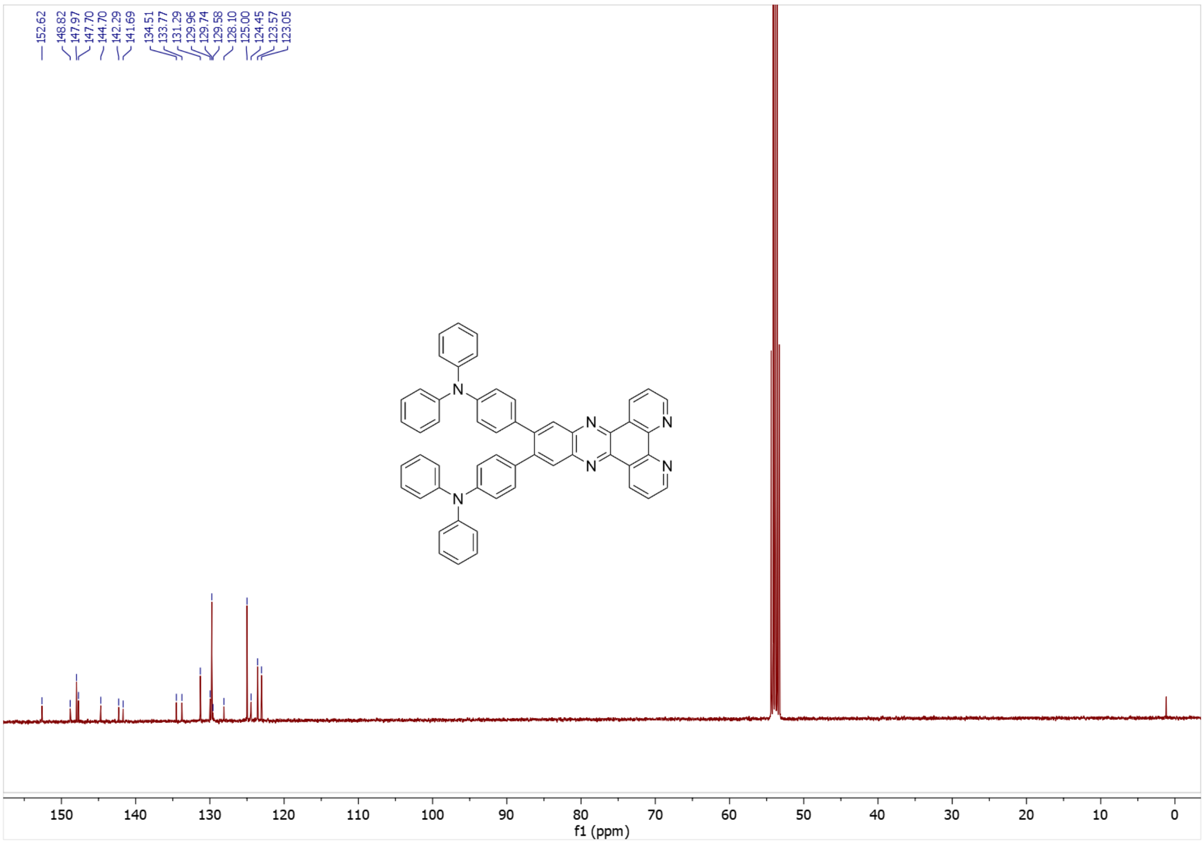

Figure S22: ^{13}C NMR spectrum of 2TPADPPZ (CD_2Cl_2, 101.6 MHz).

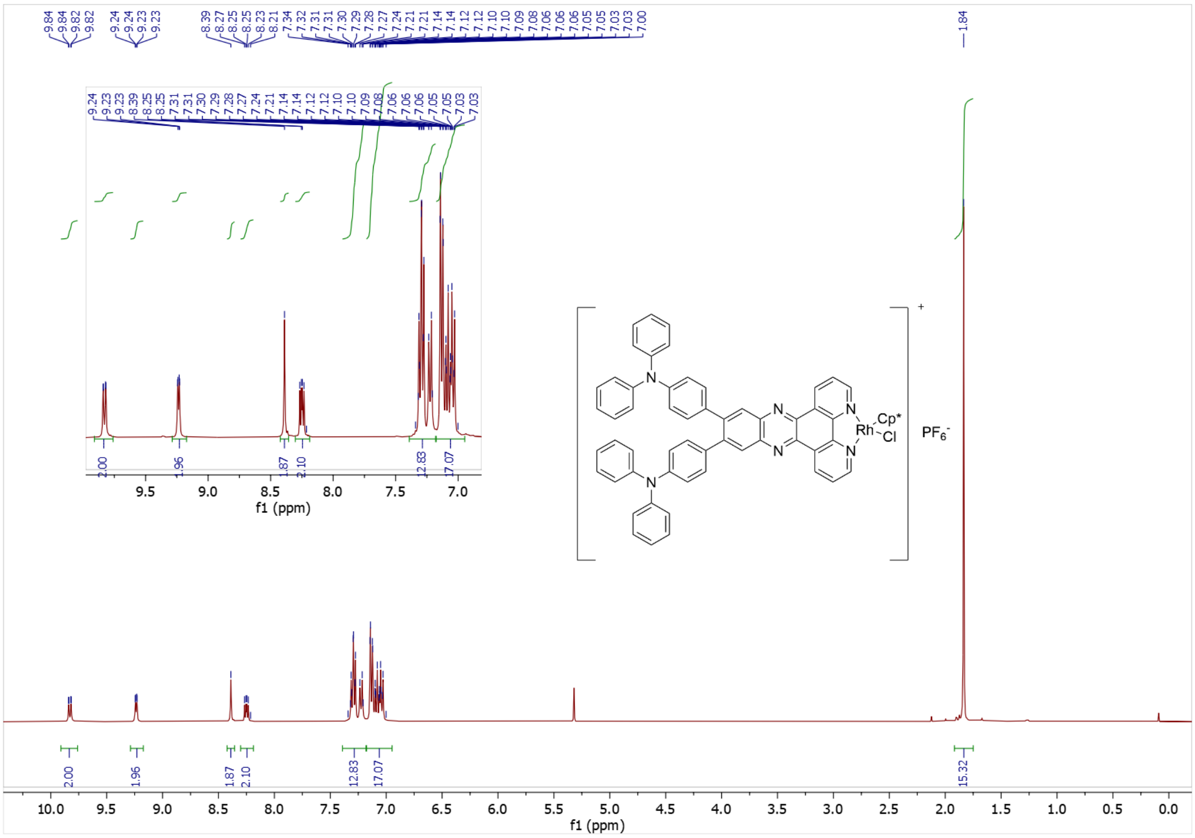

Figure S23: ^{1}H NMR spectrum of 2TPADPPZRhCp* (CD_2Cl_2, 600.2 MHz).

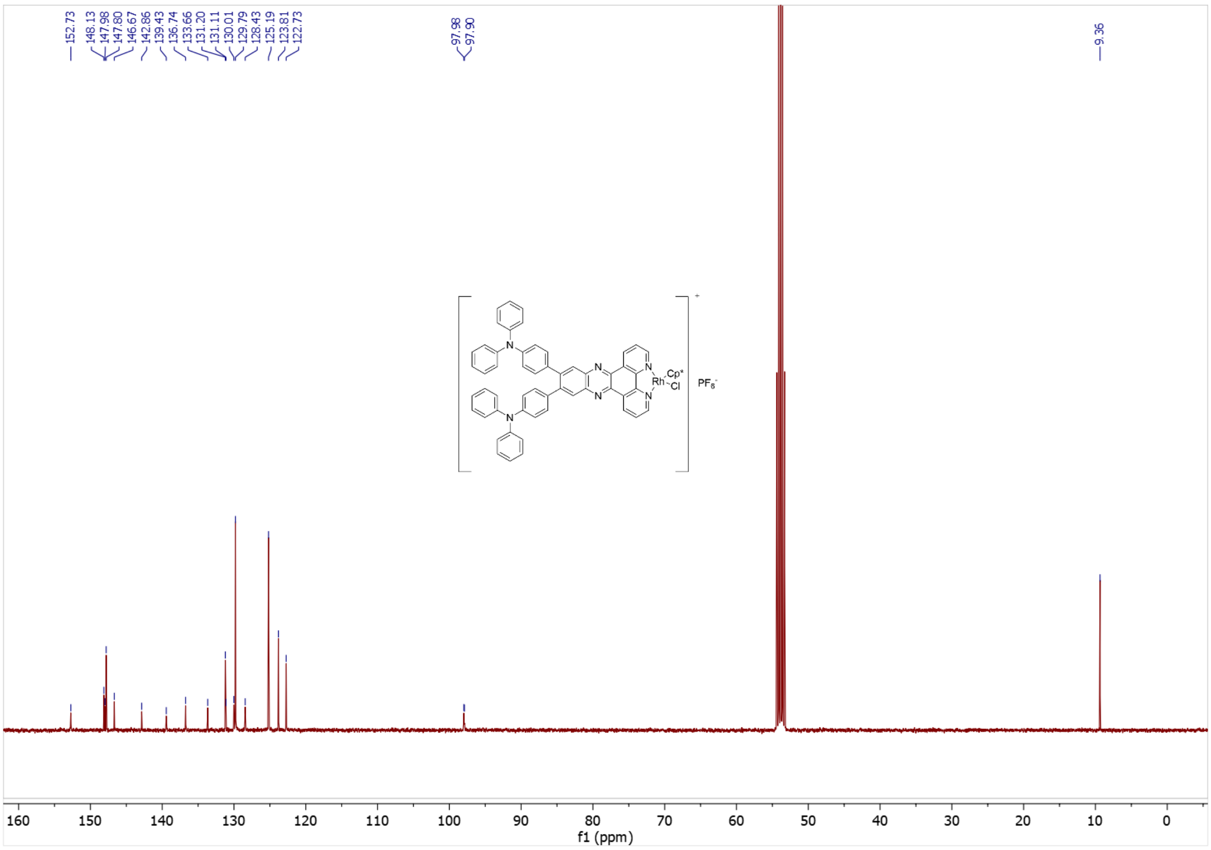

Figure S24: ^{13}C NMR spectrum of 2TPADPPZRhCp* (CD_2Cl_2, 150.9 MHz).

4. Cyclic Voltammograms

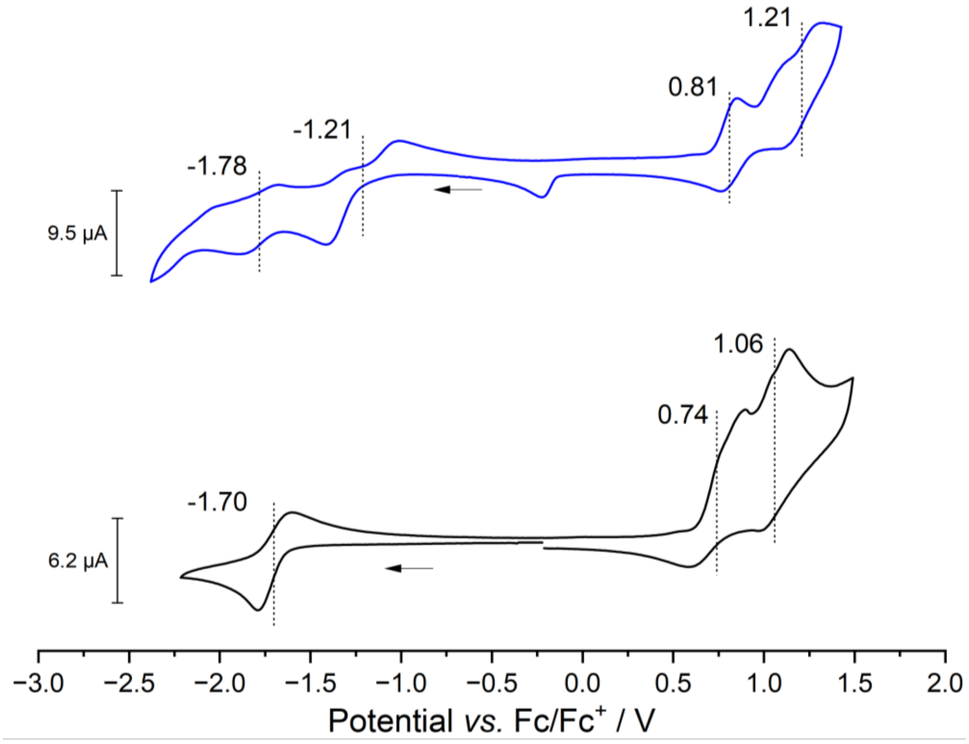

Figure S25: Cyclic voltammograms of 2^{t}BuCzDPPZ (black line) and 2^{t}BuCzDPPZRhCp* (blue line) in CH_2Cl_2 (1 mM) with 0.1 M *n*-Bu_4NPF_6, WE = GC.

5. Photophysical Measurements

5.1. Transient Absorption Spectroscopy

5.2.1 Excitation at 400 nm

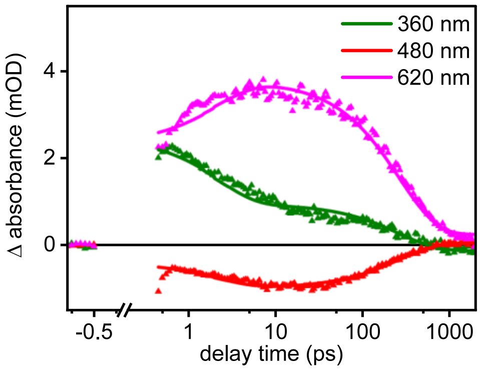

Figure S26. Decay kinetics of 2tBuCzDPPZ in acetonitrile following excitation at 400 nm with respect to their delay time in ps.

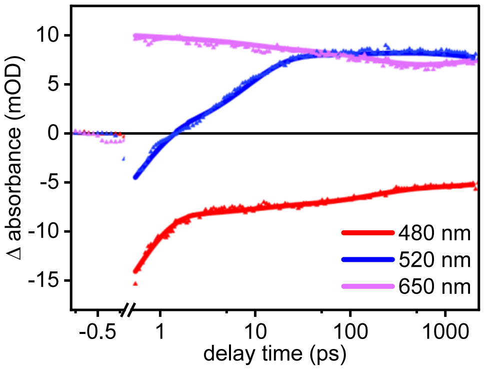

Figure S27. Decay kinetics of 2tBuCzDPPZ in Toluene following excitation at 400 nm with respect to their delay time in ps.

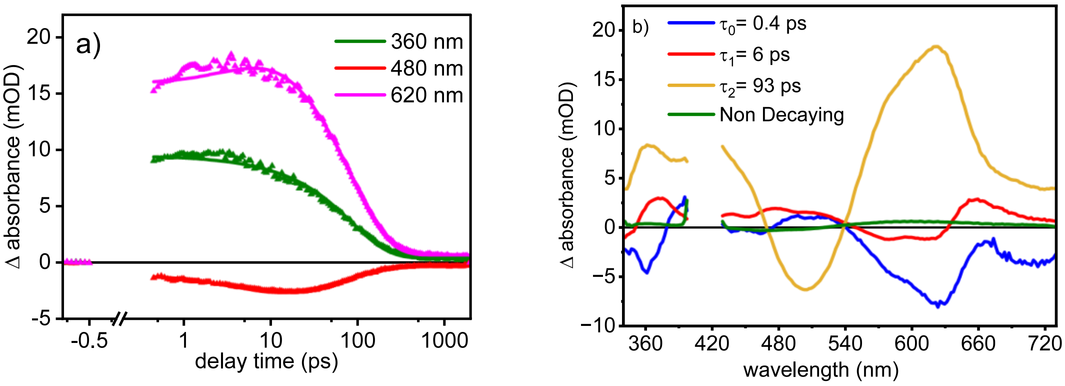

Figure S28. a) Decay kinetics of 2tBuCzDPPZRhCp* in acetonitrile following excitation at 400 nm. concerning their delay time in ps. b) Decay-associated spectra (DAS) in the wavelength range of 350 to 740 nm.

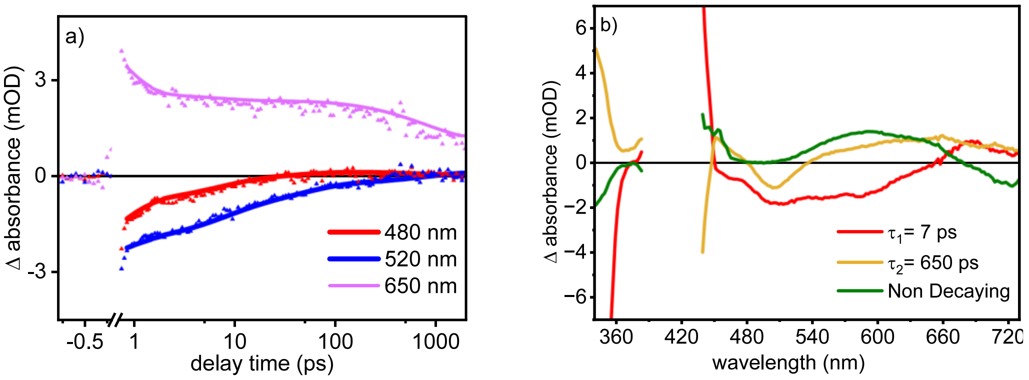

Figure S29. a) Decay kinetics of 2tBuCzDPPZRhCp* in Toluene following excitation at 400 nm. concerning their delay time in ps. b) Decay-associated spectra (DAS) in the wavelength range of 350 to 740 nm.

5.2.2 Excitation at 470 nm

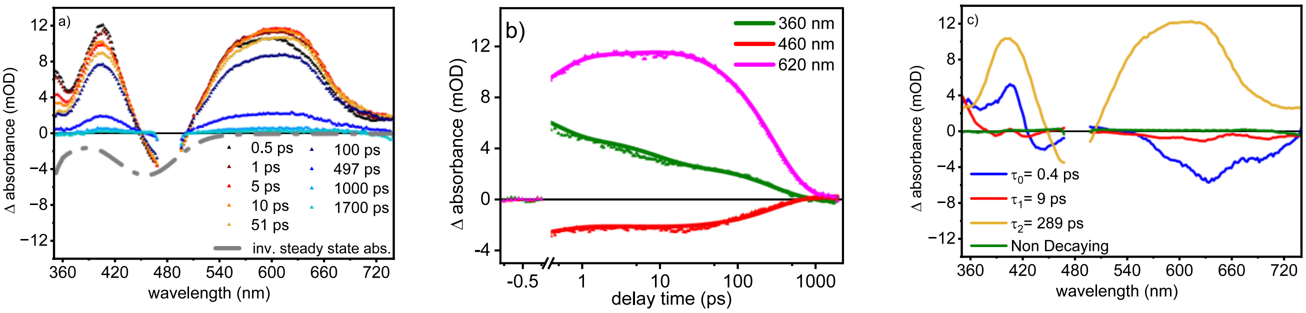

Figure S30. a) Transient absorption spectra of 2tBuCzDPPZ in acetonitrile following excitation at 470 nm. B) Decay kinetics concerning their delay time in ps. C) Decay-associated spectra (DAS) in the wavelength range of 350 to 740 nm.

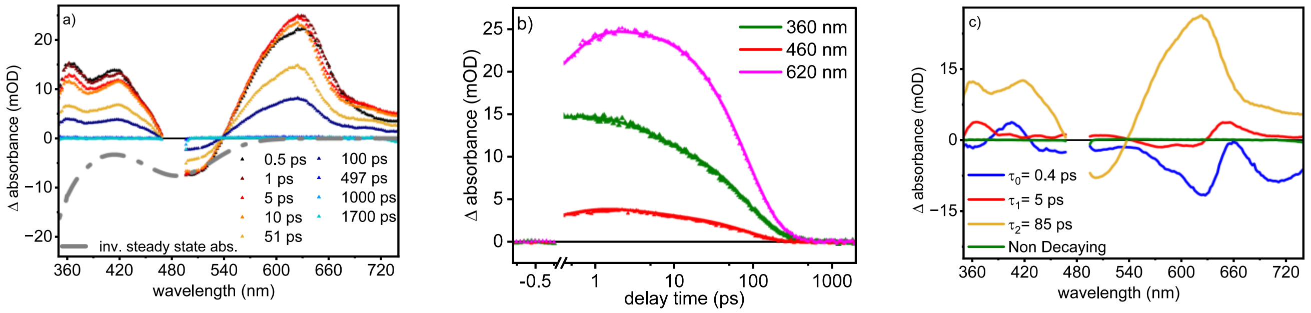

Figure S31. a) Transient absorption spectra of 2tBuCzDPPZRhCp* in acetonitrile following excitation at 470 nm. B) Decay kinetics concerning their delay time in ps. C) Decay-associated spectra (DAS) in the wavelength range of 350 to 740 nm.

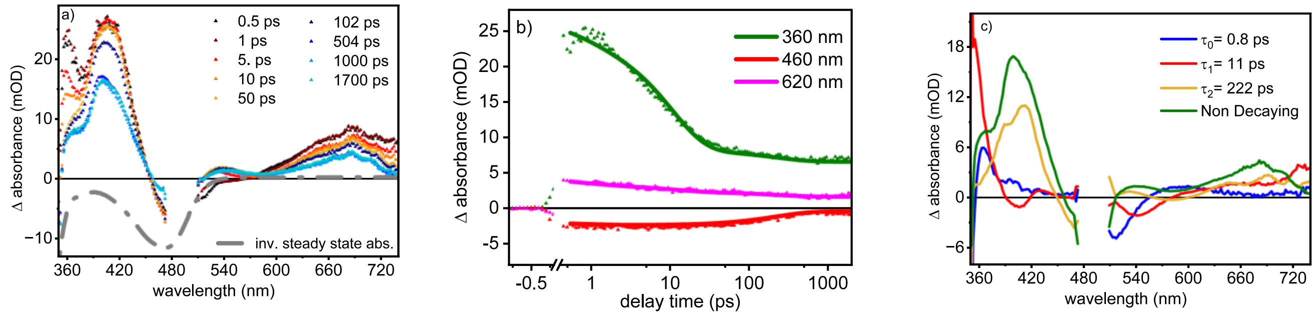

Figure S32. a) Transient absorption spectra of 2tBuCzDPPZ in Toluene following excitation at 470 nm. B) Decay kinetics concerning their delay time in ps. C) Decay-associated spectra (DAS) in the wavelength range of 350 to 740 nm.

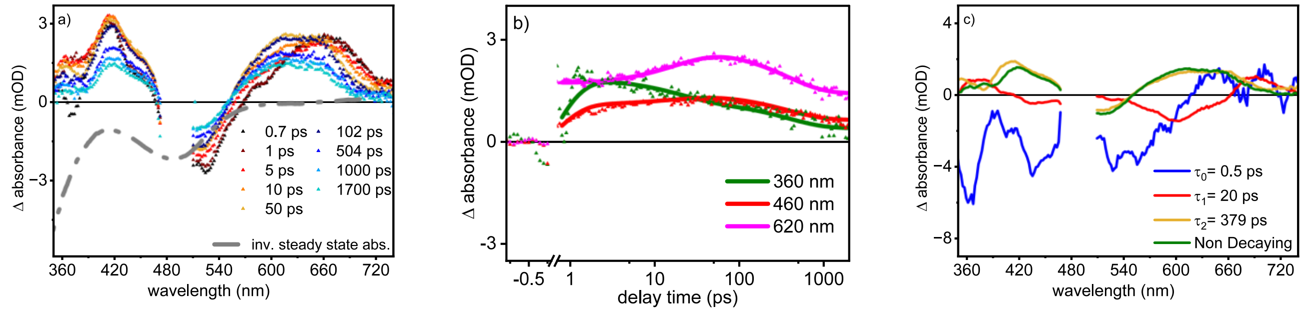

Figure S33. a) Transient absorption spectra of 2tBuCzDPPZRhCp* in Toluene following excitation at 470 nm. B) Decay kinetics concerning their delay time in ps. C) Decay-associated spectra (DAS) in the wavelength range of 350 to 740 nm.

5.2. Determination of ΔE_{ST}

The energy gap between the first singlet and the first triplet state of 2^{t}BuCzDPPZ was calculated based on the λ_{max} values of the fluorescence and the phosphorescence (at 77 K) spectra. Here, the λ_{max} value of the fluorescence spectrum in eV corresponds to the energy of the singlet state and the λ_{max} value of the phosphorescence spectrum (at 77 K) in eV corresponds to the energy of the triplet state.

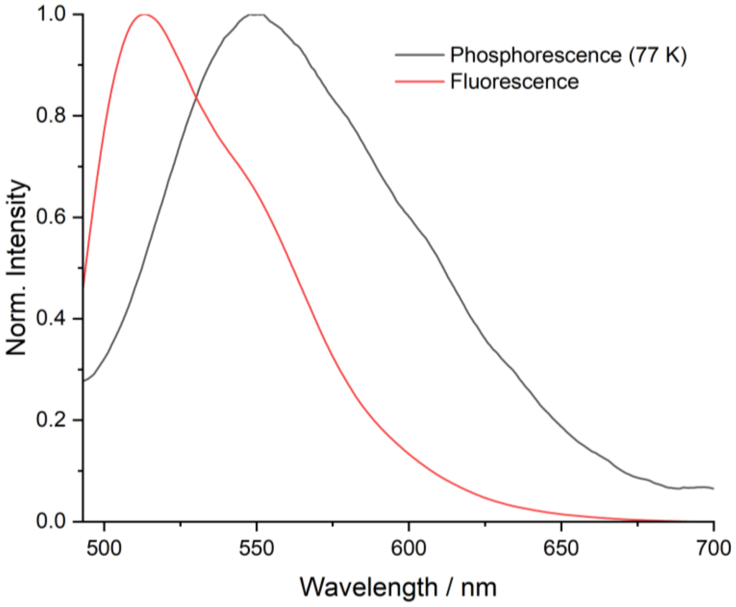

Figure S34. Fluorescence and phosphorescence (at 77 K) spectra of 2^{t}BuCzDPPZ in methylcyclohexane.

The compound was dissolved in methylcyclohexane, and the solution was transferred into an NMR tube, which was cooled to 77 K using liquid nitrogen. The spectrum was recorded immediately afterwards (λ_{ex}: 470 nm).

Formula to calculate the photon energy (eV) from the wavelength (nm):

$$E = \frac{h \cdot c}{\lambda} \qquad eq.\ 4$$

Here, E is the photonenergy in eV, h is the planck constant 4.1257×10^{-15} eV·s, c is the speed of light 299792458 m/s, and λ is the wavelength in nm.

Table S1. λ_{max} values of the fluorescence and phosphorescence (at 77 K) spectra and the corresponding singlet and triplet energies obtained with [1].

λ_{max} (fluorescence)	513.5 nm
λ_{max} (phosphorescence)	548 nm
E_S	2.41 eV
E_T	2.26 eV

Formula to calculate the ΔE_{ST}:

$$\Delta E_{ST}=E_s\text{-}E_T=2.41\ eV\text{-}2.26\ eV=0.15\ eV \qquad eq.\ 5$$

Here, E_s is the energy of the singlet state in eV and E_T is the energy of the triplet state in eV.

5.3. Stern-Volmer Quenching Experiments

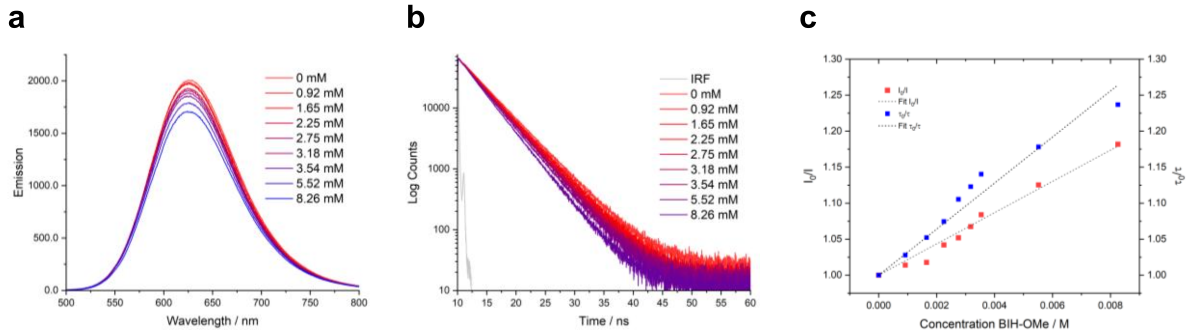

Figure S35: Emission (**a**) and lifetime (**b**) quenching of 2^{t}BuCzDPPZ (8.24 µM) with BIH-OMe. The corresponding Stern-Volmer plots (K_{SV}(emission) = 21.7 M^{-1}, K_{SV}(lifetime) = 32.0 M^{-1}) (**c**).

A stock solution of 2^{t}BuCzDPPZ in dichloromethane with the concentration of 8.24 µM was prepared, and two cuvettes were filled with 2 mL each of this solution. In cuvette 2, 4.2 mg of the quencher BIH-OMe were added to obtain a concentration of 0.0083 M. From both solutions fluorescence and emission lifetime (TCSPC) were measured. In the next steps a certain volume (6 x 250 µL) of the solution in cuvette 2 was transferred into cuvette 1 with a Hamilton syringe, and fluorescence intensity and lifetime were again measured. In this way the concentration of the quencher in cuvette 1 was increased by keeping the concentration of 2^{t}BuCzDPPZ constant. For the last data point an additional amount of 0.71 mg of BIH-OMe was transferred into cuvette 1. As a result, the I_0/I (fluorescence intensity without quencher divided by the intensity with quencher) and τ_0/τ (lifetime without quencher divided by the lifetime with quencher) were plotted against the concentration of BIH-OMe.

5.4. Emission Decay

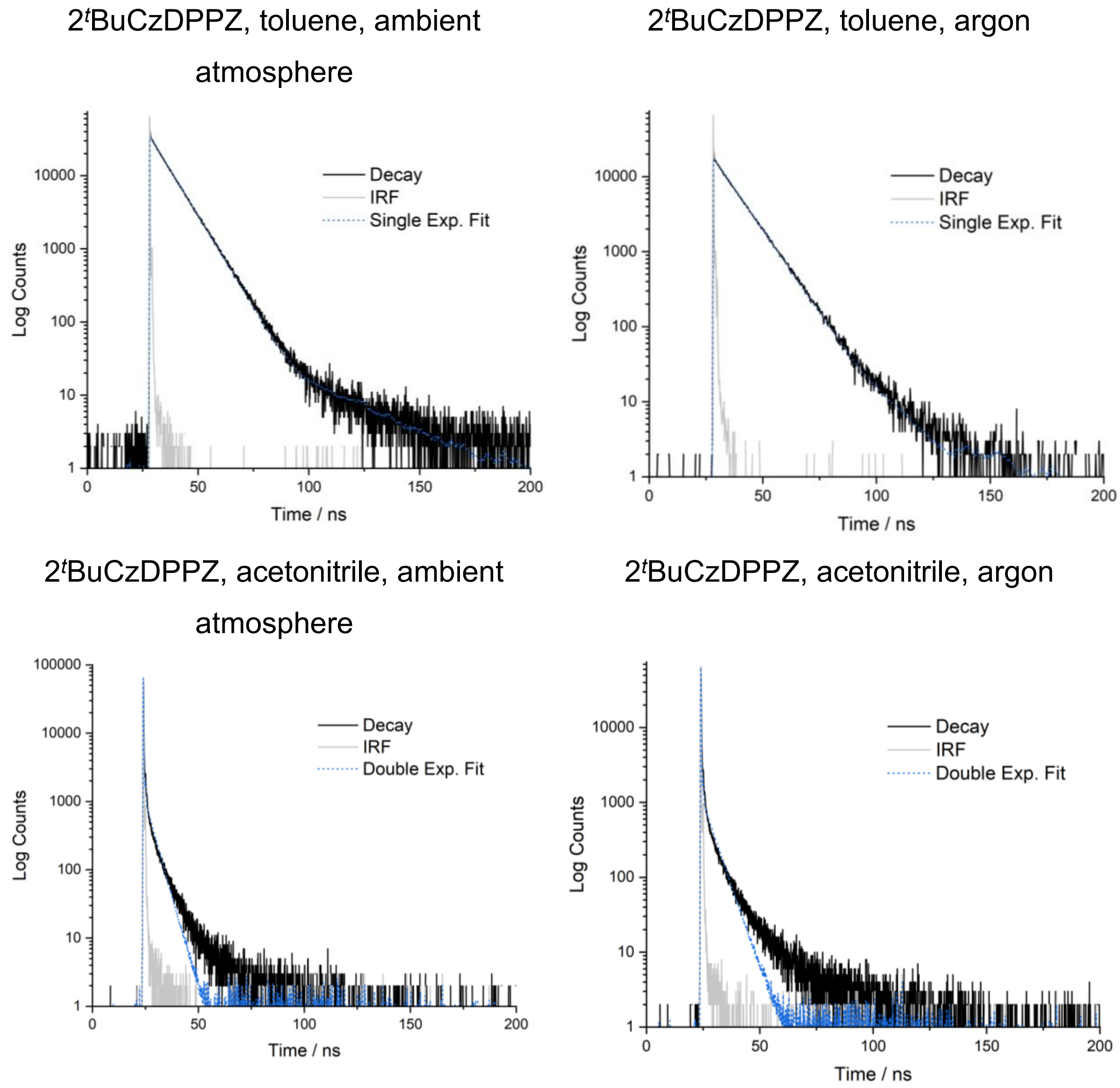

Figure S36: Emission decays (TCSPC) of 2^{t}BuCzDPPZ.

2^{t}BuCzDPPZRhCp*, toluene, ambient atmosphere

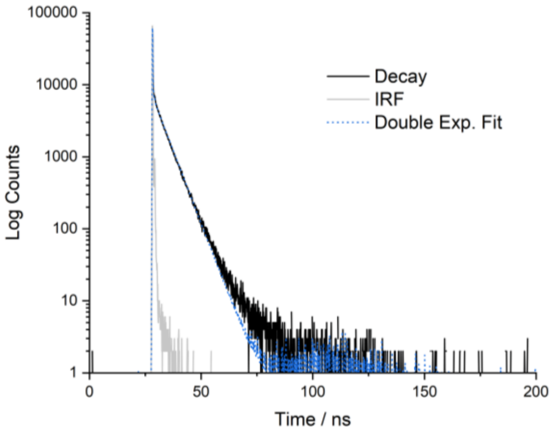

2^{t}BuCzDPPZRhCp*, toluene, argon

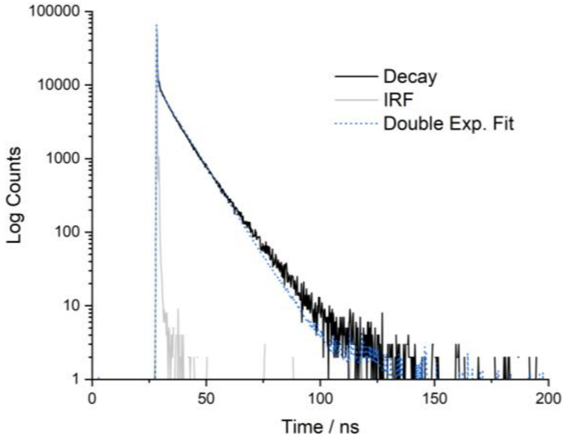

2^{t}BuCzDPPZRhCp*, acetonitrile, ambient atmosphere

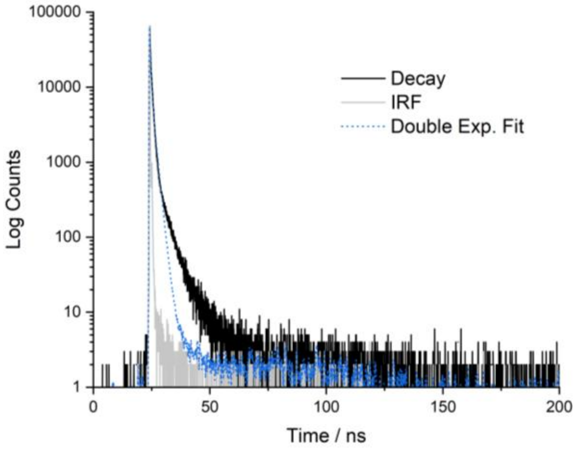

2^{t}BuCzDPPZRhCp*, acetonitrile, argon

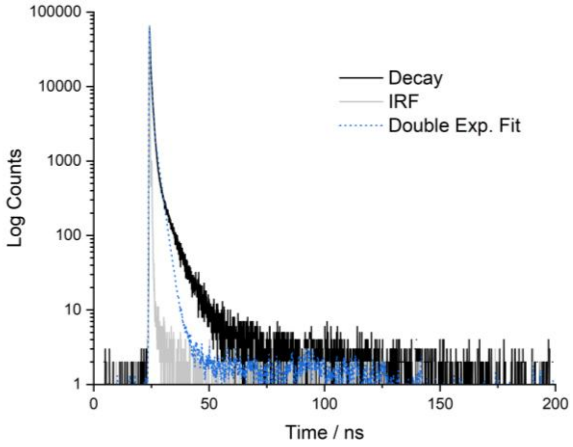

Figure S37: Emission decays (TCSPC) of 2^{t}BuCzDPPZRhCp*.

6. Catalysis

The catalytic procedures described in the following were carried out similar to previously reported experiments.[33,34]

6.1. Determination of NADH Formation

For the determination of the amount of NADH formed, fluorescence spectroscopy was used since NADH features a characteristic emission band at around 460 nm when excited at 340 nm.[33] 2 mL of an NADH stock solution (43.3 μM, acetonitrile/water (1/9, v/v)) was placed in a fluorescence cuvette, then a fluorescence spectrum was recorded by excitation at 340 nm. Afterwards, 1 mL of this solution was replaced with 1 mL solvent (acetonitrile/water) resulting in a solution half as concentrated. Another spectrum was then recorded. In this manner the following concentrations were measured: 43.3 μM, 21.6 μM, 10.8 μM, 5.4 μM, 2.7 μM, and 1.4 μM. Since the purity of the purchased NADH was 97%, the obtained emission intensity was multiplied by 0.97. When the concentration was plotted against the emission, a polynomic trend could be calculated giving the following formula with a R^2 of 0.9999:

$$c=4\cdot 10^{-7}\cdot I^2+0.0058\cdot I-0.0904 \qquad eq.\ 6$$

Here c is the concentration of NADH in μM and I is the emission intensity of NADH multiplied by 0.97.

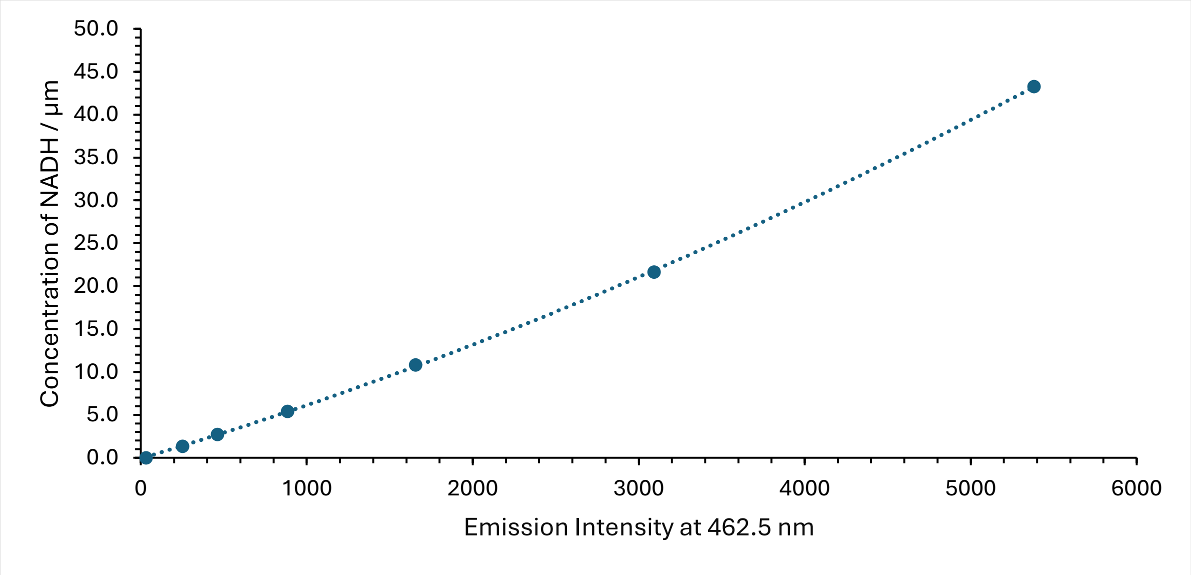

Figure S38: Polynomic trend between concentration and emission intensity of NADH in acetonitrile/water (1/9, v/v).

With this formula all concentrations of NADH in the solvent mixture acetonitrile/water (1/9, v/v) were calculated.

For the solvent mixture acetonitrile/water (1/2, v/v) a similar procedure was applied resulting in the following formula with a R^2 of 1:

$$c = 3 \cdot 10^{-7} \cdot I^2 + 0.005 \cdot I - 0.199 \qquad eq.\ 7$$

Here c is the concentration of NADH in μM and I is the emission intensity of NADH multiplied by 0.97.

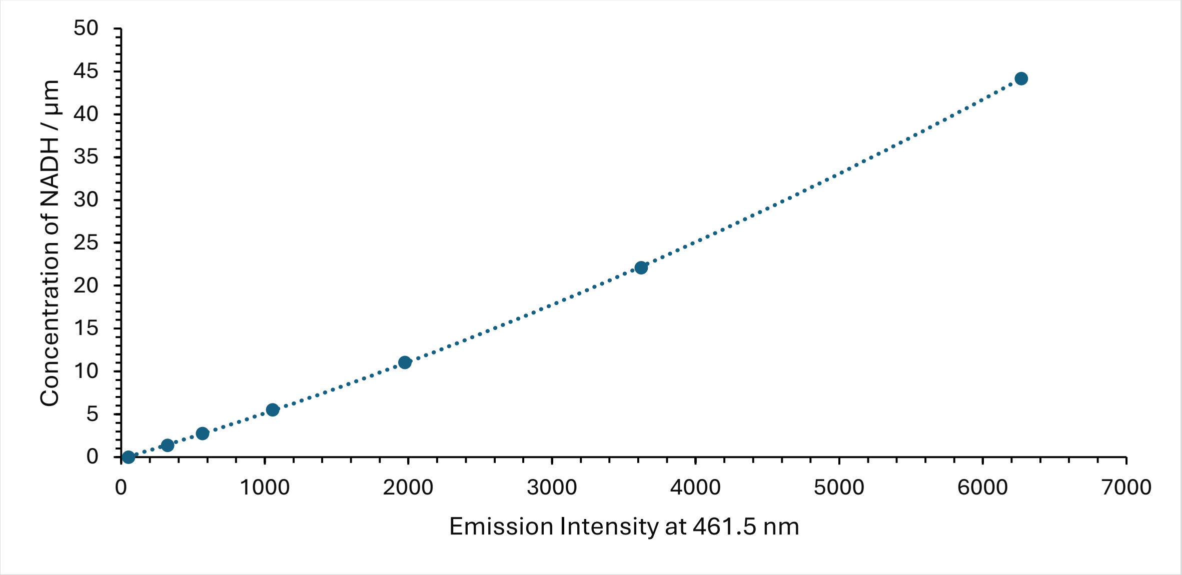

Figure S39: Polynomic trend between concentration and emission intensity of NADH in acetonitrile/water (1/2, v/v).

With this formula all concentrations of NADH in the solvent mixture acetonitrile/water (1/2, v/v) were calculated.

The turnover numbers (TONs) and turnover frequencies (TOFs) were calculated with the following formulas:

$$TON = \frac{c_{NADH}}{c_{CAT}} \qquad eq.\ 8$$

Here c_{NADH} is the concentration of NADH in μM and c_{CAT} is the concentration of the PS-CAT 2^{t}BuCzfDPPZRhCp*, which is 5 μM.

$$TOF = \frac{TON}{Time} \qquad eq.\ 9$$

Here, the TON is divided by the time in h.

6.2. Formate-driven Catalysis

Formate-driven catalysis was carried out in a sealable cuvette with a path length of 10 mm (quartz glass, Hellma Anaytics) in 3 mL solvent (acetonitrile/water (1/9, v/v)). The samples were prepared in an argon-filled glovebox under inert atmosphere with degassed solvents. The final concentrations are the following: 5 μM 2*t*BuCzDPPZRhCp*, 50 mM $NaHCO_2$, 250 μM NAD^+. In a certain time interval, fluorescence spectra were recorded to monitor the formation of NADH. For this, the cuvette was placed in a fluorescence spectrometer, and it was excited at 340 nm. Emission of NADH was observed at λ_{max} = 462.5 nm. Triplicates were measured to calculate an average TON.

RT No. 1 RT No. 2 RT No. 3

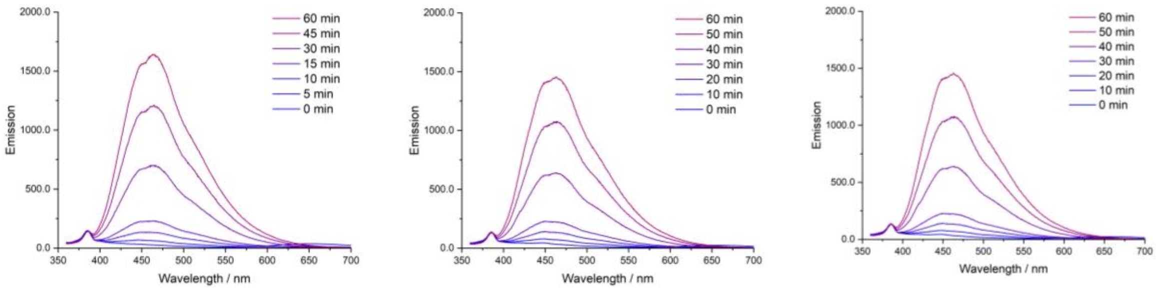

Figure S40: Fluorescence spectra of the catalytic mixture at room temperature measured after 0, 5, 10, 15, 30, 45, and 60 min.

45 °C No. 1 45 °C No. 2 45°C No. 3

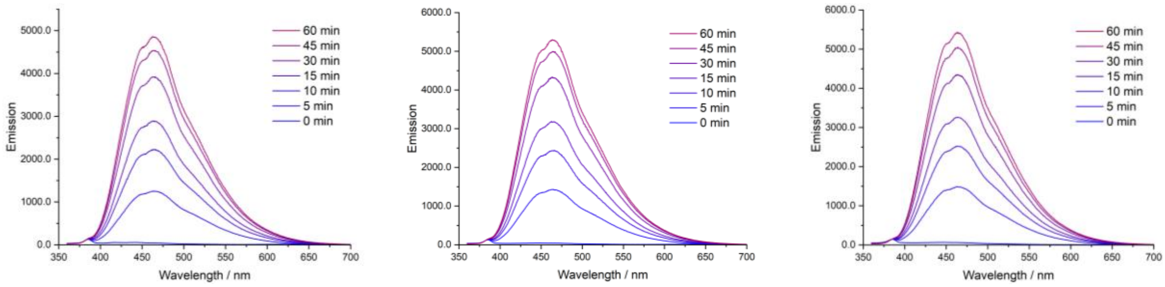

Figure S41: Fluorescence spectra of the catalytic mixture at 45 °C measured after 0, 5, 10, 15, 30, 45, and 60 min.

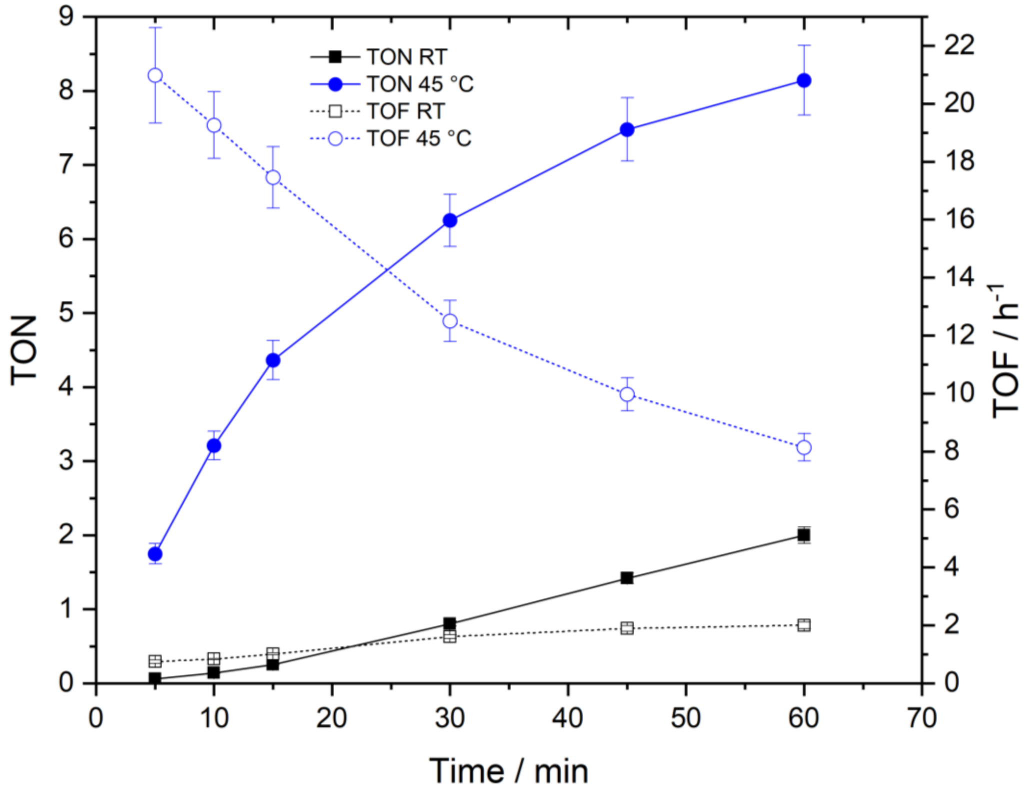

Figure S42: TON and TOF values of the format-driven catalysis at 45 °C and at room temperature.

6.3. Light-driven Catalysis

Light-driven catalysis was carried out in a sealable cuvette with a path length of 10 mm (quartz glass, Hellma Anaytics) in 3 mL solvent (acetonitrile/water (1/2, v/v)). The samples were prepared in a glovebox under inert atmosphere using degassed solvents. The final concentrations are the following: 5 µM 2^{t}BuCzDPPZRhCp*, 400 µM BIH-OMe, 250 µM NAD^+. In the case of triethylamine (TEA) as the sacrificial electron donor, it was used in a concentration of 0.212 M, and Na_2HPO_4 (0.17 M) was used to prepare a buffer. After preparation, an LED stick ((λ_{max} = 465 nm, fwhm = 20 nm, P = 45 mW/cm^2)) was placed under the cuvette, and the solution was irradiated for the indicated time. In regular time intervals, fluorescence spectra were recorded to monitor the formation of NADH. For this, the cuvette was placed in a fluorescence spectrometer, and it was excited at 340 nm. Emission of NADH was observed at λ_{max} = 461.5 nm. This procedure was repeated for a certain number of cycles. To prove that all components are needed for photocatalysis, conditions without irradiation and without catalyst were performed. Triplicates were measured to calculate an average TON.

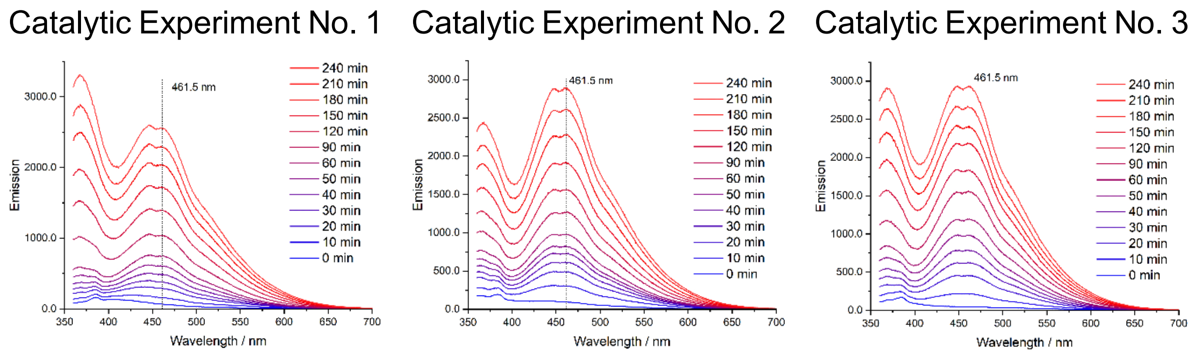

Figure S43: Fluorescence spectra of the catalytic mixture measured after 0, 10, 20, 30, 40, 50, 60, 90, 120, 150, 180, 210, and 240 min. Conditions: 5 µM 2^{t}BuCzDPPZRhCp*, 400 µM BIH-OMe, 250 µM NAD$^+$, LED (λ_{max} = 465 nm, fwhm = 20 nm, P = 45 mW/cm^2), acetonitrile/water (1/2, v/v).

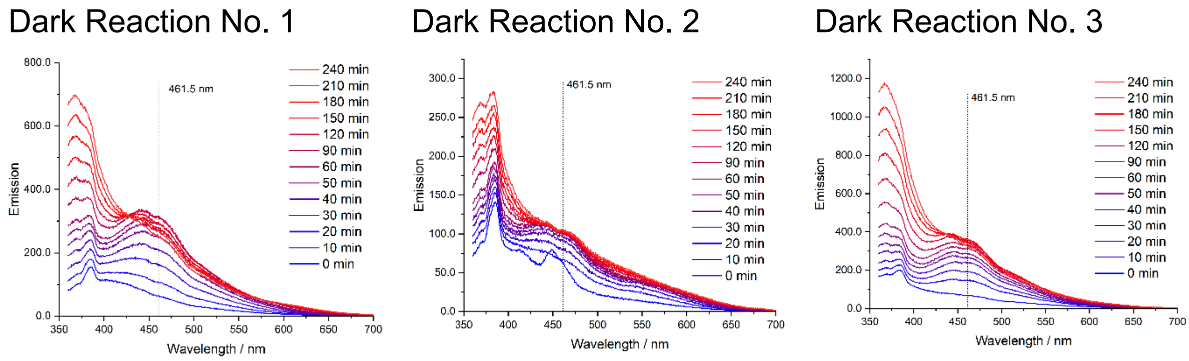

Figure S44: Fluorescence spectra of the dark reaction measured after 0, 10, 20, 30, 40, 50, 60, 90, 120, 150, 180, 210, and 240 min. Conditions: 5 µM 2^{t}BuCzDPPZRhCp*, 400 µM BIH-OMe, 250 µM NAD$^+$, acetonitrile/water (1/2, v/v).

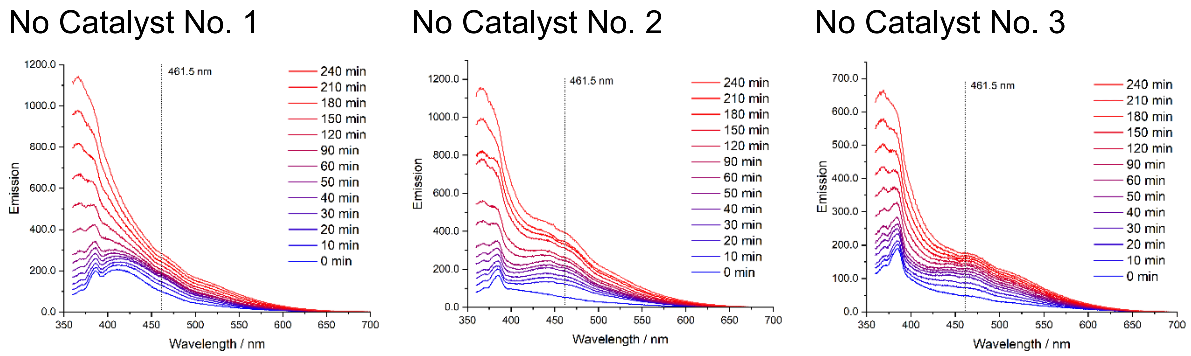

Figure S45: Fluorescence spectra of the mixture without catalyst measured after 0, 10, 20, 30, 40, 50, 60, 90, 120, 150, 180, 210, and 240 min. Conditions: 400 µM BIH-OMe, 250 µM NAD+, LED (λ_{max} = 465 nm, fwhm = 20 nm, P = 45 mW/cm^2), acetonitrile/water (1/2, v/v).

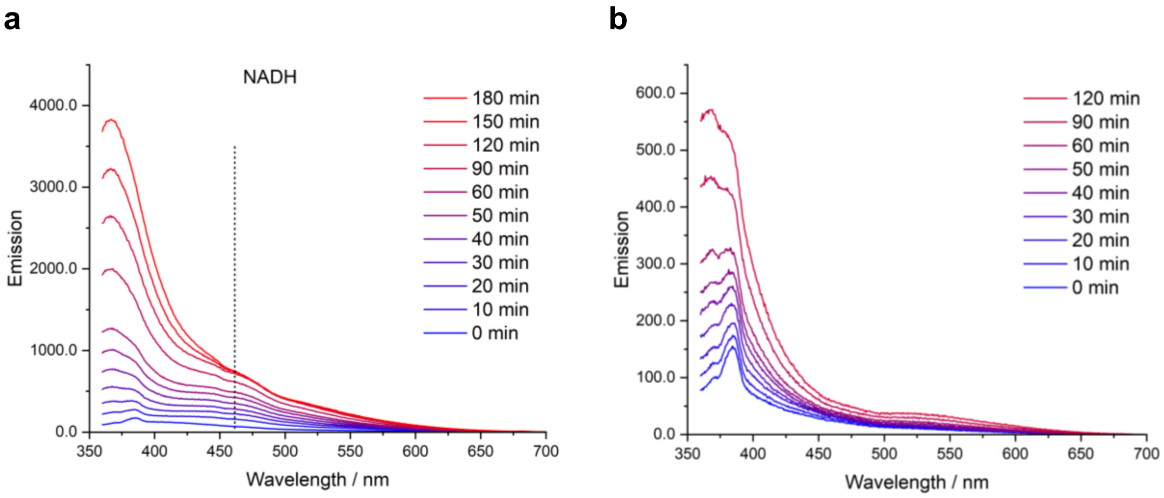

Figure S46. Formation of NADH monitored by fluorescence spectroscopy at 45 °C (5 µM 2^{t}BuCzDPPZRhCp*, 400 µM BIH-OMe, 250 µM NAD+, LED (λ_{max} = 465 nm, fwhm = 20 nm, P = 45 mW/cm^2), acetonitrile/water (1/2, v/v)) (**a**). Fluorescence spectra of a mixture only containing BIH-OMe (400 µM in acetonitrile/water (1/2, v/v)) under irradiation at 465 nm (fwhm = 20 nm, P = 45 mW/cm^2) (**b**).

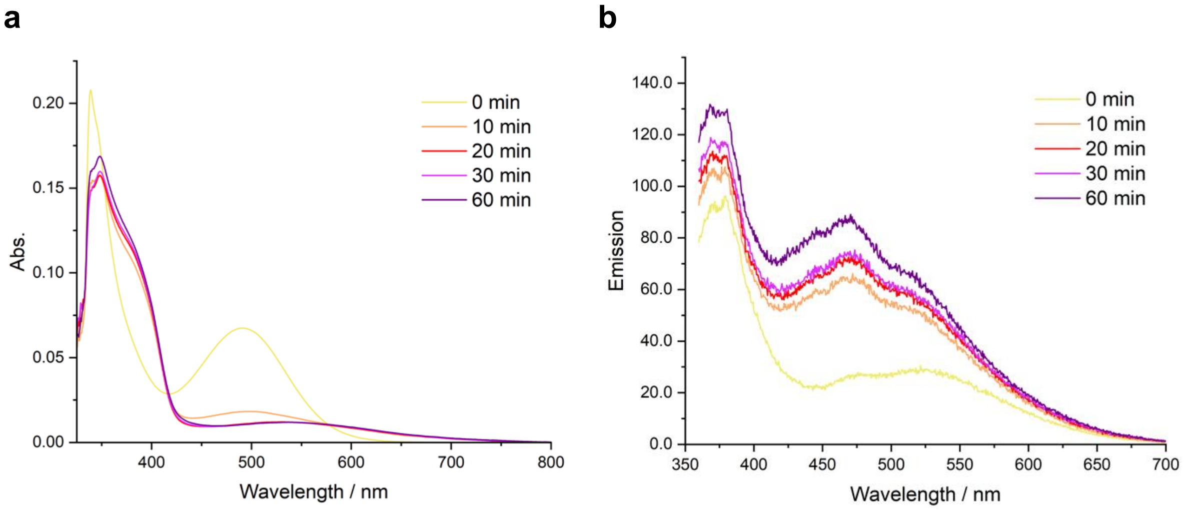

Figure S47: UV-Vis (**a**) and fluorescence (**b**) spectra of the catalytic mixture measured after 0, 10, 20, 30, 40, 50, and 60. Conditions: 5 μM 2^{t}BuCzDPPZRhCp*, 3300 μM BIH-OMe, 250 μM NAD$^+$, LED (λ_{max} = 465 nm, fwhm = 20 nm, P = 45 mW/cm^2), acetonitrile/water (1/2, v/v).

7. Computational Results

Table S2. Calculated singlet-triplet energy gaps at TDDFT level of theory, and derived intersystem crossing and fluorescence rate constants for 2^{t}BuCzDPPZ.

<table>
<tr><th></th><th colspan="2">State</th><th>ΔE / eV</th><th>k_{ISC} / s^{-1}</th><th>k_F (S_1) / s^{-1}</th></tr>
<tr><td rowspan="4">Acetonitrile</td><td rowspan="2">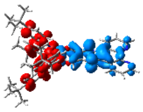
S_1, 2.24 eV, CT</td><td>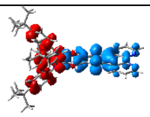
T_1, 1.84 eV, CT</td><td>0.40</td><td>4.8×10^6</td><td rowspan="2">6.8×10^5</td></tr>
<tr><td>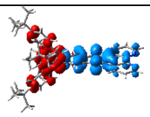
T_2, 2.12 eV, CT</td><td>0.12</td><td>3.5×10^7</td></tr>
<tr><td rowspan="2">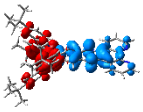
S_2, 2.34 eV, CT</td><td>T_1, 1.84 eV, CT</td><td>0.50</td><td>2.8×10^6</td><td rowspan="2">N.A.</td></tr>
<tr><td>T_2, 2.12 eV, CT</td><td>0.21</td><td>3.7×10^6</td></tr>
<tr><td rowspan="4">Toluene</td><td rowspan="2">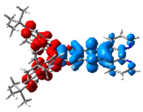
S_1, 2.47 eV, CT</td><td>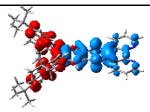
T_1, 2.22 eV, CT</td><td>0.25</td><td>1.0×10^7</td><td rowspan="2">1.1×10^6</td></tr>
<tr><td>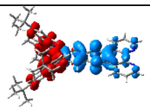
T_2, 2.30 eV, CT</td><td>0.17</td><td>2.4×10^7</td></tr>
<tr><td rowspan="2">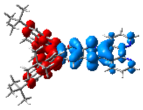
S_2, 2.49 eV, CT</td><td>T_1, 2.22 eV, CT</td><td>0.26</td><td>2.8×10^6</td><td rowspan="2">N.A.</td></tr>
<tr><td>T_2, 2.30 eV, CT</td><td>0.18</td><td>3.7×10^6</td></tr>
</table>

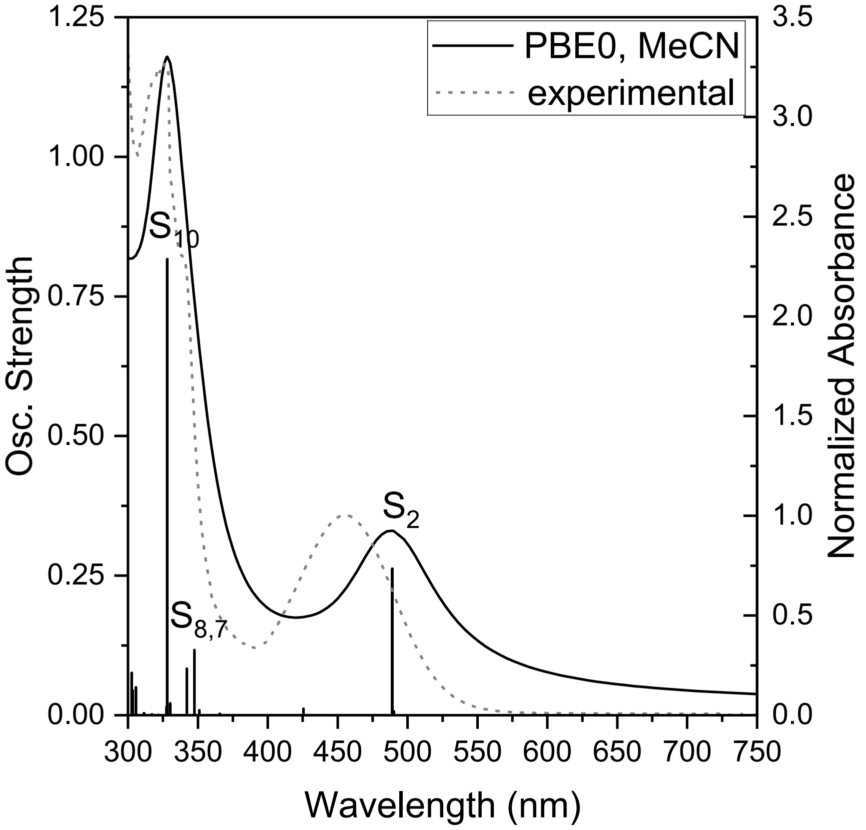

Figure S48. Simulated UV/Vis absorption spectrum (solid black line) of 2^{t}BuCzDPPZ in acetonitrile; key electronic excitations contributing to the absorption are indicated. The transitions were broadened by Gaussian functions with a full width at half maximum of 0.2 eV. The experimental spectrum is given in dashed line.

Table S3. Simulated vertical excitation energies (E^e), wavelengths (λ), oscillator strengths (f), and singly-excited configurations of the singlet-singlet transitions visualized by means of charge density difference (CDD) plots involved in the initial absorption of 2^{t}BuCzDPPZ in the S_0 equilibrated geometry (in acetonitrile).

State	Transition Type	CDD	E^e / eV	λ / nm	f
S_1	CT	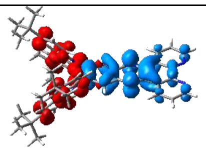	2.53	490	0.007
S_2	CT	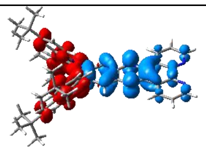	2.54	489	0.263
S_7	CT, LE	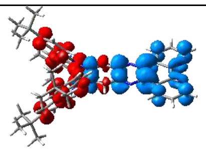	3.57	347	0.117
S_8	CT, LE	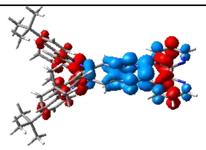	3.62	342	0.083
S_{10}	LE, CT	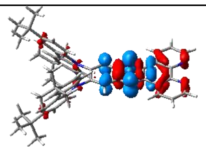	3.78	328	0.817

Table S4. Simulated vertical excitation energies (E^e), wavelengths (λ), oscillator strengths (f), and singly-excited configurations of the singlet-triplet transitions visualized by means of charge density difference (CDD) plots involved in the initial absorption of 2^{t}BuCzDPPZ in the S_0 equilibrated geometry (in acetonitrile).

State	Transition Type	CDD	E^e / eV	λ / nm	f	s^2
T_1	CT	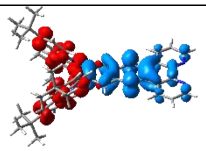	2.27	546	0.000	2.00
T_2	CT	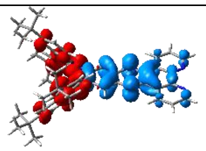	2.35	527	0.000	2.00
T_3	CT, LE	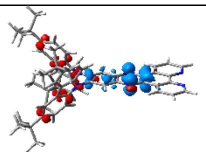	2.84	437	0.000	2.00
T_4	CT	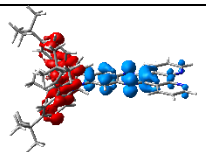	2.89	429	0.000	2.00
T_5	LE	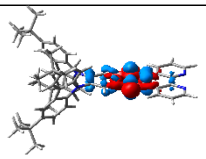	2.91	426	0.000	2.00

Table S5. Spin-orbit couplings of first 5 singlet and 5 triplet states of 2^{t}BuCzDPPZ given in cm^{-1}, at TDA-DFT level of theory in acetonitrile.

**State, Energy, CDD**	T_1, 2.27 eV 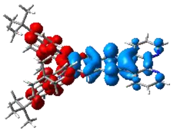	T_2, 2.35 eV 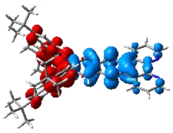	T_3, 2.84 eV 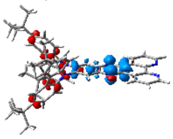	T_4, 2.89 eV 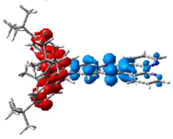	T_5, 2.91 eV 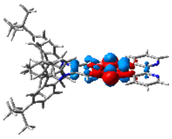
S_0, 0.00 eV	1.04	0.57	1.06	0.39	0.42
S_1, 2.53 eV 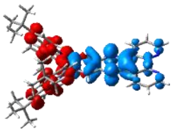	0.42	0.42	0.20	0.24	2.39
S_2, 2.54 eV 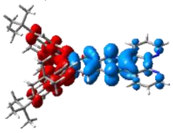	0.40	0.21	0.39	0.00	1.57
S_3, 2.91 eV 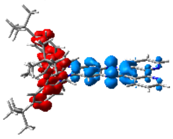	0.37	0.07	0.33	0.01	0.26
S_4, 2.99 eV 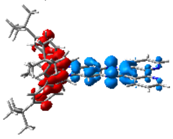	0.04	0.30	0.19	0.18	0.85
S_5, 3.29 eV 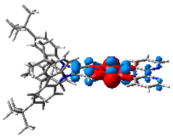	6.50	1.70	5.82	0.31	3.09

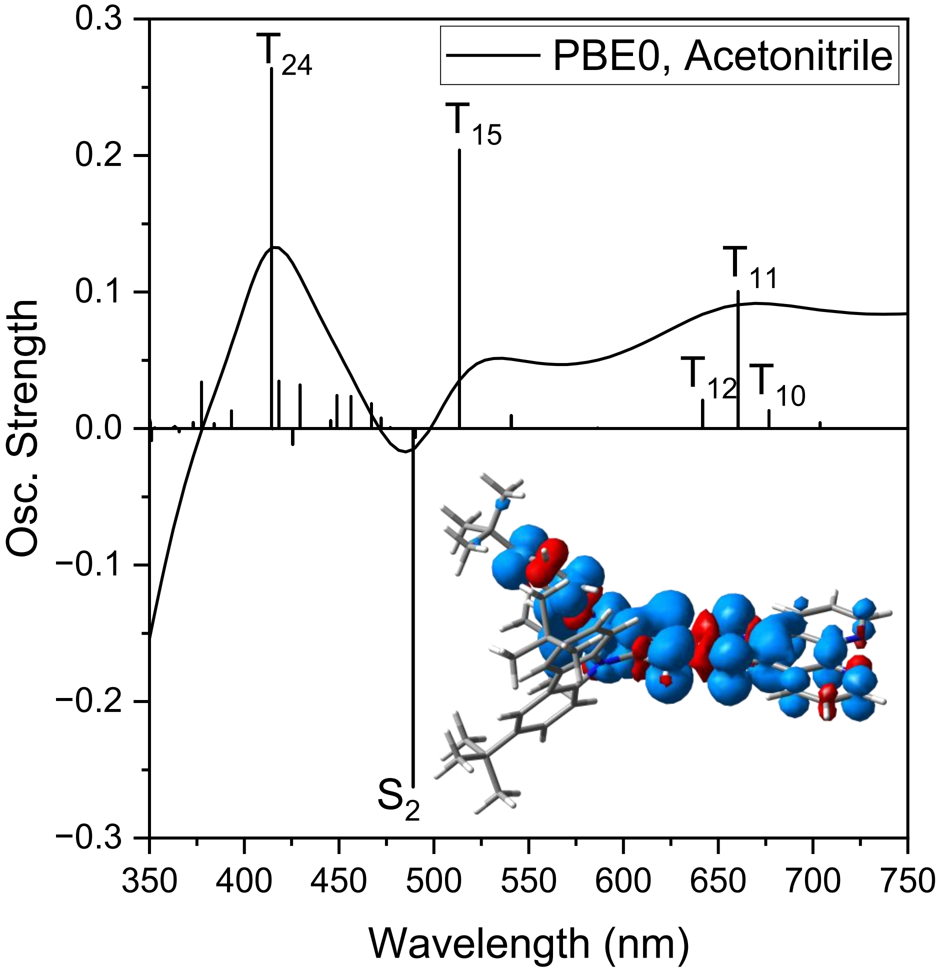

Figure S49. Simulated transient absorption spectrum of 2^{t}BuCzDPPZ in acetonitrile; key electronic excitations contributing to the absorption are indicated. The transitions were broadened by Gaussian functions with a full width at half maximum of 0.2 eV. Spin density of the triplet state is displayed.

Table S6. Simulated vertical excitation energies (E^e), wavelengths (λ), oscillator strengths (f), and singly-excited configurations visualized by means of charge density difference (CDD) plots of the spin and dipole-allowed triplet-triplet transitions involved in the excited-state absorption of 2^{t}BuCzDPPZ within the equilibrated T_1 geometry (in acetonitrile).

State	Transition Type	CDD	E^e / eV	λ / nm	f	s^2
T_5	CT, LE	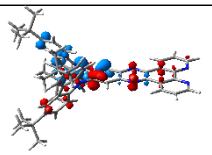	1.08	1148	0.319	2.04
T_{10}	CT, LE	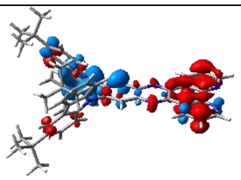	1.83	677	0.013	2.05
T_{11}	LE, CT	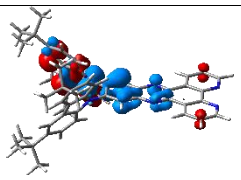	1.88	660	0.100	2.05
T_{12}	CT	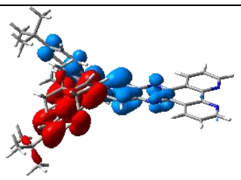	1.93	642	0.021	2.04
T_{15}	LE	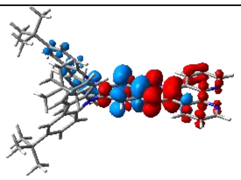	2.41	514	0.204	2.05
T_{24}	CT, LE	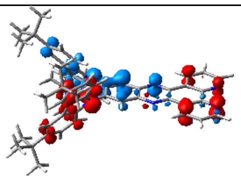	2.99	414	0.264	2.09

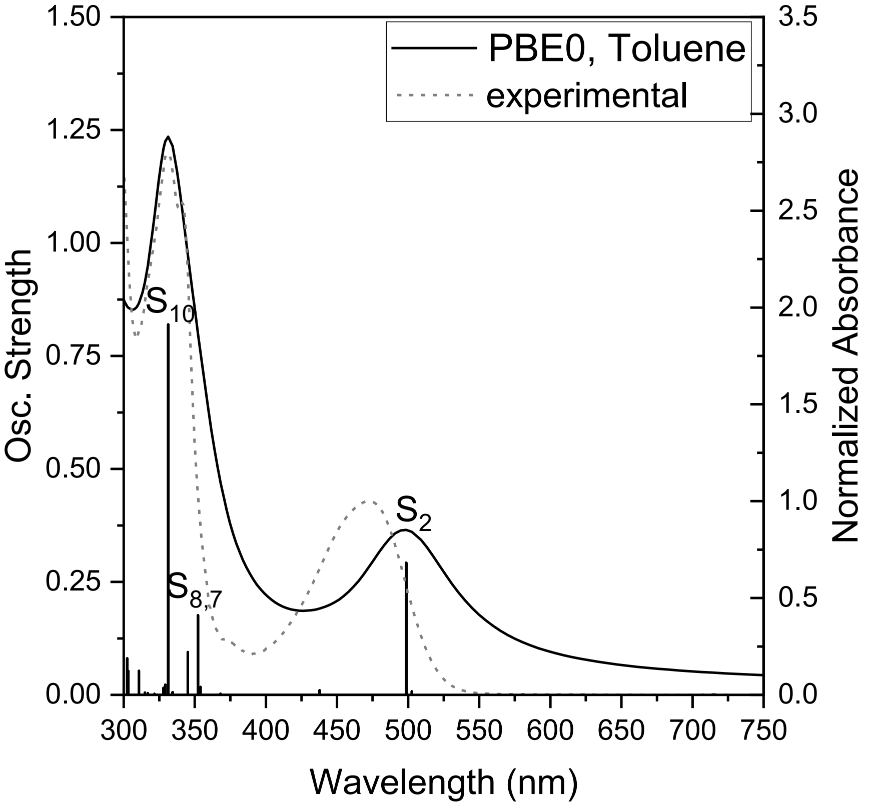

Figure S50. Simulated UV/Vis absorption spectrum (solid black line) of 2^{t}BuCzDPPZ in toluene; key electronic excitations contributing to the absorption are indicated. The transitions were broadened by Gaussian functions with a full width at half maximum of 0.2 eV. The experimental spectrum is given in dashed line.

Table S7. Simulated vertical excitation energies (E^e), wavelengths (λ), oscillator strengths (f), and singly-excited configurations of the singlet-singlet transitions visualized by means of charge density difference (CDD) plots involved in the initial absorption of 2^{t}BuCzDPPZ in the S_0 equilibrated geometry (in toluene).

State	Transition Type	CDD	E^e / eV	λ / nm	f
S_1	CT	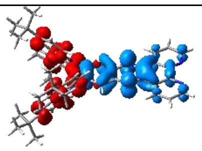	2.47	503	0.008
S_2	CT	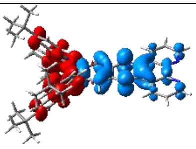	2.49	499	0.293
S_7	CT, LE	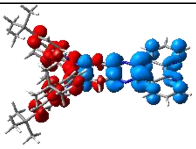	3.52	352	0.176
S_8	CT, LE	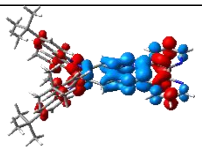	3.59	345	0.095
S_{10}	LE, CT	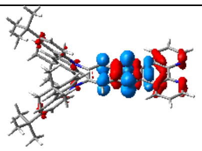	3.74	331	0.820

Table S8. Simulated vertical excitation energies (E^e), wavelengths (λ), oscillator strengths (f), and singly-excited configurations of the singlet-triplet transitions visualized by means of charge density difference (CDD) plots involved in the initial absorption of 2^{t}BuCzDPPZ in the S_0 equilibrated geometry (in toluene).

State	Transition Type	CDD	E^e / eV	λ / nm	f	s^2
T_1	CT	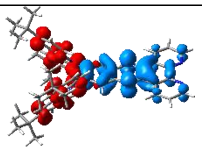	2.22	558	0.000	2.00
T_2	CT	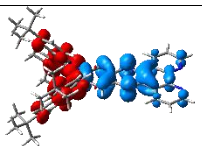	2.30	538	0.000	2.00
T_3	CT	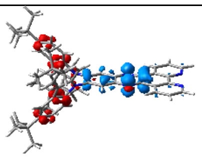	2.81	441	0.000	2.00
T_4	CT	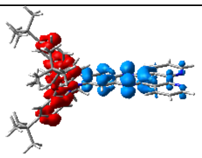	2.82	440	0.000	2.00
T_5	LE	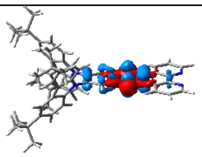	2.89	429	0.000	2.00

Table S9. Spin-orbit couplings of first 5 singlet and 5 triplet states of 2^{t}BuCzDPPZ given in cm^{-1}, at TDA-DFT level of theory in toluene.

	T_1, 2.22 eV 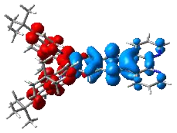	T_2, 2.30 eV 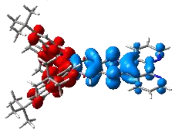	T_3, 2.81 eV 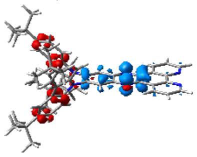	T_4, 2.82 eV 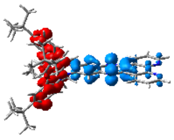	T_5, 2.89 eV 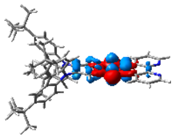
S_0, 0.00 eV	1.11	0.60	1.08	0.40	0.33
S_1, 2.47 eV 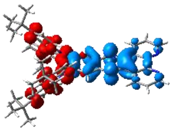	0.39	0.44	0.12	0.22	2.43
S_2, 2.49 eV 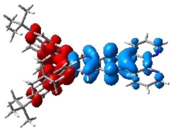	0.42	0.19	0.40	0.03	1.52
S_3, 2.83 eV 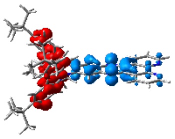	0.35	0.06	0.31	0.00	0.25
S_4, 2.91 eV 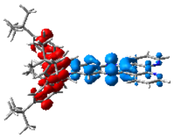	0.01	0.26	0.18	0.15	0.78
S_5, 3.37 eV 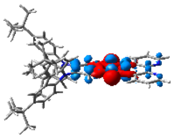	6.21	1.68	5.59	0.29	2.59

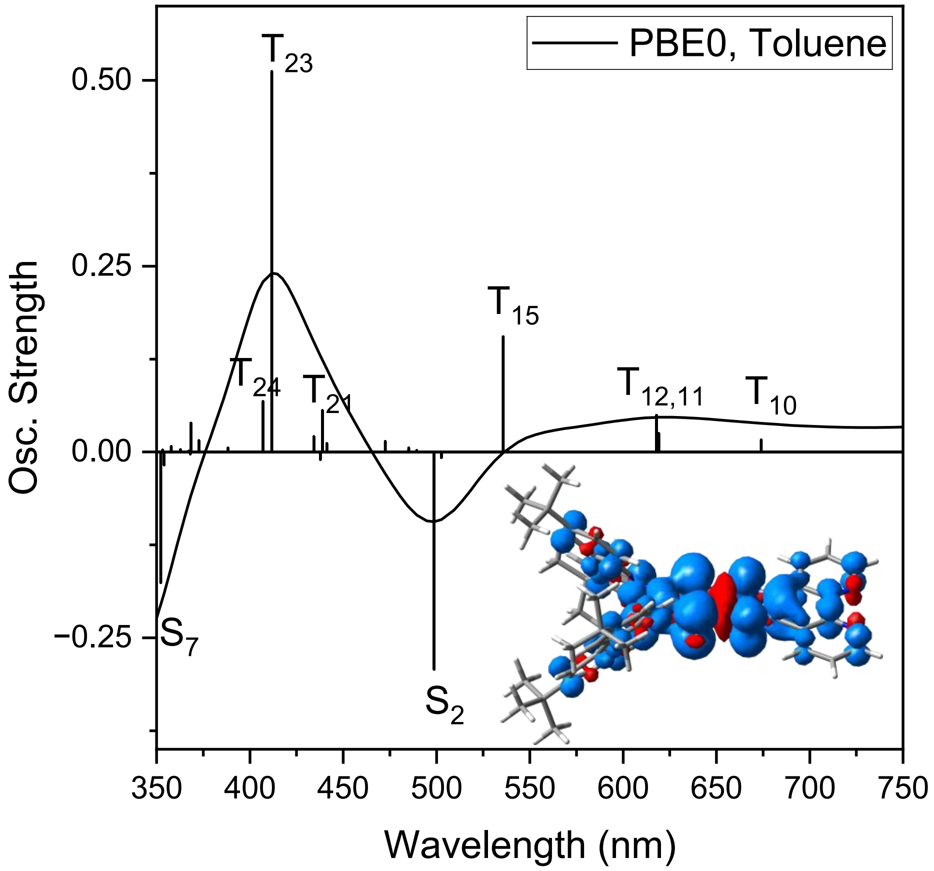

Figure S51. Simulated transient absorption spectrum of 2^{t}BuCzDPPZ in toluene; key electronic excitations contributing to the absorption are indicated. The transitions were broadened by Gaussian functions with a full width at half maximum of 0.2 eV. Spin density of the triplet state is displayed.

Table S10. Simulated vertical excitation energies (E^e), wavelengths (λ), oscillator strengths (f), and singly-excited configurations visualized by means of charge density difference (CDD) plots of the spin and dipole-allowed triplet-triplet transitions involved in the excited-state absorption of 2^{t}BuCzDPPZ within the equilibrated T_1 geometry (in toluene).

State	Transition Type	CDD	E^e / eV	λ / nm	f	s^2
T_4	CT	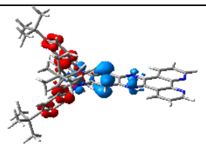	0.99	1251	0.241	2.04
T_{10}	LE	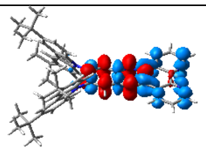	1.84	674	0.016	2.08
T_{11}	CT	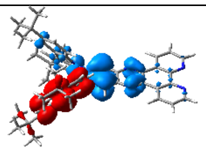	2.00	619	0.025	2.05
T_{12}	CT	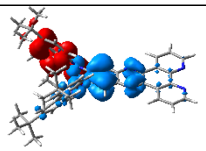	2.01	618	0.049	2.05
T_{15}	LE	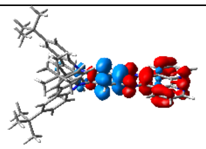	2.31	536	0.155	2.04
T_{21}	CT	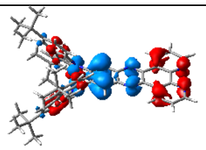	2.83	439	0.056	2.05
T_{23}	CT	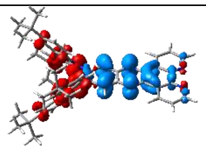	3.01	412	0.512	2.06
T_{24}	LE	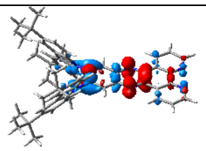	3.05	407	0.068	2.11

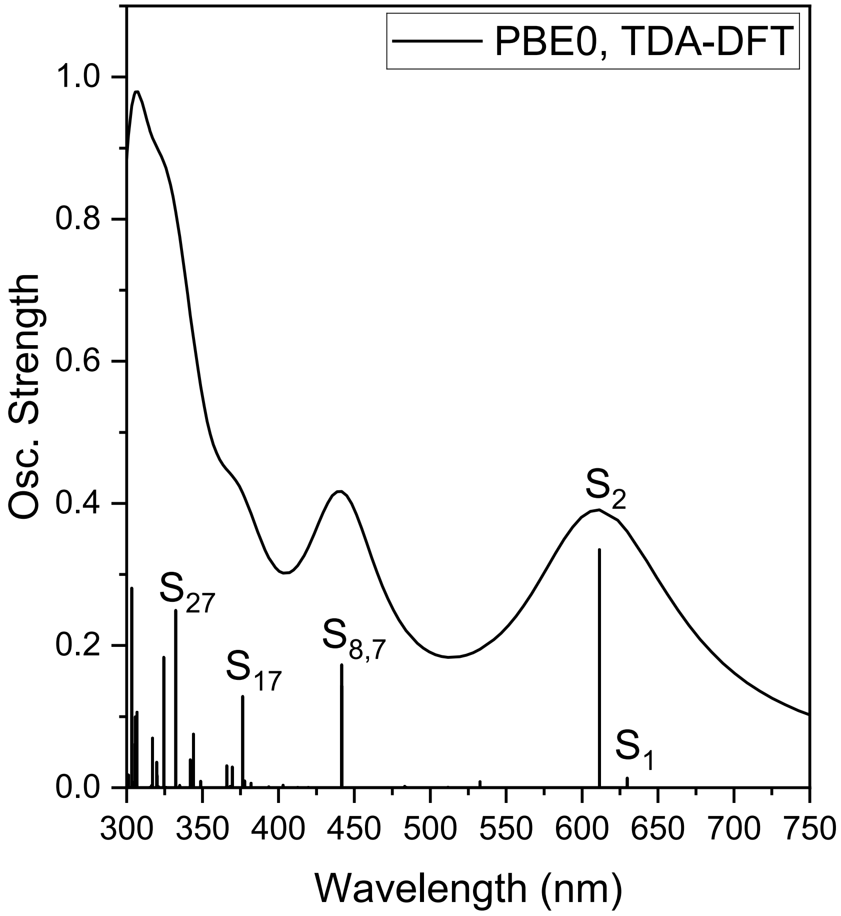

Figure S52. Simulated UV/Vis absorption spectrum (solid black line) of 2^{t}BuCzDPPZRhCp* in acetonitrile calculated at TDA-DFT level of theory with PBE0 functional; key electronic excitations contributing to the absorption are indicated. The transitions were broadened by Gaussian functions with a full width at half maximum of 0.2 eV.

Table S11. Simulated vertical excitation energies (E^e), wavelengths (λ), oscillator strengths (f), and singly-excited configurations of the singlet-singlet transitions visualized by means of charge density difference (CDD) plots involved in the initial absorption of 2^{t}BuCzDPPZRhCp* in the S_0 equilibrated geometry at TDA-DFT level of theory with PBE0 functional.

State	Transition Type	CDD	E^e / eV	λ / nm	f
S_1	LLCT	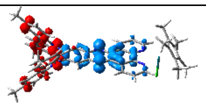	1.97	630	0.014
S_2	LLCT	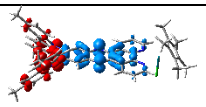	2.03	611	0.335
S_7	LLCT	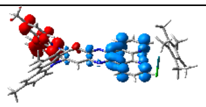	2.81	442	0.173
S_8	LLCT	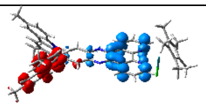	2.81	442	0.143
S_{17}	MLCT	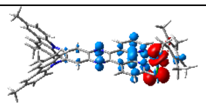	3.29	377	0.128
S_{27}	MLCT	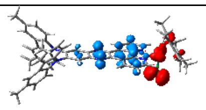	3.73	332	0.249

Table S12. Simulated vertical excitation energies (E^e), wavelengths (λ), oscillator strengths (f), and singly-excited configurations of the singlet-triplet transitions visualized by means of charge density difference (CDD) plots involved in the initial absorption of 2^{t}BuCzDPPZRhCp* in the S_0 equilibrated geometry at TDA-DFT level of theory with PBE0 functional.

State	Transition Type	CDD	E^e / eV	λ / nm	f	s^2
T_1	LLCT, ILCT	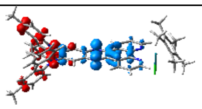	1.80	690	0.000	2.00
T_2	LLCT	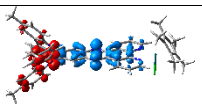	1.83	677	0.000	2.00
T_3	LLCT	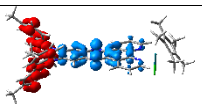	2.31	536	0.000	2.00
T_4	LLCT	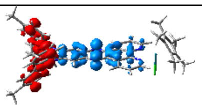	2.40	516	0.000	2.00
T_5	LLCT	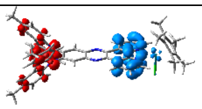	2.53	489	0.000	2.00

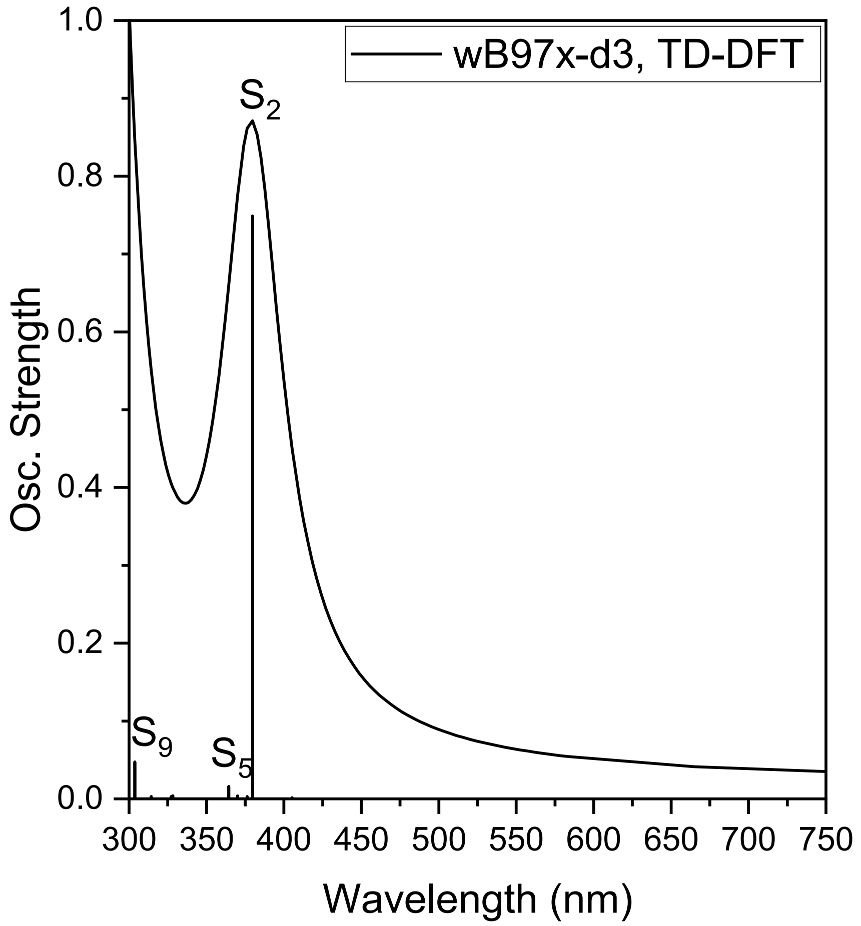

Figure S53. Simulated UV/Vis absorption spectrum (solid black line) of 2*t*BuCzDPPZRhCp* in toluene calculated at TD-DFT level of theory with ωB97-d3 functional; key electronic excitations contributing to the absorption are indicated. The transitions were broadened by Gaussian functions with a full width at half maximum of 0.2 eV.

Table S13. Simulated vertical excitation energies (E^e), wavelengths (λ), oscillator strengths (f), and singly-excited configurations of the singlet-singlet transitions visualized by means of charge density difference (CDD) plots involved in the initial absorption of 2^tBuCzDPPZRhCp* in the S_0 equilibrated geometry at TD-DFT level of theory with ωB97-d3 functional.

State	Transition Type	CDD	E^e / eV	λ / nm	f
S_1	MC	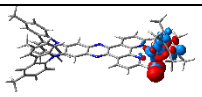	3.06	405	0.002
S_2	LLCT	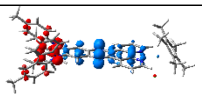	3.26	374	0.749
S_5	LMCT	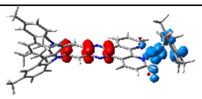	3.40	361	0.016
S_9	LLCT	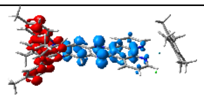	4.08	303	0.047

Table S14. Simulated vertical excitation energies (E^e), wavelengths (λ), oscillator strengths (f), and singly-excited configurations of the singlet-triplet transitions visualized by means of charge density difference (CDD) plots involved in the initial absorption of 2^tBuCzDPPZRhCp* in the S_0 equilibrated geometry at TD-DFT level of theory with ωB97-d3 functional.

State	Transition Type	CDD	E^e / eV	λ / nm	f	s^2
T_1	ILCT	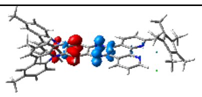	2.27	545	0.000	2.00
T_2	MC	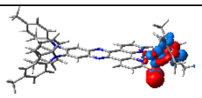	2.60	477	0.000	2.00
T_3	MC	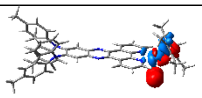	2.68	462	0.000	2.00
T_4	LLCT	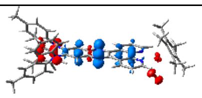	2.82	439	0.000	2.00
T_5	LLCT	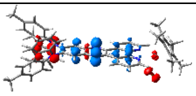	2.91	425	0.000	2.00

Table S15. Spin-orbit couplings of first 5 singlet and 5 triplet states of 2*t*BuCzDPPZRhCp* given in cm^{-1}, at TD-DFT level of theory in toluene.

**State, Energy, CDD**	T_1, 2.27 eV 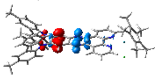	T_2, 2.60 eV 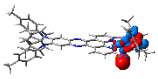	T_3, 2.68 eV 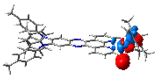	T_4, 2.82 eV 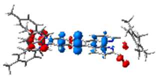	T_5, 2.91 eV 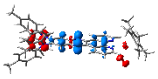
S_0, 0.00 eV	3.29	926.74	875.74	62.60	1239.25
S_1, 3.06 eV 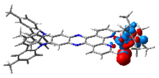	7.00	31.33	761.33	18.85	1006.64
S_2, 3.26 eV 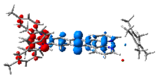	1.24	104.75	1.34	0.48	1.58
S_3, 3.29 eV 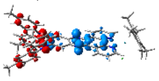	0.66	3.23	2.83	0.50	2.78
S_4, 3.35 eV 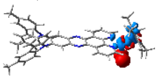	5.14	756.33	21.85	2.61	51.98
S_5, 3.40 eV 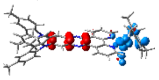	2.89	805.48	34.74	8.77	52.85

Table S16. Simulated vertical excitation energies (E^e), wavelengths (λ), oscillator strengths (f), and singly-excited configurations of the singlet-singlet transitions visualized by means of charge density difference (CDD) plots involved in the initial absorption of 2^{t}BuCzDPPZRhCp* in the S_0 equilibrated geometry at TDA-DFT level of theory with ωB97-d3 functional.

State	Transition Type	CDD	E^e / eV	λ / nm	f
S_1	MC	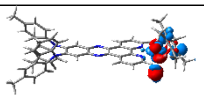	3.09	402	0.002
S_2	LLCT	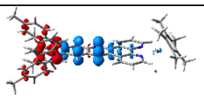	3.31	374	0.745
S_5	MC	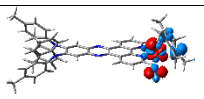	3.44	361	0.017
S_9	LLCT	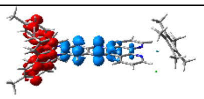	4.09	303	0.042

Table S17. Simulated vertical excitation energies (E^e), wavelengths (λ), oscillator strengths (f), and singly-excited configurations of the singlet-triplet transitions visualized by means of charge density difference (CDD) plots involved in the initial absorption of 2^{t}BuCzDPPZRhCp* in the S_0 equilibrated geometry at TDA-DFT level of theory with ωB97-d3 functional.

State	Transition Type	CDD	E^e / eV	λ / nm	f	s^2
T_1	MC	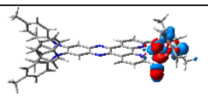	2.65	467	0.000	2.00
T_2	ILCT	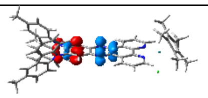	2.70	460	0.000	2.00
T_3	MC	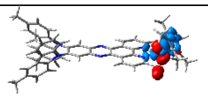	2.76	450	0.000	2.00
T_4	ILCT	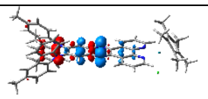	2.91	426	0.000	2.00
T_5	MC	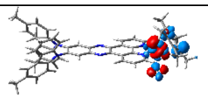	2.97	418	0.000	2.00

8. Additional Figure

a b

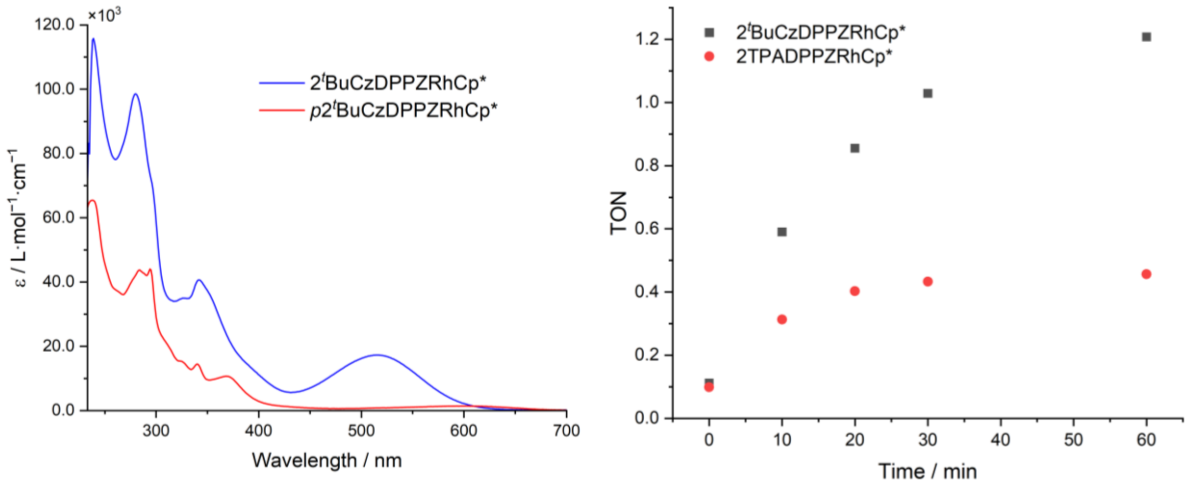

Figure S54. UV/Vis absorption spectra of 2^{t}BuCzDPPZRhCp* and *p*2^{t}BuCzDPPZRhCp* in $CHCl_3$ (a). TON values for the following conditions in acetonitrile/water (1/2, v/v): 5 μM 2^{t}BuCzDPPZRhCp* or *p*2^{t}BuCzDPPZRhCp*, 400 μM BIH-OMe, 250 μM NAD^+, LED (λ_{max} = 465 nm, fwhm = 20 nm, P = 45 mW/cm^2) (b).

9. Literature

[1] T. Igarashi, E. Tayama, H. Iwamoto, E. Hasegawa, “Carbon–carbon bond formation via benzoyl umpolung attained by photoinduced electron-transfer with benzimidazolines” *Tetrahedron Letters* **2013**, *54*, 6874–6877.

[2] R. Siebert, D. Akimov, M. Schmitt, A. Winter, U. S. Schubert, B. Dietzek, J. Popp, “Spectroscopic Investigation of the Ultrafast Photoinduced Dynamics in π-Conjugated Terpyridines” *ChemPhysChem* **2009**, *10*, 910–919.

[3] J. Kübel, R. Schroot, M. Wächtler, U. S. Schubert, B. Dietzek, M. Jäger, “Photoredox-active Dyads Based on a Ru(II) Photosensitizer Equipped with Electron Donor or Acceptor Polymer Chains: A Spectroscopic Study of Light-Induced Processes toward Efficient Charge Separation” *J. Phys. Chem. C* **2015**, *119*, 4742–4751.

[4] C. Müller, T. Pascher, A. Eriksson, P. Chabera, J. Uhlig, “KiMoPack: A python Package for Kinetic Modeling of the Chemical Mechanism” *J. Phys. Chem. A* **2022**, *126*, 4087–4099.

[5] B. Dietzek, T. Pascher, V. Sundström, A. Yartsev, “Appearance of coherent artifact signals in femtosecondtransient absorption spectroscopy in dependence on detector design” *Laser Phys. Lett.* **2007**, *4*, 38–43.

[6] F. Neese, “Software update: The ORCA program system—Version 5.0” *WIREs Computational Molecular Science* **2022**, *12*, e1606.

[7] K. Shizu, H. Kaji, “Quantitative prediction of rate constants and its application to organic emitters” *Nat Commun* **2024**, *15*, 4723.

[8] M. Ernzerhof, G. E. Scuseria, “Assessment of the Perdew–Burke–Ernzerhof exchange-correlation functional” *The Journal of Chemical Physics* **1999**, *110*, 5029–5036.

[9] C. Adamo, V. Barone, “Toward reliable density functional methods without adjustable parameters: The PBE0 model” *The Journal of Chemical Physics* **1999**, *110*, 6158–6170.

[10] F. Weigend, “Accurate Coulomb-fitting basis sets for H to Rn” *Phys. Chem. Chem. Phys.* **2006**, *8*, 1057.

[11] F. Weigend, R. Ahlrichs, “Balanced basis sets of split valence, triple zeta valence and quadruple zeta valence quality for H to Rn: Design and assessment of accuracy” *Phys. Chem. Chem. Phys.* **2005**, *7*, 3297.

[12] Daniel Escudero, Adèle D. Laurent, Denis Jacquemin, *Time-Dependent Density Functional Theory: A Tool to Explore Excited States. In: Leszczynski, J. (eds) Handbook of Computational Chemistry*, Springer, **2015**.

[13] M. J. G. Peach, M. J. Williamson, D. J. Tozer, “Influence of Triplet Instabilities in TDDFT” *J. Chem. Theory Comput.* **2011**, *7*, 3578–3585.

[14] G. V. Loukova, V. P. Vasiliev, A. A. Milov, V. A. Smirnov, V. I. Minkin, “Unraveling electronic properties of an organometallic solute: Lippert-Mataga and quantum-chemical extensive study” *Journal of Photochemistry and Photobiology A: Chemistry* **2016**, *327*, 6–14.

[15] K. Witas, S. S. Nair, T. Maisuradze, L. Zedler, H. Schmidt, P. Garcia-Porta, A. S. J. Rein, T. Bolter, S. Rau, S. Kupfer, B. Dietzek-Ivanšić, D. U. Sorsche, “Beyond the First Coordination Sphere─Manipulating the Excited-State Landscape in Iron(II) Chromophores with Protons” *J. Am. Chem. Soc.* **2024**, *146*, 19710–19719.

[16] C. Wegeberg, D. Häussinger, S. Kupfer, O. S. Wenger, “Controlling the Photophysical Properties of a Series of Isostructural d^6 Complexes Based on Cr^0 , Mn^I , and Fe^{II}” *J. Am. Chem. Soc.* **2024**, *146*, 4605–4619.

[17] N. Sinha, J. Wellauer, T. Maisuradze, A. Prescimone, S. Kupfer, O. S. Wenger, "Reversible Photoinduced Ligand Substitution in a Luminescent Chromium(0) Complex" *J. Am. Chem. Soc.* **2024**, *146*, 10418–10431.
[18] L. Zedler, S. Kupfer, H. Schmidt, B. Dietzek-Ivanšić, "Oxidation-state sensitive light-induced dynamics of Ruthenium-4H-Imidazole complexes" *Chemistry – A European Journal* **2024**, *30*, e202303079.
[19] F. Neese, F. Wennmohs, A. Hansen, U. Becker, "Efficient, approximate and parallel Hartree–Fock and hybrid DFT calculations. A 'chain-of-spheres' algorithm for the Hartree–Fock exchange" *Chemical Physics* **2009**, *356*, 98–109.
[20] R. Izsák, F. Neese, W. Klopper, "Robust fitting techniques in the chain of spheres approximation to the Fock exchange: The role of the complementary space" *The Journal of Chemical Physics* **2013**, *139*, 094111.
[21] B. Helmich-Paris, B. De Souza, F. Neese, R. Izsák, "An improved chain of spheres for exchange algorithm" *The Journal of Chemical Physics* **2021**, *155*, 104109.
[22] K. Shizu, H. Kaji, "Theoretical Determination of Rate Constants from Excited States: Application to Benzophenone" *J. Phys. Chem. A* **2021**, *125*, 9000–9010.
[23] D. P. Craig, T. Thirunamachandran, *Molecular Quantum Electrodynamics: An Introduction to Radiation-molecule Interactions*, Academic Press Inc, **1998**.
[24] G. E. Shillito, T. B. J. Hall, D. Preston, P. Traber, L. Wu, K. E. A. Reynolds, R. Horvath, X. Z. Sun, N. T. Lucas, J. D. Crowley, M. W. George, S. Kupfer, K. C. Gordon, "Dramatic Alteration of^3 ILCT Lifetimes Using Ancillary Ligands in $[Re(L)(CO)_3$ (phen-TPA)$]^{n+}$ Complexes: An Integrated Spectroscopic and Theoretical Study" *J. Am. Chem. Soc.* **2018**, *140*, 4534–4542.
[25] G. E. Shillito, D. Preston, P. Traber, J. Steinmetzer, C. J. McAdam, J. D. Crowley, P. Wagner, S. Kupfer, K. C. Gordon, "Excited-State Switching Frustrates the Tuning of Properties in Triphenylamine-Donor-Ligand Rhenium(I) and Platinum(II) Complexes" *Inorg. Chem.* **2020**, *59*, 6736–6746.
[26] G. E. Shillito, D. Preston, J. D. Crowley, P. Wagner, S. J. Harris, K. C. Gordon, S. Kupfer, "Controlling Excited State Localization in Bichromophoric Photosensitizers via the Bridging Group" *Inorg. Chem.* **2024**, *63*, 4947–4956.
[27] J. J. Sutton, D. Preston, P. Traber, J. Steinmetzer, X. Wu, S. Kayal, X.-Z. Sun, J. D. Crowley, M. W. George, S. Kupfer, K. C. Gordon, "Excited-State Switching in Rhenium(I) Bipyridyl Complexes with Donor–Donor and Donor–Acceptor Substituents" *J. Am. Chem. Soc.* **2021**, *143*, 9082–9093.
[28] G. Yang, G. E. Shillito, C. Zens, B. Dietzek-Ivanšić, S. Kupfer, "The three kingdoms—Photoinduced electron transfer cascades controlled by electronic couplings" *The Journal of Chemical Physics* **2023**, *159*, 024109.
[29] V. García-López, J. V. Milić, M. Zalibera, D. Neshchadin, M. Kuss-Petermann, L. Ruhlmann, J. Nomrowski, N. Trapp, C. Boudon, G. Gescheidt, O. S. Wenger, F. Diederich, "Light-actuated resorcin[4]arene cavitands" *Tetrahedron* **2018**, *74*, 5615–5626.
[30] D. Gudeika, A. Miasojedovas, O. Bezvikonnyi, D. Volyniuk, A. Gruodis, S. Jursenas, J. V. Grazulevicius, "Differently substituted benzothiadiazoles as charge-transporting emitters for fluorescent organic light-emitting diodes" *Dyes and Pigments* **2019**, *166*, 217–225.
[31] B. Schäfer, H. Görls, M. Presselt, M. Schmitt, J. Popp, W. Henry, J. G. Vos, S. Rau, "Derivatives of dipyrido[3,2-a:2′,3′-c]phenazine and its ruthenium complexes, influence of arylic substitution on photophysical properties" *Dalton Trans.* **2006**, 2225–2231.

[32] B. S. Adams, G. E. Shillito, H. van der Salm, R. Horvath, C. B. Larsen, X.-Z. Sun, N. T. Lucas, M. W. George, K. C. Gordon, “Alteration of Intraligand Donor–Acceptor Interactions Through Torsional Connectivity in Substituted Re-dppz Complexes” *Inorg. Chem.* **2017**, *56*, 12967–12977.
[33] L. Zedler, P. Wintergerst, A. K. Mengele, C. Müller, C. Li, B. Dietzek-Ivanšić, S. Rau, “Outpacing conventional nicotinamide hydrogenation catalysis by a strongly communicating heterodinuclear photocatalyst” *Nat Commun* **2022**, *13*, 2538.
[34] J. Brückmann, C. Müller, I. Friedländer, A. K. Mengele, K. Peneva, B. Dietzek-Ivansic, S. Rau, “Photocatalytic Reduction of Nicotinamide Co-factor by Perylene Sensitized RhII IComplexes**” *Chem. Eur. J.* **2022**, *23*, e202201931.
